# Acknowledgment to the Reviewers of *Sensors* in 2022

**DOI:** 10.3390/s23031179

**Published:** 2023-01-19

**Authors:** Sensors Editorial Office

**Affiliations:** MDPI AG, St. Alban-Anlage 66, 4052 Basel, Switzerland

High-quality academic publishing is built on rigorous peer review. *Sensors* was able to uphold its high standards for published papers due to the outstanding efforts of our reviewers. Thanks to the efforts of our reviewers in 2022, the median time to first decision was 16 days and the median time to publication was 38 days. Regardless of whether the articles they examined were ultimately published, the editors would like to express their appreciation and thank the following reviewers for the time and dedication that they have shown *Sensors*:

A. D. PrasadLiang LuA. K. M. Mahbubur RahmanLiang WangA. Ping ZhangLiang XiaoAakash AhmadLiang XuAaqif Afzaal AbbasiLiang ZouAarón Ángel Salas-SánchezLiang-Bi ChenAaron D. LikensLiangcai CaoAaron J. PungLiang-Cheng ShiuAaron K. HoshideLiangcheng TuAbas SabouniLianggui LiuAbayomi OtebolakuLiangjiang YuAbbas HaddadiLiangjing YangAbbas NasriLiangliang ChengAbbas OrandLiangliang LuAbbas RohaniLiangliang XiaoAbbas SultanLiangliang YangAbbass NasserLiangpeng GaoAbdelaziz A. AbdelhamidLiangtian WanAbdelaziz TimesliLiangxing HuAbdel-Baset M. A. IbrahimLianjin HongAbdeldjalil Aïssa-El-BeyLianqun ZhouAbdelhafez DalabLiansheng SuiAbdelhak MerizigLianyong QiAbdelhaleem KhaderLianyou JingAbdelhalim BencheikhLianzhou ChenAbdelhameed IbrahimLibera AmentaAbdelhamid HelaliLibin YangAbdelhamied Ashraf AteyaLibo ZhaoAbdelkader AbderrahmaneLibor PekařAbdelkader ShaabanLichao LiuAbdellah BoulouzLichao ZhangAbdellah ChehriLida HengAbdelmalik MoujahidLidia Buda-OzogAbdel-Mehsen AhmadLidia DobrescuAbdel-Nasser SharkawyLidia PutlyaevaAbdel-Rahman AklLidia Sánchez GonzálezAbdelrahman ElsaidLidija MandicAbdelrazek ElnasharLien Minh DangAbderrahim MoumenLieselotte CortenAbderrahmane AissaLieu-Hen ChenAbderrazak BoutramineLifeng WangAbderrazak JemaiLifeng WuAbdessamad DidiLifeng ZhangAbdollah Kavousi-FardLifeng ZhuAbdon SepulvedaLihong LiAbdu GumaeiLihua LiuAbdu SaifLihua WangAbdul HafeezLihui WangAbdul Hafez Abdul HafezLijun LiuAbdul Kayum Mohammad Zakir HossainLijun WangAbdul MajeedLili Nemec ZlatolasAbdul Mannan KhanLilia TightizAbdul MatinLilian SosaAbdul MuhithLiliana CostaAbdul QayyumLiliana Mihaela MogaAbdul Salam ShahLi-ling HungAbdul WahidLiliya DemidovaAbdulateef BalogunLiliya S. LogoydaAbdulaziz AborujilahLilly LiAbdulaziz AlsulamiLilong ChaiAbdulaziz M. AbdulazizLim ByeonghwaAbdulbast AbushgraLiming HuangAbdulfatah A. YusufLiming WangAbdulhalim DandoushLiming YaoAbdulkader JoukhadarLiming ZhangAbdullah AlamoodiLin ChengAbdullah AlomariLin DongAbdullah BadeLin LuAbdullah BeyazLin MengAbdullah Bin ShamsLin QiAbdullah ÇakanLin WangAbdullah LakhanLin WuAbdullatif ShuhailLin XuAbdulnaser M. AlshoaibiLin YangAbdulrahman Khalaf Al-AliLin ZhouAbdülvahap ÇakmakLinas PetkevičiusAbdulwahed M. AboukarimaLinas StripinisAbdur KhanLinas SvilainisAbdur Rahman Bin ShahidLinchuan ZhaoAbdur RaziqLincoln Faria Da SilvaAbdzahir ElnourLinda Azevedo KauppilaAbebaw Belay JemereLinda PaternòAbebe Belay AdegeLinda SenigagliesiAbel Silva-FilhoLinda Viola Ehlin CaldasAbel Yeboah-OforiLindsay BottomsAbera TulluLinfei YinAbhijit MondalLinfeng LiAbhilash ChandelLing LuoAbhilash Puthanveettil MadathilLing MaAbhilasha SinghLing YuAbhimanyu Singh GarhwalLingfu KongAbhimanyu ThakurLingju MengAbhishek GhoshLingjun ZhaoAbhishek KandwalLingling HuAbhishek KaushikLingling LiAbhishek KumarLingmei JiangAbhishek SharmaLingqian ZhangAbhrajit SenguptaLingwei XuAbiel Aguilar-GonzálezLingxiang ZhengAbimbola E. OluwalanaLingxin ChenAbiodun AbioyeLingyun LuAbla SmahiLingze DuanAbolfazl MohebbiLingzhong FanAbolfazl Safaei BezgabadiLingzhong GuoAbraham Boby RibyLinko G. NikolovAbraham FinnyLinlin LuAbraham López VivancosLinlin YouAbraham Mejia AguilarLino MisogutiAbram MusinguziLinping ZhaoAbror BuriboevLinqing GuiAbu Saleh Md BakibillahLinqing LuoAbu Sayed ChowdhuryLinquan BaiAbu SufianLinsen XuAbu Zaharin AhmadLinsheng HuoAbul BasharLionel NkenyereyeAca JovanovićLip Yee PorAcácio M. R. AmaralLiping DuAccardo AgostinoLiping QiAchmad SolikhinLiping XieAda FortLiqiang LuoÁdám BárdosLiquan GuoAdam BujnowskiLiqun LiuAdam C. CobbLisandro LovisoloAdam CwudzińskiLise BellangerAdam DeptułaLise KilicAdam GlowaczLiseane Padilha ThivesAdam JablonskiLitong LyuÁdám JuhászLiu WangAdam KhanLiu YangAdam KłodowskiLiudmila Gerasimova-MeigalAdam KowalewskiLiuqing YangAdam Krzysztof PilatLívia Florio SgobbiAdam KrzyzakLivia PetrescuAdam L. KaczmarekLivija CveticaninAdam LeckoLivio D′AlviaAdam M. KawalecLiviu GorasAdam MartowiczLiviu-Adrian CotfasAdam MilikLiviu-Daniel ȘtefanAdam RosińskiLiwei AnAdam RutkowskiLi-Wei LiuAdam Szela̧gLixia YangAdam ZagubieńLixin WuAdam ZiębińskiLixing WangAdamantia StamouLiying ZhengAdão SilvaLiyu YeAdawiya J. HaiderLizhi WangAddisson SalazarLizhu LiAdeeb SalhLjubomir VračarAdeela HanifLobo LouieAdel A. BahaddadLogan T. TrujilloAdel Ali AhmedLoganathan VeeramuthuAdel Shaaban AwadLokam AnjaneyuluAdela BadauLokendra SinghAdelaide MirandaLong ChenAdelina BordianuLong HanAdem OzcelikLong JinAdhimar OliveiraLong LiuAdil IllahiLong WangAdina ArvinteLong WeiAdina Carmen IlieLong WuAdirak KanchanaharuthaiLongfei ZhouAdit KurniawanLonghua DingAdithya BalasubramanyamLongxiang GuoAditya Kumar SahuLoredana SimčićAditya P. MathurLorena Elena MelitAditya Rio PrabowoLorena ParraAditya SahuLorena Redondo-MorataAdmir MulahusićLorena Reyes-RubianoAdnan GutubLorena RossiAdnan MahmoodLorena Velazquez-IbarraAdnan MujahidLorenzo BrognaraAdnan Shahid KhanLorenzo DianaAdnan Ul-HasanLorenzo DiniaAdnane HabibLorenzo GraziAdnane NoualLorenzo L. ColumboAdnen KacemLorenzo MonfardiniAdolfo FernándezLorenzo PiazzoAdolfo Josue Rodriguez-RodriguezLorenzo PiccoAdonios KarpetisLorenzo PutzuAdrian Amor-MartinLorenzo Ricciardi CelsiAdrian BurlacuLorenzo ScaleraAdrian DziembowskiLorenzo ServaAdrian Elmi-TeranderLoris NanniAdrian GligorLotfi Ben SaidAdrian IoanaLouis F. M. Ten BoschAdrian J. T. TeoLourdes María Fernández SeguínAdrian KampaLourenco PereiraAdrian KliksLovorka LibrićAdrian KnapczykLu BaiAdrian Martinez-RivasLu LengAdrián Mateo-OrcajadaLu RenAdrian PetrovanLu YinAdrian PopLuana De Freitas NascimentoAdrian Turek RahoveanuLuana OlivieriAdriana GrigorescuLuay TahaAdriana MexicanoLubna Luxmi DhiraniAdriana MoreaLubomir BenaAdrian-Mihail StoicaĽuboš KrišťákAdriano CampsĽuboš OvseníkAdriano Chaves LisboaĽuboslav StrakaAdriano de Oliveira AndradeLuc EstebanezAdrien Chan-Hon-TongLuca AbeniAdroaldo RaizerLuca Andrea LudovicoAfaq AhmadLuca AntognoliAfaque Manzoor SoomroLuca BraidottiAffan YasinLuca BruzzoneAfonso Rangel Garcez de AzevedoLuca CavaggioniAfshin AkhshaniLuca D′AciernoAfshin ShoeibiLuca FredianelliAfzal BadshahLuca GuarneraAfzal Husain KhanLuca MagistrelliAfzal KhanLuca MartinoAgata Ando′Luca MassiddaAgata GiełczykLuca OggioniAgata Krywko-CendrowskaLuca OnetoAgata Marta SocciniLuca PaduaAgbotiname Lucky ImoizeLuca PalmieriAggeliki VlachostergiouLuca PancioniAghil ShavalipourLuca Paolo ArdigòAgilandeeswari LoganathanLuca PetrignaAgne Paulauskaite-TarasevicieneLuca PolettoAgnes PurwidyantriLuca RussoAgnes Sui-Yin ChanLuca SchirruAgnese BrunziniLuca SciacovelliAgnese SbrolliniLuca TavasciAgnieszka AdamczukLuca TestarelliAgnieszka ChodorekLuca UlrichAgnieszka DudziakLucas CambuimAgnieszka GenerowiczLucas De Oliveira TeixeiraAgnieszka Kijo-KleczkowskaLucas EncarnaçãoAgnieszka KłopotowskaLucas FerreiraAgnieszka MlynarskaLucas Hernández-SaraviaAgnieszka PregowskaLucas PereiraAgnieszka RichertLucia MacchiusiAgnieszka SękalaLucia TrozzoAgnieszka StacherzakLucian LazarescuAgnieszka SujakLucian PetrescuAgnieszka Szmelter-JaroszLucian Pîslaru-DănescuAgnieszka WosiakLucian TrifinaAgnieszka Wujeska-KlauseLuciana R. P. KassabAgostino ForestieroLucian-Gabriel ZamfirAguinaldo FraddosioLucian-Mihai CosovanuAgustí Costa-JoverLuciano BertiniAgustín Aibar AlmazánLuciano L. MenegaldoAgustín Díaz-ÁlvarezLuciano NoceraAgustin L. Herrera-MayLucica BarbesAgustina BuccellaLucija BrezočnikAgustinus Purna IrawanLucija VejmelkaAhad BehboodiLucile SautotAhana Roy ChoudhuryLucilla AlfonsiAhed AbugabahLucjan GucmaAhlam Al-DhamariLucjan KozielskiAhmad AlkhodreLucjan SetlakAhmad AlshormanLudo M. MahieuAhmad Beng Hong KuehLudovic MacaireAhmad DarkiLudovico ValliAhmad Esmaili TorshabiLudovit KovanicAhmad Fairuz OmarLuigi BenedicentiAhmad FarooqLuigi BibbòAhmad H. Juma′hLuigi BorzìAhmad HamzehLuigi CampanellaAhmad HassanLuigi CatuognoAhmad JahanbakhshiLuigi CelonaAhmad KeshavarzLuigi de NapoliAhmad MahmoodLuigi FortunaAhmad MohammadLuigi MeccarielloAhmad Sayed FayazLuigi Pio PrencipeAhmad SerjoueiLuigi SequinoAhmad Taher AzarLuigi TiburziAhmad Zia Ul-Saufie Mohamad JaperiLuigi ZanziAhmadjan MuhammadhajiLuis Adrián Zúñiga AvilésAhmed A. IbrahimLuis Alberto Morales RosalesAhmed A. KishkLuís Álvarez SabucedoAhmed AbdelaalLuis AriasAhmed Abdelmottaleb OmarLuis Arturo SorianoAhmed Abdelraheem FarghalyLuis Augusto SilvaAhmed Al-ArajiLuis Barba-GuamanAhmed Aly Hussein AlyLuís BarretoAhmed ArafaLuis Bernal EscobedoAhmed BadrLuís C. CoelhoAhmed BarnawiLuis CadarsoAhmed BelaadiLuis CarúsAhmed Chiheb AmmariLuis E. Gomez ArmasAhmed El BaroudyLuis Edmundo Lugo UribeAhmed El OualkadiLuis Enrique González-JiménezAhmed ElmogyLuis F. PedrazaAhmed ElnakibLuis Fernando HerránAhmed ELsawafLuis Fernando Luque-VegaAhmed El-SayedLuís Filipe Lages MartinsAhmed F. KineberLuis Francisco DíezAhmed GomaaLuis García-AsenjoAhmed HammamiLuis Gerardo Trujillo-FrancoAhmed HelmiLuis GómezAhmed ImteajLuís Gonzaga Mendes MagalhãesAhmed Ismail Mohamed ShahinLuis HernandezAhmed Jamal Abdullah Al-GburiLuis Ignacio Lopera GonzálezAhmed K. BadawiLuis JacintoAhmed KhwajaLuis Javier Sánchez-AparicioAhmed M. A. SayedLuis M. Álvarez-SabucedoAhmed M. EbidLuis M. G. AbegãoAhmed Mancy MosaLuis Manuel Fernández De AhumadaAhmed N. AbdallaLuis MariscalAhmed SabryLuis Mendez-BarrosoAhmed Shaharyar KhwajaLuis Norberto López De LacalleAhmet Cagdas SeckinsLuís OliveiraAhmet Emin AktanLuís PáduaAhmet Ercan TopcuLuis ParrillaAhmet Fatih TabakLuis PayáAhmet KaraküçükLuís PinhoAhmet KertmenLuís Pinto Da SilvaAhmet Mete VuralLuís RosaAhmet YaziciLuís RoseiroAhmet Yigit ArabulLuis SigchaAhmet YildizLuis ValentínAhteshamul HaqueLuis VelascoAi IshidaLuisiana SabbatiniAida MustaphaLuiz Carlos Gomes De FreitasAidel SalihLuiz GonçalvesAiert Amundarain IrizarLuiz Henrique Alves De MedeirosAijaz AbbasiLuiz Palucci-VieiraAila KhanLuiz PoffoAilan CheLuka FlegarAileni Raluca MariaLuka PavićAimé Lay-EkuakilleLuka PavličAimen KhacharemLuka RumoraAimin ChangLukáš KrauzAintzane ArmentiaLukasz BonenbergAirat Zh. SakhabutdinovŁukasz GorskiAisha NazirLukasz JedlinskiAisheng WuLukasz KaczmarekAivars VembrisŁukasz KłosowskiAjay BandiŁukasz KnypińskiAjay Kumar YagatiŁukasz KolimasAjay PalŁukasz MakowskiAjaya Kumar SinghLukasz MalekAjit BeheraŁukasz NagiAjith AbrahamLukasz PrzybylAjith Tom JamesLukasz PyrzowskiAkash Kumar BhoiŁukasz RauchAkbar AliŁukasz SadowskiAkbar Hojatti-NajafabadiLukasz SójkaAkbar MohammadŁukasz StaszewskiAkebo YamakamiŁukasz WiechetekAkhil GuptaŁukasz WięcławAkhil KandhariŁukasz ŻyłkaAkhilesh PathakLulu WangAkhilesh ThyagaturuLuminita GhervaseAkhouri Pramod KrishnaLuminita MarmureanuAkhtar NaureenLuminita NicolescuAkihiko FujiwaraLuminita OanceaAkihiko KatayamaLuminita ScripcariuAkil AhmadLun GaoAkilarasan MuthumariappanLuna NgeljaratanAkilu Yunusa-KaltungoLung-Chi LeeAkinori ItoLung-Chien ChenAkinrinade George AyankojoLuning BiAkio KurodaLuong Vuong NguyenAkio NakashimaLuqi WangAkio TsunedaLuqman Ali ShahAkira SasakiLu-Sheng HsiehAkira UmemuraLuyao ZhengAkos OdryM. A. Hannan Bin AzharAkrivi AsimakopoulouM. Firoz MridhaAkshansh GuptaM. G. SumithraAkshansh MishraM. H. AliabadiAkshansha ChauhanM. HassaballahAku VisuriM. KarthigaAlaa Abd-Alazeez Abu-SrhanM. Mary Shanthi RaniAlaa Abdulhady JaberM. NagamadhuAlain DieterlenM. SaravananAlain ReineixM. Shamim MiahAlain Richard NdjiongueM. SupriyaAlami AnouarM. Syed AliAlan LitchfieldM. V. A. BahubalendruniAlan-Miguel ValdezM. Ziyan SheriffAlanoud SubahiMa KeAlba Roda-SalesMabao LiuAlban KuriqiMabel VazquezAlbert E. PattersonMaciej DobrzańskiAlbert Garcia-BenadiMaciej HarasAlbert PrinnMaciej HenzelAlbert SabbanMaciej KubonAlbert Thomas AnastasioMaciej LawrynczukAlberto A. Escobar-PuentesMaciej MatuszewskiAlberto AraujoMaciej NikodemAlberto BarontiniMaciej RoskoszAlberto BattistelMaciej SadowskiAlberto BonastreMaciej SobierajAlberto Camarero OriveMaciej StefańczykAlberto Comesaña-CamposMaciej SułowiczAlberto De CastroMaciej ZaborowiczAlberto DoriaMadalina DumitriuAlberto FerranteMădălina Simona BălțatuAlberto GonzalezMaddaka ReddeppaAlberto GregoriMaddalena MantovaniAlberto Izquierdo-FuenteMadhav PatilAlberto J. Rosales SilvaMadhavi PaliAlberto NannarelliMadhumita SahooAlberto OchoaMadis RatasseppAlberto OlmoMagdalena B. SkarżyńskaAlberto PortaMagdalena Elizabeth Bergés TiznadoAlberto Rodriguez MartinezMagdalena KrbotAlberto RoncagliaMagdalena Maj-ZurawskaAlberto TellaecheMagdalena MatuszakAlberto VillalongaMagdalena MieloszykAldo CalcanteMagdalena StobieckaAldo GhisiMagdalena Valentina LunguAldo QuattroneMagdalena WlodarskaAlejandro C. FreryMagdalena WyrwickaAlejandro CaballeroMagdy ElbahnasawyAlejandro Galán-MercantMaggie MashalyAlejandro Garcia-Miranda FerrariMaghsoodi Tilaki Mohammad JavadAlejandro Gutiérrez-GilesMagno Conceição Das MercêsAlejandro JavaloyesMagnus FalkAlejandro MedinaMaha DrissAlejandro Ramírez-RojasMahanth PrasadAlejandro RincónMahdi BamdadAlejandro Roman LoeraMahdi BaradaranniaAlejandro RosadoMahdi JampourAlejandro SalcidoMahdi KhosravyAlejandro VegaMahdi NaghshvarianjahromiAleksandar Dj ValjarevićMahdi ShariatiAleksandar MiladinovicMaher KhalielAleksandar MiltenovicMaher Yahya SalloomAleksandar RadonjićMahesh Kumar RathiAleksandar SušićMahesh P. BhatAleksandar TrifunovićMaheshwari Mukesh KumarAleksandar ZivkovicMahima Agumbe SureshAleksander JakimowiczMahmood Abbasi LayeghAleksander NawratMahmood Al ShareedaAleksander SešekMahmood ShafieeAleksandr BugayMahmoud AbughoushAleksandr OmetovMahmoud BakrAleksandr RomanovMahmoud BendaryAleksandr S. AnikinMahmoud DhimishAleksandr SakhnevychMahmoud EbrahimiAleksandra JungMahmoud ElbattahAleksandra K. Eberhard-MoscickaMahmoud El-MorshedyAleksandra LabusMahmoud ElshariefAleksandra NinaMahmoud ElsisiAleksandra Radomska-ZalasMahmoud F. SeleimanAleksandra RadulovićMahmoud Nabil MahmoudAleksandra S. KristoMahmoud R. M. AtallaAleksandra StuparMahmoud RagabAleksandra SzydłowskaMahmoud ZadehbagheriAleksandras VoicikasMahmudur RahmanAleksandrs MarininsMahnoosh KholghiAleksei KiselevMahyar KhorasaniAleksei RozhnovMai The VuAleksei TitovMaiara De Jesus BassiAleksei V. EmelianovMaike SchwammbergerAleksei Vorob′evMaira Beatriz Hernandez MoranAleksejs ZacepinsMaitreyee WairagkarAleksey A. VedyaginMaja CavicAleksey FilippovMaja KrčumAleksey GvozdarevMaja PušnikAleksey KabanovMaja ŠtulaAleksey S. AnuchinMajed AbuseifAlena VimmrováMajid AliAlenka VagaskaMajid HossienpourAleš ProcházkaMajid KhazaeeAleš SlívaMajid MahmoodAleš StražeMajid Masteri-FarahaniAleš ŠvigeljMaju KuriakoseAlessandra BattistiMakhsudsho G. NematovAlessandra BiancolilloMakiko KobayashiAlessandra BorghiMakoto EndoAlessandra GalliMakram JafarAlessandra GiulianiMaksymilian MądzielAlessandra PutrinoMakusu TsutsuiAlessandro BazziMalaiyalathan AdimoolamAlessandro BoglioloMalarvizhi ArulrajAlessandro CaponeMalaya Kumar NathAlessandro CasavolaMalek GassoumiAlessandro CataniaMalena EspanolAlessandro CostantiniMałgorzata A. JanikAlessandro CuttinMałgorzata B. PańkowskaAlessandro D′EmilioMałgorzata CharytanowiczAlessandro Enrico Cesare RedondiMalgorzata DominoAlessandro FantiMałgorzata I. MichalczykAlessandro FedeleMałgorzata KujawskaAlessandro FilippeschiMałgorzata ŁabańskaAlessandro GabrielliMałgorzata MatusiakAlessandro LabellaMałgorzata Plechawska-WójcikAlessandro MalobertiMałgorzata Przybyła-KasperekAlessandro MassaroMałgorzata SztubeckaAlessandro MeiMałgorzata WernerAlessandro NastroMalik JawarnehAlessandro NocentiniMalinka IvanovaAlessandro PaghiMallikarjuna GowdaAlessandro ParodiMalte FrovelAlessandro PesatoriMalte MisolAlessandro PozzebonMamoon RashidAlessandro PuiattiMamoona HumayunAlessandro RasuloMamoru MimuraAlessandro SapienzaMamoun AbuhelouAlessandro ScanoMamta AgiwalAlessandro ScordoMamun Abu-TairAlessandro SeverinoManabu ItoAlessandro SopegnoManabu SatoAlessandro StefanoManabu ShimakawaAlessandro TerenziManabu TsukadaAlessandro TestaManaf ZghaibehAlessandro TonacciManan ShahAlessandro VitaleManar SamadAlessandro VizzarriMandalika B. SrinivasAlessandro ZonaMandana JalaliAlessia CogatoMandeep Singh Jit SinghAlessia IrreraManes Fernández CabanasAlessia SanninoMan-Fai LeungAlessio BenedettiManfred M. VietenAlessio BruttiMang FengAlessio BuzzinMangali Chinna ChinnaiahAlessio De AngelisManh Le-TranAlessio FascistaManh Pham LinhAlessio MeccaManikandan RamachandranAlessio MondiniManish BhattaraiAlessio TurcoManisha KaushalAlessio ValenteMan-Je KimAlessio VernaManjit KaurAlessio ZulianiManjula RanagalageAlex BystrovManjur KolharAlex JamesManlio BaccoAlex MatlockManlio SantilliAlex MouapiMannix P. BalanayAlex RogersManod WilliamsAlex StellaManoel Firmino De Medeiros JúniorAlexander A. LebedevManohar DasAlexander AgathosManohar Prasad BhandariAlexander ArizaManoj DiwakarAlexander AurellManoj KumarAlexander BelikovManoj PandaAlexander BungeManoj RamanathanAlexander ChizhikMansi GandhiAlexander Christantho BudimanMansour HaddadAlexander DeistingManuel Albornoz-CabelloAlexander DevdarianiManuel ArmadaAlexander DubolazovManuel Casal-GuisandeAlexander E. HramovManuel De La SenAlexander E. MoskalenskyManuel Fernandez-VeigaAlexander FeoktistovManuel Fernández-VeigaAlexander FerreinManuel Filipe P. C. M. CostaAlexander FerwornManuel García SanchezAlexander G. BannovManuel HerreraAlexander GagarinManuel Ibarra-ArenadoAlexander HošovskýManuel Jesús-AzabalAlexander HunoldManuel Joaquim Bastos MarquesAlexander IvannikovManuel Lopez-MartinAlexander KalashnikovManuel MartinezAlexander KöhlerManuel Moreno-EguilazAlexander KölpinManuel Pineda-SanchezAlexander KromkaManuel Prado-VelascoAlexander KrotovManuel Rodríguez-MartínAlexander KukaevManuel Rodríguez-RastreroAlexander LvovskyManuel ServinAlexander MaierManuel Sierra CastañerAlexander MeigalManuel StrianiAlexander MolnarManuel Vilares-FerroAlexander NovitskiyManuela Leticia KimAlexander PogrebnjakManuela TufoAlexander ReshetnyakManuela VagniniAlexander RueschManwinder SinghAlexander S. MachikhinManzoor Ahmed KhanAlexander S. TavlintsevMaode MaAlexander SafonovMaohua LinAlexander SmirnovMaokun LiAlexander SutinMaolin ChenAlexander SutorMaqusood AhamedAlexander TselevMara PistellatoAlexander UzhinskiyMaram Bani YounesAlexander V. GlushkovMarc BrechtAlexander VelichkoMarc CastellaAlexander VinogradovMarc DebliquyAlexander VodyahoMarc FischlerAlexander WolfMarc KurzAlexander ZhbanovMarc ParrillaAlexandra CernianMarc RosalesAlexandra CiorîțăMarc VidalAlexandra FălămașMarc-André DugasAlexandra GoryaevaMarcel KordošAlexandra-Iulia Szedlak-StineanMarcel LeuteneggerAlexandre AndradeMarcel NicolaAlexandre BonattoMarcel WorringAlexandre Cunha BastosMarcella CorduasAlexandre Da Costa SenaMarcella SalvatoreAlexandre Fonseca BrandãoMarcellin AtemkengAlexandre RobichaudMarcello AugelloAlexandre SerresMarcello BertoAlexandrina NanMarcello CasaAlexandros BousdekisMarcello La GuardiaAlexandros PaspatisMarcello ValoriAlexandros SpourniasMarcelo A. C. FernandesAlexandru ApahideanMarcelo Bender PerotoniAlexandru BănicăMarcelo CoertjensAlexandru BarsanMarcelo DavidAlexandru Cosmin ObrejaMarcelo De Carvalho AlvesAlexandru IlieșMarcelo GitiranaAlexandru Isaic-ManiuMarcelo MendozaAlexandru LavricMarcelo PetryAlexandru MartianMarcia I. EndresAlexandru OpreaMarcin AdamczykAlexandru PîrjanMarcin CiecholewskiAlexandru VulpeMarcin DerlatkaAlexei V. MuravyovMarcin GierekAlexei V. PopovMarcin GnybaAlexey A. KytmanovMarcin GodzierzAlexey A. PakhomovMarcin GórskiAlexey BeskopylnyMarcin GrabaAlexey G. VoloboyMarcin HernesAlexey IvutinMarcin JakubowskiAlexey KashevnikMarcin Jan KraśnyAlexey KureevMarcin JasiewiczAlexey KutyrevMarcin KaminskiAlexey PenenkoMarcin KluczykAlexey ShitvovMarcin LawnikAlexey V. AndrianovMarcin LucknerAlexey V. ShvetsovMarcin MaciejewskiAlexey V. VakhinMarcin NiemiecAlexey VereschakaMarcin PeplińskiAlexey Yu BykovskyMarcin ProcekAlexey ZozulyaMarcin RudzkiAlexis MendezMarcin StaniekAlfa SharmaMarcin StrączkiewiczAlfio BattiatoMarcin SuszynskiAlfio Dario GrassoMarcin SzczygiełAlfonso Ariza QuintanaMarcin WoźniakAlfonso Gómez-EspinosaMarcio BasilioAlfonso Infante-MoroMárcio Falcão Santos BarrosoAlfonso Maria PonsiglioneMarcio FialhoAlfonso Martínez-NovaMarco A. Moreno-ArmendárizAlfonso MastropietroMarco AgusAlfonso Penichet-TomásMarco ArnesanoAlfonso Prieto GuerreroMarco BindiAlfonso Ramírez-PedrazaMarco BottaAlfonso RomeroMarco BuzioAlfonso ZambonMarco BuzzelliAlfredo GüemesMarco CamperaAlfredo PinaMarco CarminatiAlher Mauricio HernandezMarco CarnevaleAli Abbasian ArdakaniMarco CiveraAli AbboudMarco Claudio De SimoneAli AbdoMarco D′AlonzoAli AhmadianMarco Dell′IsolaAli AienMarco EscobarAli Akbar SafaviMarco FiorucciAli AlahmerMarco FontanelliAli AlawnehMarco FuringhettiAli AlbarratiMarco GermanottaAli Al-NajiMarco GhislieriAli Al-TemeemyMarco GiannettoAli ArabMarco GirolamiAli BereyhiMarco GiurgiuAli BoolaniMarco GrossiAli Caglar ÖzenMarco LanuzzaAli ChaibakhshMarco LeoAli CinarMarco LolicatoAli Dehghan FiroozabadiMarco LombardiAli Enes DingilMarco Ludovico MarquesAli FarmaniMarco ManciniAli GhaffariMarco MiliucciAli Hassan SodhroMarco NunesAli HayajnehMarco PassafiumeAli I. YurekliMarco PaterniAli JamoosMarco PortelliAli JohariMarco PrandiniAli KaramiMarco RaceAli KhodabandeluMarco RandazzoAli MansoulMarco Raoul MariniAli MansourMarco RighiAli MirzaeiMarco Roberto CavallariAli Mohammad AlqudahMarco ScireaAli Mohammad SaghiriMarco SeraciniAli Moradi AmaniMarco SozziAli Muhamed AliMarco SpadiAli NaumanMarco TambascoAli Nawaz RanjhaMarco TodescatoAli PassianMarco TravagliatiAli RamouzMarco VaccariAli Reza RezvaniMarco VenerandaAli Riza EktiMarco ZannoniAli RohanMarcos Benedito SchimalskiAli Sadeghi HabibabadMarcos BrioschiAli SelamatMarcos CavenaghiAli ShukurMarcos Cesar De OliveiraAli Soltani Sharif AbadiMarcos Dos SantosAli Vatankhah BarenjiMarcos Eduardo ScheicherAli Wagdy MohamedMarcos F. S. TeixeiraAliaa Rehan YoussefMarcos MarcosAliasghar BaziarMarcos MartinsAlice Verticchio VercellinMarcos MontesAlicia Esther AresMarcos Rafael NanniAlicia Martinez RebollarMarcos SchimalskiAlicia Megia-FernandezMarcos Tostado-VélizAlicja BortkiewiczMarcos V. CondeAliihsan SekertekinMarcos Vinícius Da SilvaAlija PašićMarcosiris Amorim de Oliveira PessoaAlimohammad ShirzadifarMarcus SchmidtAlin DinitaMarderos SayeghAlin GramaMare KoitAlin ZamfiroiuMarek BarskiAlina DutaMarek BolanowskiAlina MirzaMarek ČeškovičAlina MomotMarek ChaleckiAlina Pyka-PająkMarek DuricaAlina RoitbergMarek FranaszekAlina-Cristina BuneaMarek GancarzAlinaghi SalariMarek GaworskiAlireza AhmadianMarek JaśkiewiczAlireza Akhavan-SafarMarek JunghansAlireza EnshaeianMarek KęsekAlireza GoliMarek KozieńAlireza ModirMarek KrawczukAlireza SanaeifarMarek KulawiakAlireza SharifiMarek KvetAlisa RudnitskayaMarek LefikAliseri GovardhanMarek MiloszAlison De Oliveira MoraesMarek MoravcikAlison PurcellMarek MrózAliyu AliyuMarek SawerwainAljoša KošakMarek SawickiAlka BishnoiMarek ŠolcAllam Jaya PrakashMarek StanuszekAllan De FreitasMarek StencelAllan L. SchaeferMarek VagašAllison Hess-DunningMargarete AmorimAlmir BadnjevicMargarita AnastassovaAlmir KarabegovicMargarita GamarraAlmira RamanavičienėMargarita Tecpoyotl-TorresAlon DavidyMargaux BouzinAlonso Alonso-AlonsoMargot DeruyckAlparslan SariMargulan IbraimovAlper OzpinarMari NapariAl-Sakib Khan PathanMaria A. ZuluagaAlsath M. Gulam NabiMaria Antonia BrighettiÁlvaro Antón-SanchoMaria Assunta SignoreÁlvaro Astasio PicadoMaría BustosAlvaro González-AngelesMaria ButakovaAlvaro Joffre Uribe QuevedoMaria CappaiAlvaro LopezMaría Carmen PérezÁlvaro Lozano MurciegoMaría Carmen SeijoAlvaro SuarezMaria ChernyaevaÁlvaro SuárezMaria Chiara CascheraAlvin Kai-Xing LeeMaria Claudia SurugiuAlvise SpanòMaria Colomba ComesAlwin PouloseMaria Concetta D′OvidioAmad ZafarMaria Cristina Rodríguez SánchezAmadeo ArgüellesMaria De La Cruz Cardenosa RubioAmadeu GriolMaría de las Nieves González GarcíaAmal HajjajMaría Del Carmen García-RíosAmal KasryMaría Del Pilar Salas-ZárateAmal KhalifaMaria Del Refugio Castañeda-ChavezAmalia Luque-SendraMaria DeRosaAmalia MoutsopoulouMaria DietrichAman BatraMaría Dolores Gómez PulidoAman MahajanMaría Emilia BrassescoAmanda EsquivelMaría Estela Peralta-AlvarezAmandeep KaurMaria Eusebia Guerrero-SanchezAmandeep SinghMaria Fátima PaulinoAmani Yousef OwdaMaria FreitasAmany SarhanMaria G. IoannidesAmar AmouriMaria GamcovaAmar Kumar BarikMaria GhitaAmarajothi DhakshinamoorthyMaria Giovanna BiancoAmber SolangiMaria Giovanna ConfettoAmbrogio P. LonderoMaria Giovanna MasciottaAmeer Hamza KhanMaria Grazia AlaimoAmeersing LuximonMaria Grazia ManeraAmel KsibiMaria GuarneriAmelia Carolina SparavignaMaria I. GoretskaiaAmélia Martins DelgadoMaría Isabel Rodriguez FerradasAmer CharbajiMaría Jesus Gómez GarcíaAmer ZakariaMaria João NunesAmerica CalifanoMaria João RamalhosaAmey KhanolkarMaria José Angélico GonçalvesAmin Beiranvand PourMaria Jose JuradoAmin HekmatmaneshMaria KoroziAmin KhajehdezfulyMaria LeporeAmin NasiriMaria LianouAmin Roshandel KahooMaría LópezAmin ZehtabianMaria Luisa BraungerAmina RhouatiMaría Luisa de la Hoz TorresAmine AbouaomarMaria Luminița ScutaruAmir Akramin ShafieMaría Luz Gámiz-PérezAmir BenzaouiMaria MarinescuAmir GolrooMaria MiritelloAmir HaiderMaria NishevaAmir Hamzeh FarajollahiMaria ParisiAmir Hosein OveisMaría Paz Comech MorenoAmir Masoud MolaeiMaría PedreroAmir Masoud RahmaniMaria PomoniAmir MosaviMaria PrincipeAmir Reza RamtinMaria RajskaAmir SeyyedabbasiMaria RaposoAmir TjollengMaria RashidiAmira ZrelliMaria RigouAmirhamzeh FarajollahiMaria RizziAmirhossein MajidiradMaria RizzoAmirhossein OrumiyeheiMaria Rosa TrovatoAmirmostafa AmirjaniMaria RubegaAmirreza KhodadadianMaria SamarakouAmirul IslamMaria SergeevaAmit ChhabraMaria StrianeseAmit GanatraMaria Teresa InzitariAmit kumar GoyalMaría Teresa Sanz-PascualAmit Kumar SikderMaria Teresa TodaroAmit Kumar TyagiMaria Torres VegaAmit SinghalMaria VasconcelosAmit Vilas SantMaria Vesna NikolicAmitava NagMaria Viorela MunteanAmjad J. HumaidiMaria Vittoria BarbieriAmjad Y. MajidMaria-Alexandra PaunAmlana PandaMariacristina RosciaAmmad MohammadMaria-Dolores CanoAmmar ArmghanMarialisa ScatáAmmar ArshadMariam IbrahimAmmar MuthannaMarian BurduceaAmmar OdehMarian CosteaAmna SaharMarian GogolaAmol D. VibhuteMarian IonAmol S. PatwardhanMarián KučeraAmor ChowdhuryMarián MarčišAmr El-WakeelMarian WnukAmr Mohamed Samy MahdyMarian WysockiAmran BhuiyanMariana Alfaro-GómezAmril NazirMariana BeňováAmrit KaphleMariana Domnica StanciuAmrit MukherjeeMariana DumitrescuAmrita KunduMariana FloriaAmy J. MollMariana GhicaAmy MuellerMariana MarinAmyar AmineMariana PetrovaAn Cong TranMariane R. PetragliaAna BratuMarianna JacynaAna BudimirMarianna KapsetakiAna C. PinhoMarianna NardinoAna Carolina C. De SousaMarianna ObristAna Carolina De Sousa SilvaMarianna ZicharAna Cocho-BermejoMarianne A. AzerAna De-Las-HerasMarianne ComteAna Dinora Guzman-ChavezMaricar AguilosAna DoblasMarieke StammesAna FonsecaMarie-Luce BourguetAna Isabel Calero-CastilloMariem TurkiAna Isabel De Andrés RubioMarietta FodorAna IvaniševićMariia NazarkevychAna Kuzmanic SkelinMarija B. Petrović MihajlovićAna Leticia Fornari CapraraMarija HabijanAna LopesMarija JozanovićAna Luísa RodriguesMarijana DjordjevićAna M. DobrevaMarijke CoetzeeAna M. MadureiraMarijo BuzukAna Manzano-LeónMarilena GiglioAna Maria RochaMarilena IanculescuAna Maria Torres-ArandaMarilena RicciAna Patrícia RochaMarina BastrakovaAna Sanchez-GimenoMarina BertoliniAna Sofia AnicetoMarina ChukalinaAna Sofia Pinho ColimMarina GavrilovaAna SoldadoMarina Gil-CalvoAnabella FerralMarina Ivasic-KosAnaïs BarasinskiMarina ManeaAna-Isabel Corregidor-SánchezMarina MondinAnamaria BjeloperaMarina N. RumyantsevaAnamaria FeierMarina RonzhinaAnand GavaiMarina V. Orda-ZhigulinaAnand NayyarMarina YavtushenkoAnand ShrivastavMarinela MirceaAnanda TiwariMarinko BarukčićAnandram VenkatasubramanianMarino SfornaAnargyros BaklezosMarino VilovićAnas AlazzamMario A. Quiroz-JuárezAnastasia LavrenkoMario A. R. DantasAnastasia VybornovaMario Al SayahAnastasiia KrivoruchkoMario Alberto García RamírezAnastasiia SafonovaMário AntunesAnastasija CollenMario BasletićAnastasija NikiforovaMario CaterinoAnastasios BezerianosMario D′AcuntoAnastasios DimouMario De OliveiraAnastasios DoulamisMario Di MauroAnastasios GiannopoulosMario FargnoliAnastasios KoulaouzidisMario FruschelliAnastasios MpalaskasMario GómezAnastasios ZavosMario GuevaraAnastasiya E. RunnovaMario HirzAnastasiya YurgensonMario InacioAnastassios M. StamatelosMário J. G. C. MendesAnatoliy BatyukMário João S. F. SantosAnatoly SavelievMario LorenzAnatoly SkripalMario Luis Ruz RuizAnbesh JamwalMario MarinelliAnca HanganMario MeroneAnca MaximMario MilerAnca UdristoiuMario MiličevićAn-Chi WeiMario PrsaAncuța ChetrariuMario Rui ArrudaAnda-Elena StanciuMário S. RodriguesAnderson A. FelixMário SilvaAnderson Wedderhoff SpenglerMário VazAndi DirpanMário VéstiasAndjela DraganićMario Wedney de Lima MoreiraAndoni BeriainMarion MundtAndré B. M. SouzaMarios AvgerisAndré BigandMarios G. KrokidisAndré Caceres CarrilhoMarios StogiannosAndré Eugênio LazzarettiMarisela Hernández-GonzálezAndré FurtadoMaristella E. VoutetakiAndre KummerowMarius ArvinteAndre Luiz BelemMarius Iulian MihailescuAndré MermoudMarius MineaAndré Olean-OliveiraMarius PrelipceanuAndré OttoniMarius VolmerAndré Pimenta FreireMariusz BuciakowskiAndre RodackiMariusz ChmielewskiAndré ZúqueteMariusz DejaAndrea BalloMariusz DudziakAndrea BertacchiMariusz GiergielAndrea CataldoMariusz JasinskiAndrea CesariniMariusz JaśniokAndrea CicchettiMariusz JedlińskiAndrea CioncoliniMariusz KoniecznyAndrea CossettiniMariusz KostrzewskiAndrea EhrmannMariusz MrozekAndrea FasanoMariusz PtakAndrea FinMariusz Robert AdamskiAndrea FuscoMariusz SpechtAndrea GenerosiMariusz WęglarskiAndrea GiorgettiMariusz ZiółkoAndrea Gonzalez-MontoroMariya AleksandrovaAndrea LoddoMariya OuaissaAndrea LucchiniMariyam OuaissaAndrea ManniMarjan GolobAndrea MariscottiMarjan MernikAndrea MelisMarjorie DarrahAndrea MichienziMark A. ArnoldAndrea MorabitoMark A. GriffinAndrea MotroniMárk AntalAndrea NettisMark AuslenderAndrea OrsiniMark BradleyAndrea PalumboMark Cronin-GolombAndrea PassarelliMark EverettAndrea PastoreMark FarriesAndrea PiemonteMark HlawitschkaAndrea PirasMark J. PotosnakAndrea PompignaMark James JacksonAndrea RegaMark Kenneth QuinnAndrea RiaMark L. AdamsAndrea RosováMark LevinAndrea SambriMark MelnykowyczAndrea ScorzaMark R. HoffmannAndrea SerafiniMark ShortisAndrea SimonMark T. LemmonAndrea TangherloniMark T. MarshallAndrea TigriniMark WilliamsAndrea TuraMarkiewicz Jakub StefanAndrea VitalettiMarkku TurunenAndrea VitaliMarko BizjakAndreas BirkMarko Č. BarjaktarovićAndreas FischerMarko GulićAndreas HolzingerMarko HorvatAndreas KanavosMarko JermanAndreas KolbMarko KomparaAndreas KönsgenMarko KosAndreas LeichMarko KosticAndreas MøgelmoseMarko MalajnerAndreas PesterMarko MijačAndreas RauhMarko OrosnjakAndreas TsakalofMarko PanicAndreas-Neil UnterreinerMarko PetkovsekAndree Emmanuel WidjajaMarko SaracAndrei A. StolovMarkus EisenbachAndrei C. HolmanMarkus FeldtAndrei DragomirMarkus FiedlerAndrei DumitrescuMarkus HärtelAndrei G. AnisimovMarkus PuchingerAndrei GheorghiuMarkus SchneiderAndrei OlaruMarkus TauberAndrei S. PotapovMaros SmondrkAndrei StanMarouan MizmiziAndrei TarasovMarsel R. VagizovAndrei TsarevMarta BiegańskaAndrei Vasile NastutaMarta FijałkowskaAndrei VatavuMarta Galant-GołębiewskaAndrei VladykoMarta HarničárováAndreia SousaMarta KadelaAndrej BugajevMarta KadłubekAndrej SarjasMarta KarasAndrés Arcia-MoretMarta KopańskaAndres Barajas-SolanoMarta MazurAndres Blanco-OrtegaMarta ParazziniAndres G. MarrugoMarta Parzeniecka-JaworskaAndrés García-FlorianoMarta Rodríguez-HernándezAndres Mendez-VazquezMarta RusnakAndres NistalMarta S. FerreiraAndreu Corominas MurtraMarta SigutAndrew Baxter ReidMarta TessaroloAndrew C. ChienMarta WiśniewskaAndrew ChryslerMartin BeerAndrew GreenhalghMartin BitterAndrew HatchettMartin BoldtAndrew HuntMartin BoltiziarAndrew KwongMartin BrandlAndrew LavenderMartin ČechAndrew LuiMartin ČernýAndrew M. LaneMartin DahmenAndrew PaiceMartin DeckýAndrew R. WillisMartin Enrique Romero-SanchezAndrew RevillMartin FallyAndrew StranieriMartin GoubejAndrew Tanny LiemMartin Hering-BertramAndrew WixtedMartin HofmannAndrey A. RadionovMartin Jonsson-NiedziolkaAndrey A. SamokhvalovMartin KenyeresAndrey A. ZhirnovMartin KöglerAndrey Alexandrovich SidorenkoMartin KopaniAndrey ButovchenkoMartin LavallièreAndrey ChernovMartin MaguireAndrey D. PryamikovMartin MundtAndrey G. AltynnikovMartin RejhonAndrey GalyaevMartin RosarioAndrey Georgievich PaulishMartin SchätzAndrey IvantsovMartin SchmittAndrey KoptyugMartin ŠipošAndrey KurekinMartin SlavikAndrey KuzminMartin ŠtronerAndrey N. KokoulinMartin TreiberAndrey OsipovMartin V. IvanovAndrey SarychevMartin Valtierra-RodriguezAndrey StepnovMartin VašinaAndrey V. SvalovMartin VlkovskýAndrey ZaharievMartin ZeckaAndrija KrtalićMartina CortiAndrius ČeponisMartina De LandroAndrius DzedzickisMartina EspositoAndriy ChmyrovMartina FoschiAndriy SemenovMartina KieningerAndriy SverstyukMartina MammarellaAndriy ZahorulkoMartina Medvidović-KosanovićAndrusca MihaiMartina PastorinoAndry Maykol PintoMartina ŠestakAndry SedelnikovMartino AldrigoAndryushin Konstantin PetrovichMartino GiaquintoAndrzej BiałasMartiwi Diah SetiawatiAndrzej BłażejewskiMárton SzemenyeiAndrzej BuchowiczMarwa MajdiAndrzej BurghardtMaryam Abo-TabikAndrzej DziedzicMaryam Mobed-MiremadiAndrzej FelskiMaryam WeilAndrzej HandkiewiczMarzena Jeżewska-ZychowiczAndrzej KasperskiMarzena KurpinskaAndrzej KatuninMarzena MięsikowskaAndrzej KaźmierczakMarzena PawlikAndrzej KotyraMasaaki BabaAndrzej KrukMasaaki MochimaruAndrzej M. ŚliwczyńskiMasaaki TamagawaAndrzej MileckiMasafumi YanoAndrzej MontwiłłMasaharu KamedaAndrzej MycielskiMasakazu HirotaAndrzej MyśliwiecMasanao NakamuraAndrzej PolanczykMasanori EguchiAndrzej RomanowskiMasashi HamayaAndrzej SikoraMasashi SuganoAndrzej SiomaMasatoshi FutakawaAndrzej SzrombaMasatoshi KozakiAndrzej TomczakMasayuki ShimojoAndrzej UrbaśMasayuki TsunekiAndrzej WilkMasoud BaghelaniAndrzej ZankiewiczMasoud Vesali-NasehAndung Bayu SekaranomMassimiliano BenettiAndy StammMassimiliano CannataAnees AbbasMassimiliano ComissoAneesh ChauhanMassimiliano DonatiAneta Gądek-MoszczakMassimiliano GardaAneta Nowak-MichtaMassimiliano MangoneAneta Ptak-ChmielewskaMassimiliano ZaninAneta SlodekMassimo CacciaAngel Buendía-RomeroMassimo CarossaÁngel Canal-AlonsoMassimo ConfortiÁngel Giménez PastorMassimo DonelliAngel Guillermo BracamonteMassimo GandolaAngela CapocefaloMassimo GaraiAngela ChungMassimo MarielloÁngela María Coves SolerMassimo MerendaAngela StaicuMassimo PiottoAngelo AloisioMassimo SalviAngelo CardellicchioMassimo SorliAngelo LoiMassimo W. RivoltaAngelo Marcelo TussetMassimo ZuccoAngelo PutignanoMassood Tabib AzarAngelo QuarantaMasud AhmedAngelo TroedhanMasudul ImtiazAngga HermawanMatea Zajc PetranovićAngshuman KhanMatej BažecAnh TruongMatej NjegovecAnhtuan LeMatej VargaAniekan Magnus UkpongMateo López-MoralAniel MoraisMateus Coelho SilvaAnik IslamMateus MendesAnil S. BaslamisliMateus Niroh Inoue SanquettaAnindita SarkarMateusz KuklaAnindya NagMateusz PaligaAniruddha ChandraMateusz PasternakAniruddha RayMatheus Silva GonçalvesAnis HamzaMathias BlandeauAnis KoubaaMathias LemmensAnisa KaurMathieu EdelyAnish Prasad ShresthaMatija BušićAnisha PathakMatija MaroltAnita UscilowskaMatin TorabiniaAnith NelleriMatjaz SkrinarAnja SchlichtMatjaž ŠramlAnjan GudigarMats BörjessonAnkit KumarMatteo AneddaAnkit Kumar YadavMatteo Beretta-PiccoliAnkit SharmaMatteo BottinAnkita MisraMatteo Bruno LodiAnkita VaishMatteo BusiAnkur BhattacharjeeMatteo CesariAnmin ChenMatteo CioniAnn L. BaldwinMatteo MelchiorreAnna BoruckaMatteo SensiAnna BurdukMatteo TonezzerAnna Carreras CochMatteo ValtAnna ChiaradonnaMatthew EadyAnna Cleta CroceMatthew FarrowAnna DanihelováMatthew GaddAnna GorbenkoMatthew JacobsAnna Harley-TrochimczykMatthew LeineweberAnna Jakubska-BusseMatthew P. ReedAnna Knitter-PiątkowskaMatthew PikeAnna Kowalewska-KudłaszykMatthew ToewsAnna KubíčkováMatthew WorseyAnna KurzychMatthias GüntherAnna KusiorMatthias Müller-HannemannAnna KusmartsevaMatthias RädleAnna KuwanaMatthias ZeisbergerAnna N. BerlinaMatthieu CaussanelAnna OlczakMattia BadoAnna Paradowska-StolarzMattia BorgarinoAnna Renata DembskaMattia BrambillaAnna SabatiniMattia Francesco BadoAnna StępieńMattia MiottoAnna StrzelewiczMattia UdinaAnna SztyberMaulin JoshiAnna VatsanidouMaura M. ZyllaAnna VisviziMaurice Van KeulenAnna WróblewskaMauricio J. SilvaAnna Zawada-TomkiewiczMauricio LlaverAnnalisa AlloccaMauricio RodriguezAnnalisa BarlaMaurizio ArenaAnnalisa D′ArcoMaurizio MartinaAnnalisa Di BernardinoMaurizio MauriAnnalisa LiccardoMaurizio NaldiAnnalisa PaoloneMaurizio PetrarcaAnnamaria PannielloMaurizio ValleAnnarita D′AddabboMauro BallicchiaAnnarita De MaioMauro BelloneAnne KoelewijnMauro BiffiAnne MichelinMauro CaccavaleAnne PallarèsMauro Callejas CuervoAnne SchwarzMauro D′ArcoAnne Schwarz-PfeifferMauro EpifaniAnne TalneauMauro GattiAnnegret MündermannMauro InnocenteAnnette CaenenMauro TropeaAnnunziata NuscaMauro VenturiniAnnushree BablaniMax M. NorthAnoop Chandrika SreekantanMax Mauro Dias SantosAnoopum S. GuptaMax MenziesAnouar AlamiMax PfefferAnpei YeMax SchleierAnselmo Costa e SilvaMaxim BakaevAnsgar MerothMaxim KalininAnshu Mli GaurMaxim V. RyzhiiAnshuman DasMaxim V. TereshonokAnte PrkićMaxime ElbazAnthony CoustouMaximiliam LuppeAnthony G. ConstantinidesMaximilian SemmlingAnthony J. ClarkMaximo Larry Lopez CaceresAnthony N. SinclairMáximo Méndez BabeyAnthony Neal WatkinsMaximo Morales-CespedesAnthony PamartMay Kristine Jonson CarlonAnthony WakerMaya DimitrovaAntoine NonclercqMayank MishraAntolino GallegoMaycon PeixotoAntolino Gallego MolinaMaysam AbbodAnton AntonovMayue ShiAnton BergantMayur PalAntón García-MartínezMazhar Hussain BalochAnton KonushinMazhar Javed AwanAnton M. ManakhovMaziar YazdaniAnton PletersekMazin MohammedAnton PopovMbarek BacemAnton PyzhevMd Abdus Samad KamalAnton Sergeevich GubankovMd Abdus SubhanAnton VerhoefMd Alamgir KabirAnton VershovskiiMd Asiful IslamAntonella ArenaMd Fahim AnjumAntonella CurulliMd Faruk HossainAntonella FaliniMd Fashiar RahmanAntoni BorrásMd Irfanul Haque SiddiquiAntoni GrauMd Junayed HasanAntonina KalinichenkoMd Liakat AliAntonino ManiaciMd Mamunur RahamanAntonino QuattrocchiMd Manjurul AhsanAntonino S. FiorilloMd Monirul IslamAntonio A. AguiletaMd Mottahir AlamAntónio AbreuMd Nasim KhanAntônio Augusto FröhlichMd Nasim RezaAntonio BartolomeoMd Rasedul IslamAntonio BiondiMd ShafiullahAntonio CardoneMd Shahinur AlamAntónio CardosoMd Sipon MiahAntonio Cartón-LlorenteMd Tanjin AminAntonio ComparettiMd Tanvir HasanAntonio ConcilioMd. Abdul AwalAntonio CostanzoMd. Habibullah BhuyanAntonio de Amescua SecoMd. Hafiz UddinAntonio Del GiudiceMd. Humayun KabirAntonio Di MaioMd. Kamrul HasanAntonio EspositoMd. KowsherAntonio F. DíazMd. Mahidur Rahman SarkerAntonio Fernandez-BalbuenaMd. Noor-A-RahimAntonio Fernandez-LopezMd. Rajibur Rahaman KhanAntonio GarcíaMd. Reazuddin ReponAntonio GenovaMd. Salauddin RaselAntonio GloriaMd. Samsuzzaman SobuzAntonio Hernando GrandeMd. Selim HossainAntonio J. GamezMd. Shafiul AlamAntonio Jesús Yuste DelgadoMd. Shirajum MunirAntonio Jose Ramos SilvaMeei Tyng ChaiAntonio JuniorMeenakshi TripathiAntonio LázaroMegan E. ReissmanAntonio Luca AlfeoMegat Syahirul Amin Megat AliAntonio Luiz Pereira De Siqueira CamposMehdi FoumaniAntónio M. R. C. GriloMehdi GolsorkhtabaramiriAntonio M. RinaldiMehdi HarounabadiAntonio Miguel Ruiz ArmenterosMehdi Hasan ChowdhuryAntónio MonteiroMehdi KhojastehpourAntonio MoschittaMehdi NajibAntonio OliveiraMehdi NeshatAntonio ParzialeMehdi NosratiAntonio Pérez GonzálezMehdi PirahandehAntonio PescapèMehdi RahmatiAntonio PetošićMehdi RakhtalaAntônio PradoMehdi SafariAntonio RodriguesMehedi MasudAntonio RubinoMehmet Akif CifciAntonio Santos-RufoMehmet Akif KoçAntonio Sarasa-CabezueloMehmet Ali KobatAntonio ŠarolićMehmet Alp IlgazAntônio Sérgio Bezerra SombraMehmet Bahri SaydamAntonio SorianoMehmet BayginAntónio SousaMehmet Cüneyd DemirelAntonio Vázquez-LópezMehmet Hakan DemirAntonio ZuorroMehmet Hakan HocaogluAntonios AndreatosMehmet PalanciAntonios KonstantarasMehmet Volkan OzdoganAntonis PeppasMehmet YilmazAntreas KantarosMehmet Zeki KonyarAntti LajunenMehmood NawazAnuar De Jesus Fernandez OlveraMehran GhandehariAnubhav SrivastavaMehran RabaniAnuchit JitpattanakulMehrdad DadgostarAnúp PandíțhMehrdad Shahmohammadi BeniAnup PaulMehrous AliAnupam K. MisraMehtab AlamAnupam KeshariMehtab SinghAnupam Kumar BairagiMei YangAnupkumar BongaleMeimei LaiAnuradha RanasingheMeiqing WuAnurag ChoudharyMeishan ZhaoAnurag JainMeixin ZhuAnurag ThantharateMelania SusiAnuraj SinghMelanie EliasAnusha PenumartiMelchizedek I. AlipioAnusna ChakrabortyMelda ÇarpınlıoğluAnwar Ilmar RamadhanMelike ŞahAnwar KhanMelina P. IoannidouAnwesha KhasnobishMelissa BowenAnwesha MukherjeeMelissa K. LadymanAnyang WangMelkie Getnet TadesseAn-Yeu WuMeng LiuAnzhu GaoMeng LuAo PengMeng MaAo ZhouMeng Ting ChungAomar HadjadjMeng WuAoyong LiMeng YuanAparna AravelliMengce ZhengAparna KumariMengchu ZhouApiradee LimMeng-Fang LinApostolos AxenopoulosMengju LinApostolos D. GalatosMeng-Leong HowApostolos GkamasMenglong LiuApostolos KorlosMengmeng LiuApostolos MelionesMengmi ZhangApostolos ZiakopoulosMengqi YaoAppu Vengattoor RaghuMengqiang ZhaoApu Kumar SahaMengtao SunApurv SaxenaMengwei LiuAqeel AhmadMengyan NieAqil TariqMengyang ZhaoArabas PiotrMengye WangAraceli Ortiz RubioMengyu ChaiAraceli Sanchis de MiguelMeonghun LeeArafa H. AlyMerih PalandokenAraliya MoslehMerlin Simo TagneAranzazu Berbey-AlvarezMesut ŞekerArash AhmadivandMetin AktaşArash HeidariMetin OzkanArash MirhashemiMetin SabuncuArati SridharanMeysam AkbariAravind HarikumarMeysam KeshavarzAravinda C. V. NmamitMhamed SayyouriArbnor PajazitiMia FolkeArcangelo CastiglioneMian Muhammad KamalArchibong Archibong-EsoMiao LiArdashir MohammadzadehMicael Gerardo Bravo-SánchezArdavan RahimianMicaela MorettiniAree AliMicahel HumeAref MeddebMichael A. SteindorferArezoo EmadiMichael AyomohArfan GhaniMichael BermanArfat Ahmad KhanMichael BlankeArghya DattaMichael BolshovArgyro MavrogiorgouMichael ChryssomallisAri VisaMichael DanieleAria Ghora PrabonoMichael DashkovArianit MarajMichael E. GorbunovArianna PesciMichael E. MillerArie M. Van RoonMichael FeldmanArie Wahyu WijayantoMichael FokineAriel DzwonkowskiMichael GarstangAriel Soares TelesMichael HalsteadArie-Willem De LeeuwMichael HauptArif Furkan MendiMichael HoellingArif HussainMichael InggsArif MandanganMichael KeusgenArif Reza AnwaryMichael KomodromosArif UllahMichael KovalevArifur R. KhanMichael L. MyrickAriizumi RyoMichael LipsettArindam GhoshMichael LubaschArindam PaulMichael MangoldAris Cahyadi RisdiantoMichael McGuireAristeidis KarrasMichael MortimerAristidis AnagnostakisMichael MückAritz Bilbao-JayoMichael MunzAritz OzcarizMichael N. KammerArjan GijsenijMichael NewtonArjun ChennuMichael OdellArjun MohanMichael OlsonArjunan GovindarajanMichael P. CravenArkadiusz DobrzyckiMichael ParkerArkadiusz GolaMichael PetersonArkadiusz HulewiczMichael RethfeldtArkadiusz KampczykMichael RothArkadiusz MystkowskiMichael SantiagoArkadiusz SytaMichael SchmidtArkady KaryakinMichael SemenovArkady SerikovMichael SinapiusArletta HawrylakMichael SmallArlindo OliveiraMichael StrohmeierArlindo SilvaMichael TarasovArmah A. de la CruzMichael ThompsonArman Amani BabadiMichael TytgatArmando BarretoMichael WickiArmani BatistaMichael WinokurArmin Dadras EslamlouMichael ZabalaArmin FügenschuhMichaela ServiArmin LehmannMichail J. BeliatisArmin MasoumianMichail MakridisArmindo MeloMichail PatsikasArnab MuhuriMichail ShleymovichArnaldo Guimarães BatistaMichał BoreckiArnaldo Leal-JuniorMichał CiemałaArnau Mir TorresMichal DoligalskiArnaud BreardMichał DudekArnaud BuhotMichał FilipiakArnaud GouelleMichal GorawskiArnaud PeizeratMichał JakubowiczArpan DesaiMichał JasińskiArpan KusariMichał JurekArpan SircarMichal KebisekArputha Vijaya Selvi JamesMichal KelemenArsalan AhmedMichal KlichowskiArsalan GhorbanianMichał KunieckiArsalan OthmanMichał MarcińczukArsalan ShahidMichał NowickiArsénio ReisMichał OstaszewskiArseniy M. BuryakovMichal PodporaArshad FarhadMichal PtacekArtem A. EreminMichal PuškárArtem A. KuznetsovMichał R. NowickiArtem ChizhovMichal R. WrobelArtem E. ShitikovMichal RegulaArtem FeofilovMichal ŠoferArtem M. VesninMichał SpiesznyArtem ShikhovtsevMichał StopelArtem VesninMichal StosiakArthur Henrique de Andrade MelaniMichał StrzeleckiArthur I. PetrovMichal SyfertArthur MolnarMichal VenglárArti Ashish KhapardeMichal VikArtis MednisMichal WieczorowskiArtur AndruszkiewiczMichal WodtkeArtur CichańskiMichalina BłażkiewiczArtur CzerwinskiMichalis VrigkasArtur Jorge FerreiraMichel ArrigoniArtur KaweckiMichel DarmonArtur MakarMichel Eustáquio Dantas ChavesArtur MarciniakMichel GuerrazArtur MikitiukMichel KasserArtur NogaMichel SalomonArtur WilkowskiMichel StefanelloArtūras KilikevičiusMichela PaolucciArturo Forner-CorderoMichelangelo GrossoArturo Gil AparicioMichelangelo GuaitoliniArturo Hernandez AguirreMichele BonninArturo Reyes-GonzálezMichele Di FoggiaArtyom Dmitrievich ObukhovMichele FioreArtyom SelutinMichele GirolamiArumuga PerumalMichele GodioArun KumarMichèle GouiffèsArun Kumar JalanMichele MagnozziArun Kumar SangaiahMichele MastroianniArun Kumar SinghMichele MerliniArun Kumar TripathiMichele PerrelliArun Pandian JMichele RiccioArun Prasanth NagalingamMichelle LiouArun ThirumuruganMichelle MellenthinAruna Kumara RanaweeraMichiya FujikiArunkumar BongaleMieczysław BakułaArunmozhi ManimuthuMieczysław CichońArvid HellmichMiguel Ángel AndrésArvind KumarMiguel Angel CabreraAsaad MigotMiguel Angel Fernandez-GraneroAsad UllahMiguel Ángel García IzquierdoAseem VermaMiguel Angel Guevara LopezAsem M. AliMiguel Angel Luque-NietoAshanthi MaxworthMiguel Angel Maté-GonzálezAshfaqur RehmanMiguel Angel Pardo-VicenteAshiqur RahamanMiguel Ángel Solano-SánchezAshis Gopal BanerjeeMiguel Arjona RamírezAshis TripathyMiguel Bravo-ZanogueraAshish BadiyeMiguel CarrascoAshish BagwariMiguel de Simón-MartínAshish PhophaliaMiguel Díaz-RodríguezAshish SrivastavaMiguel González-RedondoAshkan NejadpakMiguel LorenzoAshley HerdaMiguel Murillo-EscobarAshley LyonsMiguel OrtegaAshok ChhetryMiguel Peixoto De AlmeidaAshok Kumar BaghaMiguel Ponce-VargasAshok PanchapakesanMiguel Poveda-GarcíaAshraf AliMiguel R. MancoAshraf AyoubMiguel Ramón Díaz-Cacho MedinaAshu TanejaMiguel RealpeAshutosh BhardwajMiguel Rodríguez PérezAshutosh SharmaMiguel TorresAshwani KumarMiguel Torres-RuizAshwani Kumar AggarwalMihaela AndreiAshwin AshokMihaela BadeaAshwin Kumar MyakalwarMihaela CirlugeaAshwin Ramesh BabuMihaela HnatiucAsier Salazar-RamirezMihaela Ioana BaritzAsier ZubizarretaMihaela PuiuAsif Ali LaghariMihaela TertisAsif KhanMihaela Tinca UdristioiuAsif NawazMihaela-Ligia UnguresanAsim NiazMihai AnastasescuAsim RayMihai Ciprian MărgărintAsis NasipuriMihai CiucAsisa Kumar PanigrahyMihai GavrilasAsiya KhanMihai NeghinaAskar HamdullaMihai NegruAssef ZareMihai SanduleanuAsterios LeonidisMihai TibuAstghik KuzanyanMihai UdrescuAtanas KurutosMihai ZahariaAtchudan RajiMihaiela IliescuAteeq Ur RehmanMihail Lucian PascuAtef AbdrabouMihailo RisticAtefeh Hajijamali AraniMihalj BakatorAthanasia PapazafiropoulouMihalj PoŝaAthanasios (Thanos) KakarountasMihnea CățeanuAthanasios ChristopoulosMihnea MarinAthanasios FevgasMiho Iryo-AsanoAthanasios G. LazaropoulosMijia YangAthanasios KiourtisMike Oluwatayo OjoAthanasios KoutrasMikel DiezAthanasios LentzasMikhail A. ErshovAthanasios TriantafyllouMikhail BabenkoAthanasios TsipisMikhail BasarabAthanasios VasilakosMikhail BelyakovAthina AlexopoulouMikhail BochkarevAthina TsanousaMikhail E. ProkhorovAtif ButtMikhail G. MozerovAtif MugheesMikhail I. BogachevAtika Rivenq-MenhajMikhail KolevAtilio GameiroMikhail LytaevAtilla KormosMikhail O. EreminAtilla Ozgur CakmakMikhail P. KozochkinAtis ElstsMikhail RodkinAtslands RochaMikhail RonkinAtsushi KuboMikhail SalinAtsushi MaseMikhail ShamoninAttila EgedyMikhail SkripkinAttila FrankoMikhail TarkovAttila GéczyMikhail V. ShestakovAttila GereMikhail Y. PlotnikovAttila HiltMikhail Yu. RyabikinAttila KerteszMikhail ZolotukhinAttila RokaMikolaj KarpinskiAttila TothMikołaj SkowronAttilio FiandrottiMiktha Farid AlkadriAudrey L. KarperienMilad GholamiAudrius ČereškaMilad HeydariaanAugustine O. NwajanaMilad MehriAugusto CiuffolettiMilad Taleby AhvanooeyAugusto MontisciMilan BalazAura MihaiMilan ČabarkapaAurelia Cristina NechiforMilan D. MomčilovićAurelian CraciunescuMilan DadoAurelian MarcuMilan GnjatovićAurelio Hierro RodriguezMilan HofreiterAurélio Luiz Magalhães CoelhoMilan HonnerAurelio MeloMilan MarinkovićAurelio StoppiniMilan PaudelAurora Hernandez-MachadoMilan ProkinAurora MaurizioMilan SedlarAurora SaibeneMilan SmetanaAutilia VitielloMilan TalichAvijit BanerjeeMilan TomaAvinash LakshmikanthanMilena Faria PintoAvtar SinghMileva Samardžić-PetrovićAwadhesh Kumar RaiMilica JankovicAxel BoeseMilica KisićAxel FlinthMilica PetrovićAxel GüntherMilind PadalkarAxel HahnMilka PotrebicAxel SikoraMilon Hossain Aya SaadMilon IslamAyaho MiyamotoMilos B. RadenkovicAyan SealMiloš MilosavljevićAydın BüyüksaraçMiloš MilovančevićAydin EresenMiłosz HuberAyesha MaqboolMiloud SouiyahAyham ZaitounyMilton Carlos Elías EspinosaAyhan BozkurtMilutin M. MilosavljevićAykut KentliMin LiAyman AlneamyMin WangAyman AlthuwaybMin XiongAyman El-ZohairyMin XuAyman Mohamed MostafaMin ZhengAymen AkremiMina HoorfarAymen FlahMina SamukawaAyodeji Olalekan SalauMinas DasygenisAyoub KhanMinchen WeiAytaç AltanMincheol WhangAyush DograMindy LevineAyyaz QureshiMine SeçkinAzadeh NilghazMing Chieh LeeAzat GizatulinMing HuangAzeem HafeezMing LiAzhar Ali ZafarMing LiuAzhar Mohd IbrahimMing ZhangAzhar ZamMing ZhaoAzian Azamimi AbdullahMing-An ChungAzimbek KhudoyberdievMingchung ChouAziz AmineMingda ZhouAziz ShahMing-Der YangAzizollah KhormaliMing-Fong TsaiAzlin AhmadMing-Fu LiAzmi AltintasMinggan LiAznarul IslamMing-Han LiaoAzremi Abdullah Al-HadiMinghang ZhaoB. V. Santhosh KrishnaMinghao LiuBabak EslamiMinghao NieBabak NorooziMinghao ZhaoBabak Shahian JahromiMinghui HongBabar K. RaoMing-Hung HsuBabek ErdebiliMingjian SunBabu R. DawadiMing-Jong TsaiBachirou Guene LougouMinglei ShuBadariah BaisMingliang TaoBader AldawsariMingliang ZhangBadr MochizukiMingliang ZhouBaha ŞenMingqiang YangBahareh ZaghariMingsong WangBahman MoraffahMing-Wei HsuBahrad A. SokhansanjMingwei SunBahram Djafari-RouhaniMingxiao LiBai LiMingxuan ChenBaiquan LiuMingyang GuoBaiyou QiaoMingyu LimBakhtiyar OrazbayevMingyuan ChenBal VirdeeMingyue DingBalasubramaniam StalinMingzhe JiangBalázs BenyóMinh T. NguyenBalázs P. SzabóMinhan LouBalázs VillányiMinho KimBambang KuswandiMinh-Quang TranBan WangMinh-Thuyen ThiBanafsheh RekabdarMinhyeok LeeBander A. AlzahraniMinjoong RimBangjiang LinMin-Kook ChoiBangsheng HanMinkyu SongBanu NergisMinoru OkadaBao JiaMinsu JungBao YangMinsu KimBao Yue ZhangMin-Su Kim (Daelim University College, Republic of Korea)Baoyang LuMin-Su Kim (Wonkwang University, Republic of Korea)Bappaditya MandalMinsub ChungBaptiste CharbonnierMinsuk KooBapun BarikMinvydas RagulskisBaracu Angela MihaelaMinwei ZhangBarbara GiussaniMinxuan ZhouBarbara GuidiMinyi XuBarbara Mavì MasiniMioara PetrusBarbara Nieradko-IwanickaMiodrag ŽivkovićBarbara Ruszkowska-CiastekMiquel Sànchez-MarrèBarbara Sowinska-ŚwierkoszMir Aftab Hussain TalpurBarid Baran LahiriMir Hojjat SeyediBaris Baykant AlagozMir Reza Ghaffari RazinBarjeece BashirMira NaftalyBarry J. McManusMiralda CukaBarry K. LavineMiran PobarBartłomiej AmbrożkiewiczMiran TahaBartłomiej GardaMircea BoșcoianuBartłomiej GuzowskiMircea DulăuBartlomiej IglinskiMircea HuleaBartłomiej ZapotocznyMircea IvanescuBartolomeo MontrucchioMircea NeagoeBartolomeo PantòMircea RadulianBartosz MillerMircea-Bogdan RadacBartosz MindurMirco MuttilloBartosz OlejnikMirco PezzoliBartosz PawłowiczMirela CatalinaBartosz PękosławskiMirela DanubianuBartosz PrzysuchaMirella Gutiérrez-ArzaluzBartosz SzulczyńskiMiren Karmele López De Ipiña PeñaBasaran Bahadir KocerMiriam PekarcikovaBaseem KhanMiriam ZachsenhouseBashar Ali Fraea EsmailMirja AhmmedBasheera MahmmodMirjam Vujadinović MandićBasil Mohammed Al-HadithiMirjana Pejić BachBasilio PueoMirko ViroliBasilio VescioMiron Bartosz KursaBasit QureshiMiron KlosowskiBaskaran InbarajMiroslav BačaBassam MohamedMiroslav BuresBassem MokhtarMiroslav DrljacaBassem ShetaMiroslav JolerBastiaan J. BraamsMiroslav JůzlBastian EngelmannMiroslav KelemenBasuraj BhowmikMiroslav MahdalBayu Adhi TamaMiroslav MinovićBazil Taha AhmedMiroslav NedeljkovićBeat GöpfertMiroslav NovakBeata KurcMiroslav PástorBeata PalczynskaMiroslav PohankaBeata ŚlusarczykMiroslav VarinyBeata SzluzMiroslaw ChmielBeata ZimaMirosław RuckiBeate Oswald-TrantaMirosław SiergiejczykBeatrice CairoMiroslaw SmieszekBeatrice ThielmannMiroslaw SwierczBeatriz GomesMiruna Silvia StanBeatriz Gómez-MartínMisael Lopez-RamirezBebiana CondeMishall Al-ZubaidieBeechu Naresh Kumar ReddyMislav StepinacBehnam Asgari LajayerMitar SimicBehnaz SohaniMitja NemecBehrad KhameseeMitja TruntičBehrouz BehnamMitradip BhattacharjeeBehzad KavianiMitrea Delia-AlexandrinaBehzad V. FarahaniMizuho NishioBehzad VaferiMizuki NakajimaBehzad YektakhahMmeerja Aakhiljabbar JabbarBei SunMoacir Fernandes GodoyBejoy AlduseMoatsum AlawidaBekim FetajiMobayode O. AkinsoluBéla TakaricsMobeen RehmanBelén CurtoMobyen Uddin AhmedBelén Vega-MárquezMoez KrichenBen Abdallah IsmailMofeed Turky RashidBen de Lacy CostelloMohamad AwadBen HamzaMohamad EidBen Van LierMohamad Khairi IshakBen WilligesMohamad ShahabBenchaporn LertanantawongMohamad Zoinol Abidin Bin Abd. AzizBenedetto NastasiMohamed A. AhmedBenedict McLarneyMohamed A. BasyooniBenedikt L. BergmannMohamed Abd ElazizBengü ÖztürkMohamed Abd ElkodousBenhadou SihamMohamed AbdelazeemBenjamas PanomruttanarugMohamed AhmedBenjamin L. SmarrMohamed Ait-El-MokhtarBenjamin MillerMohamed Amine FerragBenjamin RainerMohamed Ben Haj FrejBenoît IgneMohamed BennasarBenoît PiroMohamed BochkatiBenpeng ZhuMohamed BoutaayamouBeomju ShinMohamed DericheBerengère LebentalMohamed ElhabibyBerghout TarekMohamed El-Hadedy AlyBerihu TekluMohamed El-SaadonyBeril SirmacekMohamed ElsheikhBernadette DorizziMohamed Esmail KararBernardo MarquesMohamed EssaBernardo Nugroho YahyaMohamed H. HabaebiBernardo PatellaMohamed H. MussaBernd BaumannMohamed HammadBernd BreidensteinMohamed IbrahemBernd HitzmannMohamed JavadiBernd SauerMohamed M. AwadBernhard KoelmelMohamed M. DarwishBernhard TittmannMohamed M. F. DarwishBerrin YanikogluMohamed MareyBert ArnrichMohamed Meselhy EltoukhyBerthold HornMohamed MosaadBertrand BoudartMohamed MoustafaBertrand DavidMohamed RamdaniBertrand HassaniMohamed S. AbdalzaherBertrand SimonMohamed SalemBessem MkaouerMohamed Sameer AbdallahBettina HeiseMohamed SerhaniBhaben KalitaMohamed TabaaBhabendu Kumar MohantaMohamed TaouzariBhanu Prakash KollaMohamed Wiem MkaouerBharat BhushanMohamed Yaseen JabarullaBharat J. R. SahuMohamed ZaghloulBharatesh ChakravarthiMohamed-Amine OuamriBharath NunnaMohammad (Behdad) JamshidiBharathiraja NaguMohammad A. HasnatBharati NeelamrajuMohammad AbdolrazzaghiBhaswati SenguptaMohammad Abu YousufBhisham SharmaMohammad Afsar UddinBhishma TyagiMohammad AkramiBhupendra KhandelwalMohammad AlibakhshikenariBhupesh MishraMohammad AlmseidinBhuvana KrishnaswamyMohammad AlshayejiBianca MiarkaMohammad Amazad HossainBiao XiangMohammad ArefiBiao YinMohammad Arif Sobhan BhuiyanBiao YuMohammad Asif ZamanBih-Chyun YehMohammad BelalBihter ErolMohammad DaoudBijay Kumar RoutMohammad Ebrahim MohammadiBijun LiMohammad Faiz Liew AbdullahBikram KoiralaMohammad Farooq WaniBilal AlatasMohammad G. H. AlijaniBilal ManzoorMohammad H. HasanBilim Atli-VeltinMohammad H. Nadimi-ShahrakiBill MoranMohammad Hashemi-TabatabaeiBillur BarshanMohammad HeydariBin ChenMohammad Hossain HeydariBin GaoMohammad Iman Mokhlespour EsfahaniBin LiuMohammad JafariBin LuMohammad Junaid KhanBin TangMohammad Khalily-DermanyBin WuMohammad KhisheBin YangMohammad Masih SediqiBinbin JiaoMohammad Masum BillahBinbin YingMohammad MoghimiBindeshwar SinghMohammad MomenyBing JiMohammad Nishat AkhtarBing XuMohammad Nurunnabi MollahBing YuMohammad Rashed Iqbal FaruqueBing YuanMohammad RashidBing ZhouMohammad RazzaqueBingbing GaoMohammad Reza Chalak QazaniBingfei FanMohammad Reza KarafiBingfeng TanMohammad Reza MahmoudiBinghan XueMohammad Reza RezaeiBinghao WangMohammad S. ShourijehBingjie DangMohammad SajidBingliang HuMohammad ShahbakhtiBingpu ZhouMohammad Shawkat HossainBingshan HuMohammad ShojafarBingsheng LiMohammad ShokouhifarBingxi XiangMohammad Shokrolah ShiraziBingxi YanMohammad SoleymaniBingzhao LiMohammad Tariqul IslamBinh P. NguyenMohammad TauviqirrahmanBinhai ZhuMohammad Tavakkoli YarakiBiplav ChoudhuryMohammad YekrangniaBirgitta Dresp-LangleyMohammad ZamanBishoy SedhomMohammad ZarourBiswajeet MukherjeeMohammadhassan ValadanBiswajit MaharathiMohammadjavad HamidiaBjörn KrügerMohammadmehdi SaberioonBlake M. AllanMohammadreza ForouzeshfardBo ChenMohammadreza IlkhaniBo ChengMohammadreza Reza MalekBo DaiMohammed A. A. Al-qanessBo FuMohammed A. FayadBo GaoMohammed AbdulridhaBo HuangMohammed Alaa HamidBo LiMohammed AlghamdiBo LiuMohammed AlharbiBo MaoMohammed Ali JallalBo PengMohammed Al-SaremBo TanMohammed Amine MerzougBo WangMohammed ArefBo YangMohammed BazBo YiMohammed DiykhBo ZhangMohammed El Amine SlamaBo ZhengMohammed El-AbsiBo ZhouMohammed El-DiastyBoaventura Freire Dos ReisMohammed GismallaBoaz Ben-MosheMohammed H. AlsharifBob FriedmanMohammed HadwanBob ZhangMohammed HajjajBoby GeorgeMohammed HamzaBodor MariusMohammed HussainBofan SongMohammed Ibrahim SayyedBogdan AntonescuMohammed IsamBogdan BițăMohammed Ismail Russtam SuhrabBogdan C. FloreaMohammed KoubitiBogdan C. RaducanuMohammed M. AlaniBogdan Constantin NeaguMohammed M. FaragBogdan DumitrascuMohammed MecheeBogdan DziadakMohammed RasheedBogdan GhermanMohammed Riyaz AhmedBogdan GilevMohammed Saber EliwaBogdan GrozaMohammed Salah Al-RadhiBogdan IancuMohammed Saleh Ali MuthannaBogdan IliescuMohammed SalemBogdan LipušMohammed Shamim KaiserBogdan MiedzińskiMohammed TariqueBogdan MocanMohammed ZakariahBogdan PankiewiczMohammedhusen ManekiyaBogdan PavkovicMohan KumarBoguslaw ButryloMohand DjeziriBohao FengMohanraj ThangamuthuBohdan RusynMohd Abdul AhadBohong WangMohd Anuaruddin Bin AhmadonBoitumelo J. MatsosoMohd Anul HaqBojan ZajecMohd Ashraf AhmadBokhimi XimMohd Azizi Bin Abdul RahmanBokyu KwonMohd Dilshad AnsariBolelang H. SibollaMohd Farhanulhakim Mohd Razip WeeBolem Sai ChandanaMohd Hafiz Fazalul RahimanBolin CaiMohd Halim Mohd NoorBo-Lin JianMohd Heikal HusinBongsoon KangMohd Nadhir Ab WahabBoniphace KutelaMohd Saiful Azimi Bin MahmudBoon Giin LeeMohd Shahir Bin KasimBoon Hee SoongMohd Shahrimie Mohd AsaariBoonserm KaewkamnerdpongMohd ShuaibBoran ŞekeroǧluMohd TariqBoris G. GorshkovMohd TausifBoris GinzburgMohd Zamri IbrahimBoris Igor PalellaMohd. SaifuzzamanBoris KirovMohd. Zulfaezal Che AzeminBoris KovalerchukMohit BajajBoris KrylovMohsen AmjadianBoris Mikhailovich ShumilovMohsen AnnabestaniBorislav SavkovicMohsen KoohestaniBorislav StoyanovMohsen NiazianBorja BordelMohsen OmidvarBor-Jiunn WenMohsen Yoosefzadeh NajafabadiBorko Đ. BulajićMohsin RazaBorong HuMohtadin HashemiBor-Ran LiMoisés Jiménez MartínezBor-Shing LinMojmir KollárBorys FrankewyczMojtaba AsadiBosko NikolicMojtaba Dehghan-ManshadiBossoufi BadreMojtaba GhodsiBostjan BatageljMojtaba Jafari TadiBoštjan ŠumakMojtaba JoodakiBotao JiangMojtaba KavianiBotond Sandor KireiMojtaba KhanesarBouabid El OuahidiMojtaba YariBoulouird MohamedMolina-Granja FernandoBouraoui IlahiMominul AhsanBowei ChenMona JaberBoxuan ZhongMonia HamdiBoyu KuangMónica Ballesta GaldeanoBoyuan LiMonica DinuBożena KostekMonica FigueiredoBozena Kosztyla-HojnaMonica FiraBozhao QiMonica FlorescuBožidar PotočnikMónica Lorena Sánchez LimónBrach PostonMónica MartinsBradley J. EdelmanMonica PătrașcuBradley J. RothMonica SirouxBragadeshwaran AshokMonica TiboniBrahim AïssaMonika BłaszczyszynBrahim BrahmiMonika BleszynskiBrana JelenkovicMonika KatariaBrandon RussellMonika KwokaBranislav ŠarkanMonika PartykaBranka JokanovicMonika PetelczycBranko BabusiakMonika StomaBranko KolaricMonika Zawadka-KunikowskaBranko ŠterMontañes Rosa RodriguezBratislav MarinkovićMoonsik MinBrenda DaviesMorad ElshehabiBrenno MenezesMoreno MarcelliniBrett BorghettiMorgan MadecBrian FildesMoritz SchumannBrian G. BoothMorozov EugeneBrian HooverMortaza Saeidi-JavashBrian K. FoutchMosabber Uddin AhmedBrian KelleyMoses EkpenyongBrian M. BeckMoshe AverbukhBrian VestalMoshe KamBrian WellsMoshi GesoBrian WillisMoslem DehghaniBrigida AlfanoMostafa AzimzadehBrígida Mónica FariaMostafa ChowdhuryBrij B. GuptaMostafa HefnawiBrij GuptaMostafa MasudBriliant A. PrabowoMostafa MjahedBrojeshwar BhowmickMostafa TaghizadehBruce BernackiMostafa Yuness Abdelfatah MostafaBruce GnadeMostefa Mohamed-SeghirBruce Jeffrey RogersMoti ZwillingBruce MehlerMotoya SuzukiBruce WallaceMouath ObaidatBruno Albuquerque De CastroMouquan ShenBruno BrunoneMourad KchaouBruno Da SilvaMourad KchouBruno DamasMourad Khayati Mourad KhayatiBruno DiasMourad LahdirBruno FernandesMourade AzrourBruno FiondaMoustafa HusseinBruno GoncalvesMoustafa NasrallaBruno José Nievas SorianoMrinal Kanti BhowmikBruno MasieroMu Ku ChenBruno MezêncioMuahammad IslamBruno Miguel FerreiraMuahmmad Yeasir ArafatBruno Nazario CoelhoMuammar SadrawiBruno PiccirilloMubashir HussainBruno Sanches De LimaMu-Chun WangBruno SousaMucong LiBruno Tardiole KuehneMuddasar NaeemBruno WacogneMueen UddinBruno ZoricMuftah FraiferBryan ScotneyMugahed A. Al-antariBryan WongMugahed Al-antariBudaszewski DanielMugdha PadalkarBuddhadev PurohitMughal Muhammad FarhanBuddhika MadurapperumaMuguras MocofanBugra AlkanMuhamad Risqi Utama SaputraBuket D. BarkanaMuhammad A. RahmanBülent DelibasMuhammad AamirBumshik LeeMuhammad Aamir AliBurak AlakentMuhammad Abdul MunnafBurak GerisliogluMuhammad Abrar AkramBurcu Arman KuzubasogluMuhammad AdilBurhan Rashid HusseinMuhammad Adnan KhanBurkhant SuerfuMuhammad Ahsan GullBurkhard CorvesMuhammad Ahsan SaeedBurkhard WrengerMuhammad AjazByeng Dong YounMuhammad Akmal RemliByeong Ha LeeMuhammad AliByeongjoon NohMuhammad Ali ButtByeongseon JeongMuhammad Ali ImranByoung Chul KoMuhammad Ali JamshedByoung-Hee LeeMuhammad Ali QureshiBystrík DolníkMuhammad Aminur RahamanByung Tae OhMuhammad Amir KhanByung-Gon ChaeMuhammad AnshariByung-Gyu KimMuhammad Arif MahmoodByunghun LeeMuhammad ArsalanByungki KimMuhammad ArshadByung-Kuk SeoMuhammad Asghar KhanByungwoon ParkMuhammad AsimCadmus YuanMuhammad AslamCaesar WuMuhammad Attique KhanCaetano Mazzoni RanieriMuhammad Awais JavedCaglar BayikMuhammad AzizCai LuoMuhammad AzmatCai TaoMuhammad BabarCairo L. NascimentoMuhammad BilalCalasan MartinMuhammad Faisal NadeemCaleb RasconMuhammad Firdaus Akbar Jalaludin KhanCalin CorciovaMuhammad Gufran KhanCalin RusuMuhammad Hassan KhanCalin VaidaMuhammad HussainCălin VlădeanuMuhammad IbrahimCalin-Adrian ComesMuhammad IbrarCalogero SchillaciMuhammad Iftikhar HussainCamelia DelceaMuhammad IkramCamelia LemnaruMuhammad IrshadCamelia M. PinteaMuhammad Jawad MirzaCamil LanceaMuhammad Jehanzeb Masud CheemaCamila Paes SalomonMuhammad KamranCamilla CattaneoMuhammad KashifCamilla KärnfeltMuhammad Kashif ShahidCamilla ScapicchioMuhammad Khalil AfzalCamilo DíazMuhammad KhanCan DongMuhammad Mostafa MonowarCan HuangMuhammad Mubasher SaleemCan JiangMuhammad Muqeet RehmanCan KoyuncuMuhammad Nurul IslamCan Trong NguyenMuhammad RashidCandan GokceogluMuhammad Rawi Mohamed Mohamed ZinCàndid ReigMuhammad Reza Kahar AzizCândido DuarteMuhammad Saif Ullah KhalidCanek PortilloMuhammad SajidCaner SahinMuhammad SaleemCapace AlessiaMuhammad Salman Al FarisiCarina Pinheiro-AraujoMuhammad SardarazCarl AnthonyMuhammad ShafiqCarl James DebonoMuhammad ShahidCarl MalingsMuhammad Shahid JabbarCarla QueirosMuhammad Shahwaiz AfaquiCarles GomezMuhammad Shahzad SarfrazCarlo Alberto ArtusiMuhammad Sheikh SadiCarlo BongioanniMuhammad Shemyal NisarCarlo De BenedictisMuhammad Shuja KhanCarlo MolardiMuhammad SohailCarlo NavarraMuhammad SulaimanCarlo RainieriMuhammad Taha JilaniCarlo RicciardiMuhammad TahirCarlo SabbareseMuhammad Tariq MahmoodCarlos A. JaraMuhammad Umar AftabCarlos A. P. SoaresMuhammad Umar KhanCarlos AbreuMuhammad Umer FarooqCarlos Aguilar-IbañezMuhammad UsmanCarlos Alberto Romero-PiedrahitaMuhammad Usman Ali KhanCarlos Alejandro Granados-EchegoyenMuhammad Usman HadiCarlos AndujarMuhammad Wajid UllahCarlos BlanesMuhammad WaqasCarlos BoyaMuhammad Waqas NadeemCarlos CaroMuhammad Waseem AshrafCarlos CarvalhoMuhammad Yousuf Irfan ZiaCarlos Couder-CastañedaMuhammad ZahidCarlos Curvelo SantanaMuhammad Zahid KhanCarlos David ZuluagaMuhammad ZiaCarlos Díaz-CastilloMuhammad Ziaul HoqueCarlos Doñate BuendiaMuhammad ZubairCarlos EscobedoMuhammad Zubair AsgharCarlos Felipe Rengifo RodasMuhammed Enes AtikCarlos FrajucaMuhammed YildirimCarlos Francisco Simões GomesMuhammet Fatih AslanCarlos G. BerrocalMuhammet Fikret ErcanCarlos G. JuanMuharrem KaraaslanCarlos Galván-TejadaMuh-Tian ShiueCarlos García-RubioMujib L. PalashCarlos GriloMukesh Kumar (CSIR, India)Carlos H. LauroMukesh Kumar (Lovely Professional University, India)Carlos HernandezMukesh Kumar ThakurCarlos Javier Sosa GonzálezMukherjee SaswataCarlos L. Garrido AlzarMukhriddin MukhiddinovCarlos López-de-CelisMukundam VangaCarlos M. MateoMunam Ali ShahCarlos Manuel G. S. LimaMunawir MunawirCarlos MarquesMuneesh MaheshwariCarlos PascalMunesh SinghCarlos Pérez-LópezMurad A. RassamCarlos Perez-RamirezMurad KhanCarlos Perez-VidalMuralikannan MaruthamuthuCarlos PlateroMurat BasaranCarlos R. Del-BlancoMurat DemiralCarlos R. MinussiMurat Mustafa SavrunCarlos ReusserMurat ÖztürkCarlos Roberto Hall BarbosaMurat SimsekCarlos Roberto MinussiMurat Tolga ÖzkanCarlos Roldán-BlayMurillo Ferreira Dos SantosCarlos RosadoMurilo Sérgio JuliãoCarlos Sánchez MuñozMurthy ChavaliCarlos Soubervielle-MontalvoMurugan VeerapandianCarlos Torres-TorresMurugappan MurugappanCarlos TrenadoMusa NdiayeCarlos TrujilloMusa YilmazCarlos Valderrama SakuyamaMusab HameedCarlos VillaseñorMusheer AhmadCarlos ZafraMusrrat AliCarlos Zuluaga-DomínguezMustafa Berkan BicerCarlos-D. Martínez HinarejosMustafa Erden YildizdagCarly DaleyMustafa Hikmet Bilgehan UcarCarmelo GarcíaMustafa KuntoğluCarmelo MilitelloMustafa NamdarCarmelo PirriMustafa UstunerCarmelo ScuroMustafa YılmazCarmen BisogniMustaqeem KhanCarmen DebeleacMuthaiah ShellaiahCarmen DelgadoMuwei JianCarmen JarenMuzaffar RaoCarmen María Calama-GonzálezMuzaffer Can IbanCarmen MihailescuMyeounggon LeeCarmen Moret-TatayMyleswamy AnandkumarCarmen Peláez-MorenoMyounggon KangCarmine CiofiMyron G. BestCarol HargreavesMyungkook MoonCarola Andrea Figueroa FloresN. N. SrinidhiCarolin KolmederNabarun BhattacharyyaCarolina Del Valle SotoNabarun SahaCaroline HartleyNabaz R. KhwarahmCasimer DeCusatisNabil Abdel JabbarCasper SchouNabil Ben NessibCatalin AlexandruNacer FarajzadehCatalin BojaNader ShehataCatalin Daniel GalatanuNadhir HammamiCatalin DumitrescuNadia BeluCatalin I. PruncuNadia BrancatiCatalin PopescuNadia NedjahCatalin StoeanNadine ChapmanCatalina IticescuNadir ShahCătălina Iulia SăveanuNaeem JanCatalina-Alice BrandusNafees MansoorCatalin-Ionut SilvestruNagaraj P. ShettiCaterina CiminelliNagaraj YamanakkanavarCaterina CrediNagham SaeedCaterina PiazzaNaghmeh MahmoodianCatherine RousseyNagur Shareef ShaikCátia LeitãoNahian RahmanCecilia Di RubertoNahid Al MasoodCecilia GarciaNail AkarCecilia Jimenez-JorqueraNajam HasanCecilia SuraceNajlae IdrissiCédric Stéphane Tékouabou KoumétioNajme MansouriCees A. SwenneNajmeh SamadianiCelal ErbayNakul PandeCeleste Campo VázquezNalla AnandakumarCelia López-OngilNam KimCelso A. R. L. BrennandNam Tuan LeCelstine IwendiNam-Gyoon KimCem DirekoğluNam-Su JhoCemil ColakNam-Yong LeeCen WuNan GuoCenk ÇalışkanNan WuCésar A. CollazosNan ZengCesar CameriniNan ZhangCesar Da CostaNan ZhaoCésar Martínez-OlveraNan ZhuCesar San Martin SalasNandakishor YadavCésar TeixeiraNandakumar NambathCesar TiradoNan-Fu ChiuCesare CamettiNansong WuCesare GrazioliNaoki KanoCezary SpechtNaoki MoriCezary SzczepanskiNaoki OhshimaChaiyaporn SilawatchananaiNaoki ShinoaraChakfong CheangNaomitsu UrasakiChamara V. SenaratnaNaoshi KanekoChamindu DeepagodaNaoto YagiChan Hee ParkNaoya KasaiChan Hwang SeeNaoyuki TakeuchiChandan PandeyNarendra KhatriChandani SenNaresh KotadiyaChandra Prakash SinghNaresh Kumar DarimireddyChandra Segar ThirumalaiNasem BaderldinChandra Sekhar PappuNaser GolsanamiChandrasekaran JayaramanNaser Ojaroudi ParchinChandrasekaran SelvamNasim HajariChang Ho KangNasim RashidiradChang LiuNasima BegumChang WangNasire UlucChang Won LeeNasro Min-AllahChang XiaojunNasrullah KhanChang XuNasser Abu ZeidChangcai YangNasser YimenChangcheng ShiNatalia DistefanoChanghai LiuNatalia Garrido-VillénChang-Hee LeeNatalia GrzebiszChangho SongNatalia ParoulChang-Hua LienNatalia PorotnikovaChanghui JiangNatalie BaddourChangjin XuNataliia MelnykovaChangli LiNataliya PankratovaChang-Sei KimNataliya ShaburovaChangsen SunNatalya KondratyevaChang-Sheng LinNatan T. ShakedChangsoon ChoiNatanael Cuando-EspitiaChang-Wei HuangNatarajan RavikumarChangwon KimNataša RančićChangwoo LeeNataša TošanovićChangxin GaoNatasha VukovicChangyoon JeongNathan EddingsaasChangyou LiNathan MakowskiChangyu LiuNathan SwamiChang-Yue ChiangNathaniel T. BerryChang-Yull LeeNatheer KhasawnehChan-Jung KimNatividad Duro CarraleroChanseok JeongNatthapol SaovanaChansoo KimNavdeep DahiyaChanyoung JuNaveed Ahmed AzamChao BiNaveed EjazChao ChenNaveed Ishtiaq ChaudharyChao GuNaveed KhanChao HuangNaveed Khan BalochChao LiuNaveed Ul HassanChao LuNaveen ChauhanChao MeiNaveen KumarChao TanNaveen Kumar NishchalChao WangNaveen MahantiChao WuNaveen R. VenkatesanChao XuNavid BayatiChao YangNavid P. GandjiChao ZhaiNavid ParsaChaochen GuNavid Rahbari AsrChao-Chung PengNayan Ranjan SinghaChaofeng YeNayden NenkovChao-Fu WangNazakat AliChao-Hsien LeeNazario FoschiChao-Hui YehNazeer MuhammadChaokuei LeeNazia HameedChao-Lin KuoNazrul Anuar NayanChaomin ZhangNeal WagnerChao-Ming WangNebojsa BojovicChao-Tung YangNecmettin Firat ÖzkanChaouki HannachiNedim TutkunChaowei SiNeela SatheeshChaoxia ShiNeelakandan SubramaniChaoyang GongNeelamadhab PadhyChaoyang TiNeelesh KumarChaoyi ShiNeema MdumaCharalampos GeorgiadisNeethu SreenivasanCharalampos N. PitasNegar FarzanehCharalampos OrfanidisNeil GlossopCharis LiapiNeil Yuwen YenCharitha DiasNejc KozarCharles CarlsonNejc ŠarabonCharles F. BabbsNektarios KalyvasCharles IkerionwuNektarios MoraitisCharles J. StuhlNemai Chandra KarmakarCharles JoubertNemanja MačekCharles WalterNemitari AjienkaCharlie M. WaughNenad D. FilipovićChase Qishi WuNenad KoropanovskiChastine FatichahNenad MedićChatchawal WongchoosukNenad NenadicChathura AbeywickramaNerija ŽurauskienėChathuranga KumarageNeringa DubauskieneChau Nguyen DinhNéstor Pérez-ArancibiaChe-Cheng ChangNeven UkrainczykChe-Chih TsaoNevin DarwishChee Keong TanNewcomb RobertChee Meng Benjamin HoNezah BalalChee-Onn ChowNghia HuynhChein-I ChangNgoc Dung BuiChen CaoNgoc Q. LyChen Chin-LingNgoc Thang BuiChen LiNgombo ArmandoChen ShenNguyen Thi Hoang OanhChen YangNguyen Truong ThinhChen ZhangNguyen Vinh KhuongChen ZhuNguyen Xuan-MungChenchu XuNhat-Duc HoangCheng Ee MengNhu-Tai DoCheng JiangNiall O′ MahonyCheng JieNiamat HussainCheng LiuNiannian WangCheng ShenNicholas A. FriedenbergCheng ShiNicholas A. GiudiceCheng Shian LinNicholas CaporussoCheng Siong ChinNicholas E. ProtonotariosCheng SongNicholas F. MatererCheng SunNicholas GiudiceCheng WangNicholas HallforsCheng YanNicholas PolydoridesCheng ZongNicholas SamarasCheng-Chen ChenNicholas TothillCheng-Chi LeeNicholas WiseCheng-Chih HsuNick Andrei IvǎnescuCheng-Chung ChangNick DonaldsonChenggeng HuangNick HarrisCheng-Hsin ChuangNick VordosCheng-Hsiung LeeNico SuranthaCheng-Huan ChenNicola AnselmiCheng-Hung LinNicola Di StefanoChengjiang ZhouNicola GarzanitiChengjin LyuNicola LiberatoreCheng-Ju YuNicola LopomoChengjun HuangNicola MontemurroChengkuo LeeNicola OrzalesiCheng-Liang WangNicola PergolaChengliang ZhangNicola PetroneChenglizhao ChenNicola SeccianiChenglong HuangNicola ZeniChenglong LiNicolae FilipChenglong ShaoNicolae PopChenglong YouNicolae Popescu-BodorinChengming JiangNicolae TudoroiuChengming LuoNicolai SpicherChengning WangNicolas BinaudCheng-Shu YouNicolas KuhnChengwu ZhangNicolás Laguarda-MiróChengyi WangNicolas Le SommerCheng-Yuan ChangNicolas LoriChen-Hua FuNicolas RagotChenhui QianNicolas SegalChenn-Jung HuangNicolas SpinelliChen-Wen YenNicolas TsiftesChenxi YangNicolas TurpinChenyao YangNicole McFarlaneCheol-Soo ParkNicoleta DospinescuCherie S. TanNicoleta EnescuCherng-Yuan LinNicoletta NicolaouChester S. J. HuangNicolina SciaraffaChethan K. N. Nicolò DecarliChetna GuptaNicolò LagoChe-Tsung LinNicos MaglaverasChe-Yu LinNicu BizonChhaya GroverNida AslamChi Bum AhnNiels NijdamChi Cuong VuNihan AydemirChi WuNika PavlovićChi XuNikhel GuptaChi ZhangNikita GabdullinChia Hung KaoNikita MartyushevChia-Chen ChangNikita StsepuroChia-Chi ChengNikitas NikitakosChia-Hong YengNiklas HoppeChia-Hsun ChangNikola MirkovChia-Hui LiuNikola SakačChia-Hui WangNikola ShakevChia-Hung ChangNikola TomicChia-Jung ChangNikola ZaricChiaki RaimaNikola ZogovićChia-Liang LuNikolai A. SidorovChia-Lun LoNikolai S. PerovChia-Ming ChangNikolai SimonovChia-Ming YangNikolaos A. ChrysanthakopoulosChiao-Chi LinNikolaos BarasChiara BedonNikolaos D. KouvakasChiara CeccariniNikolaos DimitriouChiara CosenzaNikolaos KantartzisChiara GaldiNikolaos KarathanasopoulosChiara IacovelliNikolaos L. TsakiridisChiara LapucciNikolaos ManousakisChiara RomanengoNikolaos MitianoudisChiara SaraciniNikolaos NikitasChiara SchiattarellaNikolaos StefanakisChiara ZuccoNikolaos VidakisChia-Yen John LeeNikolay A. KorolevChia-Yen YangNikolay KakanakovChia-Yi HuangNikolay KiktevChi-Ching ChangNikolay KlenovChichung CheungNikolay L. KazanskiyChieh-Li ChenNikolay MadzharovChien-Chang ChenNikolay MukhinChien-Chang HsuNikolay ShilovChien-Cheng JungNikolay Todorov AtanasovChien-Chou LinNikolina FridChien-Kuo HsiehNikolina JankovicChien-Sheng LiuNikos D. FakotakisChien-Wen HsiehNikos KalatzisChih Chiang HongNikos PetrellisChih Jer LinNikos TheodoulidisChih-Chang YuNikunj A. BhagatChih-Chen YihNilanjan ChattarajChih-Cheng HuangNilanjan DeyChih-Chin YangNilesh GoelChih-Chun HsiehNileshi SarafChih-Hao LeeNils Tångefjord BasseChih-Hong LinNima Jafari NavimipourChih-Hsien ChengNima JavanbakhtChih-Hsien HsiaNimsiri AbhayasingheChihkun KeNina CurcicChih-Peng FanNina VerdelChih-Wei TangNing GaoChihwen SuNing LiChih-Wen WuNing LiuChih-Yu HsuNing SuChih-Yu WenNing WangChihyung WenNing XiongChii Bin WuNingqi LuoChikahiro ImashiroNingyuan GuoChi-Ken LuNino GrizzutiChilai ChenNinoslav MajurecChin Poo LeeNir RegevChin Wei LaiNir ShvalbChin-Chun TsaiNiranjana SampathilaChing-Chi HsuNirav JoshiChing-Chich LeuNirmal MazumderChing-Chung YinNirmala KandadaiChing-Fu WangNirvana PopescuChing-Hua TingNishant KumarChing-Hung ChangNishant TripathiChing-Hung LeeNishu GuptaChing-Hwa ChengNitesh FundeChing-Lung ChangNitin GoyalChing-Seh WuNitin GuptaChing-Sheng LinNitin SinghChing-Ta LuNitish ThakorChing-Te KuoNivethitha SomuChing-Wei WangNivin JoyChinmay ChakrabortyNiyaz Mohammad MahmoodiChinmaya Kumar NayakNizamud DinChinmaya PanigrahyNizar Faisal AlkayemChinmoy Kumar PanigrahiNizar JaberChinnasamy PonnusamyNobukazu TeranishiChinnasamy RajendranNobuyasu NaruseChin-Shiuh ShiehNoé Pérez-HiguerasChintan BhattNoela Sánchez-CarneroChinthaka PremachandraNoemí de-los-Santos-ÁlvarezChiranji Lal ChowdharyNoemí PérezChiranjibi SitaulaNogami HirofumiChittaranjan PradhanNoman KhanChitti Venkata KrishnamurthyNoor Saeed Khan KhattakChi-Wen LungNoor ZamanChi-Yi TsaiNopphon KeerativorananChi-Ying LinNora CastnerChi-Yu HsuNorbert BaderChi-Yuan ChangNorbert JungCho Nilar PhyoNorbert PalkaChokri SendiNorbert Scherer NegenbornChong ChenNorbert SchmitzChong LiNorbert TarjányiChong LiuNorbert WehnChong WangNorhanani Abd RahmanChong Yang ChuahNorhashila HashimChong ZuNorifumi KawabataChonghang ZhaoNorihiro InagakiChongjing CaoNorio MiyoshiChongsheng ChengNorio TagawaChongyu ZhuNoritaka YusaChoon LeeNorman R. WilliamsChoonhwa LeeNorshah Afizi ShuaibChouikhi SamiraNorsinnira Zainul AzlanChow Yin LaiNoshin FatimaChow-Shing ShinNouf AlzahraniChris HallNouha AlcheikhChris KarayannisNourdine AlianeChris PerrellaNoureddine LakouariChrister JohanssonNuno BastosChristian Bräuer-BurchardtNuno CrespoChristian Campos-JaraNuno CruzChristian Cuadrado-LabordeNuno Hélder SilvaChristian DanielNuno M. GarciaChristian Di NataliNuno Miguel Fonseca FerreiraChristian EspositoNuno Pessanha SantosChristian Fernández-CampusanoNuria Novas CastellanoChristian GaleaNuria PardoChristian GreveNúria Parés MarinéChristian KlauerNursuriati JamilChristian KrambergerNurul I. SarkarChristian MüllerNurul Izza Mohd NorChristian NapoliNuruol MohdChristian RaffelsbergerNuvia M. SaucedoChristian RoedelNwabueze EmekwuruChristian TanislavNyothiri AungChristian VeltmannOana GemanChristian VogeleyObinna AnejionuChristian WernerOctavian AndronicChristian-Alexander BungeOctavian BuiuChristine DémoréOctavian DanilaChristine DetrembleurOctavian DospinescuChristine DewiOctavian Narcis IonescuChristine HarendtOctavian PostolacheChristine Kowal ChinelliOctavio Gutierrez FriasChristine StraussOday Ibraheem AbdullahChristo K. ThomasOded MargalitChristodoulos KelirisOdilbek UrmonovChristof KaubaOfer BeeriChristoforos NtantogianOgnjen ArandjelovićChristoph BayerOguzhan TuysuzChristoph HintermüllerOh Seok KwonChristoph Hoog AntinkOhsuk KwonChristoph KralovecOihane Gómez-CarmonaChristoph LangeOjan GorjaniChristoph M. FriedrichOktay YilmazogluChristoph RascheOkyaz EminagaChristoph StachOla RingdahlChristoph WaldmannOlarik SurintaChristophe DelebarreOlav WerhahnChristophe DomingosOlayinka O. OgundileChristophe LohrOlcay AltintasChristophe MoyOldřich VyšataChristophe RosenbergerOleg A. MarkelovChristopher BaileyOleg A. ShlyakhtinChristopher BoyleOleg A. StepanovChristopher J. HegartyOleg IlliashenkoChristopher KentOleg LeonovChristopher RamezanOleg M. GradovChristopher ReiningOleg MorozovChristopher T. GoodinOleg PolishchukChristopher TaudtOleg SergiyenkoChristopher ZuidemaOleg ShiryayevChristos AthanasiadisOleg StukachChristos C. SpandonidisOleg V. ButovChristos DimasOleg V. IvanovChristos KontoyannisOleg V. ZakharovChristos KoulamasOleh BerezskyChristos L. StergiouOleksandr GordieievChristos M. MichailOleksandr O. KuznetsovChristos N. PanagopoulosOleksandr PylypovskyiChristos NikolopoulosOleksii TyshchenkoChristos NtanosOlexander BarmakChristos P. AntonopoulosOlga DymshitsChristos PapakostasOlga FragouChristos PappasOlga IbryaevaChristos RiziotisOlga JakšićChristos S. AntonopoulosOlga KorostynskaChristos TroussasOlga Maria Cristina BucoveţchiChrysoula BetsouOlga NabochenkoChryssoula DrouzaOlga SokolovaChuan ChenOlga SykiotiChuan WangOlga SysoevaChuan-Bi LinOlga V. KaravaiChuan-Chi LaiOlha BuchelChuang SunOlimpiu StoicutaChuangbo HaoOliver GiudiceChuang-Yuan ChiuOliver MichlerChuanheng SunOliver OziokoChuanjun HuangOliver StrbakChuanjun LiuOlivér TörőChuanqi TanOliver Winston LaytonChuanxin NiuOliverio J. SantanaChuanyun FuOlli JokinenChuanzhi DongOluyemi Peter FaladeChuchart PintaviroojOm Jee PandeyChuen-Lin TienOmar AlhazmiChu-Hui LeeOmar AlzubiChun ZhuOmar Andres Carmona CortesChun-An ChengOmar Bani MelhemChunbiao LiOmar DagdagChundong XueOmar DibChung Chen TsaoOmar Faruqi MarzukiChung Horng LungOmar Gutierrez NavarroChungkeun LeeOmar Hernández-GonzálezChung-Ping ChangOmar JanehChung-Wei KungOmar Longoria-GandaraChun-Ho WuOmar Mendoza-MontoyaChun-Hou WangOmar NiboucheChunhua GengOmar OlarteChunhui MaOmar Rodríguez AbreoChunjiong Zhang Omar Younis AlaniChunlei HuoÖmer Melih GülChunlei XiaOmer MujahidChun-Liang ChenOmer SadakChun-Liang YehOmer SaleemChunmiao YuanOmid GhorbanzadehChunna ZhaoOmid JahanianChunrong PengOmid MahdavipourChunsheng WuOmid ZobeiriChunsheng YangOmneya AttallahChun-Wei TsaiOmnia HamdyChun-Wei YangOnder AydemirChunxiu LiuOnder ErinChunxu QuOngard ThiabgohChunxu TangOnur ÖzbekChun-Yeon LinOrazio AielloChun-Ying HuangOrestis SchinasChuong NgoOrhan DagdevirenChuong V. NguyenOrhan OzgunerChurna BhandariOri RottenstreichChutisant KerdvibulvechOrlandino TestaChyan-Chyi WuOrlando Aguirre GuedesChybyung ParkOrtner MichaelCiara HeavinOsama A. OmerCícero CenaOsama OlabyCicerone Laurentiu PopaOsamah Shihab AlbahreyCid André Fidelis De Paula GomesOsamu KamiyaCid Marcos Gonçalves AndradeOscar AlonsoCihan BayindirOscar Andres VivasCíntia FrançaÓscar CarranzaCinzia CaliendoOscar Casanova CarvajalCioclea DoruOscar CastilloCiprian Briciu-BurghinaOscar De LucioCiprian DobreOscar Elias Bonilla ManruqueCiprian LapusanOscar F. AranedaCiprian LupuOscar Gil-CastellCiprian OrheiOscar Herrera-AlcántaraCiprian Petrişor PungilǎÓscar LópezCiprian Romeo ComşaOscar Mario Rodríguez-EliasCiprian-Radu RadOsman OrhanCiro ApollonioOsman Radwa AhmedCiro GioiaOsman UlkirClara Simon de BlasOsslan Osiris Vergara VillegasClaude CariouOswaldo Lopez SantosClaudia Alana ConstantinescuOswaldo MenendezClaudia CanaliOthman Bin MohdClaudia FerrarisOtman MrabetClaudia GirjobOto HaffnerClaudia Herrera-SiklodyOtoniel Mario López GranadoClaudia LermaOtto CorrêaClaudia LopesOu BaiClaudia MarziOuti JolankiClaudia PacurarOvidiu S. StoicanClaudia RinaldiOvidiu-Marcel MurârescuClaudia ScatignoOwais Ahmed MalikClaudio FerrariOxana VerkholyakClaudio LarosaOzgur Koray SahingozClaudio MarcheÖzkan UğurluClaudio NarduzziP. V. RamanaClaudio SavaglioPablo AquevequeClaus BørstingPablo Cienfuegos-SuárezCleber A. AmorimPablo CorralClécia C. B. GuimarãesPablo EscobedoClemens BeckerPablo Fernández-AriasClement LorkPablo Gómez-AbajoClement NyamekyePablo Lopez-CustodioClement NyirendaPablo Luis López EspíCleonilson Protasio De SouzaPablo Manuel Millán-MillanClifford LissendenPablo MartinezClifton HolmesPablo Palacios-JátivaClint HansenPablo Rodríguez-GonzálvezClio VossouPadmanabhan BalasubramanianClive D′SouzaPadmanathan KasinathanCocianu Catalina-LuciaPadmapriya PraveenkumarCodruta JaliuPadmini SankaramurthyColin DrummondPak Kwong ChanConcetta De StefanoPalanisamy RamasamyCong OuPalaniyandi KaruppaiyaCong QiuPallavee BhatnagarCong ShiPallavi GuptaCong YangPaloma de la PuenteConghui LiuPamayla DarbyshireConor MuldoonPamela ZontoneConrad RizalPan Kee BaeConsolatina LiguoriPanagiotis AlevrasConstantin CaruntuPanagiotis D. MichailidisConstantin NistorPanagiotis GkonisConstantin OanceaPanagiotis KarkazisConstantin VertanPanagiotis Karmiris-ObratańskiConstantin VolosencuPanagiotis KasnesisConstantin-Catalin DosofteiPanagiotis KatrakazasConstantine KotropoulosPanagiotis KogiasConstantine LukashinPanagiotis MichalisConstantine TarawnehPanagiotis PapakanellosConstantino Carlos Reyes-AldasoroPanagiotis Radoglou-GrammatikisConstantinos AngelisPanagiotis TrakadasConstantinos KoliasPanagiotis VlamosConstantinos PatsakisPanagiotis-Christos VassiliouCorinna SchmittPanayiotis KotzanikolaouCorinthias P. M. SianiparPandarasamy ArjunanCornel CobianuPandian PitchipooCornel IoanaPanga Narasimha ReddyCornelia MeckbachPangun ParkCorneliu DorofteiPangwei WangCorrado BoragnoPankaj DadheechCory SnyderPankaj KumarCosimo GentilePankaj SinghCosimo LupoPanmanas SirisomboonCosmas Ifeanyi NwakanmaPanos LoukopoulosCosmin Pompei DǎrabPantea KeikhosrokianiCostantino BalestraPantelis AngelidisCostanza Scaffidi AbbatePaola MazzantiCostas ChaikalisPaola SaccomandiCostas PanagiotakisPaola SansoniCostas S. ConstantinouPaolo BennaCostas VassilakisPaolo BertoncelloCostel Emil CotetPaolo BifulcoCourtney Elaine StewartPaolo BruschiCraig A. CoburnPaolo CasariCraig A. StauntonPaolo CastaldoCraig BartonPaolo CastiglioniCraig E. NelsonPaolo CrippaCreed JonesPaolo Di BellaCrescenzo PepePaolo E. SantangeloCrhistian Raffaelo BaldoPaolo FacciCrina Anina BejanPaolo Francesco ManiconeCristhian DuranPaolo GastaldoCristi MoldoveanuPaolo LonettiCristian AndrieseiPaolo MarconciniCristian AnghelPaolo MercorelliCristian AxeniePaolo MeriggiCristian BarzPaolo MignoneCristian ChilipireaPaolo NeriCristian CiureaPaolo PasqualiCristian ComanPaolo PetriniCristian FioriPaolo RussoCristian FosalauPaolo Stefano CrovettiCristian LaiPaolo TagliolatoCristian Lucian StanciuPaolo TripicchioCristian ManzoniPaolo ViscontiCristian MartinPaolo ZaffinoCristian OleaParameshachari DivakarachariCristian PolizianiParastoo FarniaCristian Ramirez-GutierrezParesh SaxenaCristian RotariuPari Delir HaghighiCristian ScheauParikshit DuttaCristian SimionParisa NaraeiCristian Tejedor-GarcíaParracino AntoniettaCristian VasarPartha Pratim RayCristian VendittozziPartha RoyCristiana Iulia DumitrescuParvathaneni Naga SrinivasuCristiana LarizzaParvathi ChundiCristian-Cezar PostelnicuParvez Ahmad AlviCristian-Dragoș DumitruParvez MahbubCristian-Gyozo HabaPascal DupuisCristiano D′AndreaPascal NicolayCristiano FidaniPascal ThubertCristiano Gonçalves PendãoPasika Sashmal RanaweeraCristian-Valeriu PatrichePasquale DaponteCristina AchimPasquale GiungatoCristina BaldissarriPasquale TommasinoCristina Bianca PopPatrice BellotCristina BudaciuPatricia Agudelo-RomeroCristina CirtoajePatricia ArroyoCristina Garcia-MunozPatricia Edith CamporealeCristina Gutierrez-SanchezPatricia HuestisCristina Maria Ribeiro CaridadePatricia KhashayarCristina Mihaela CampianPatricia López-GarcíaCristina NuzziPatricia Losada-PerezCristina PotrichPatrícia MartišováCristina RePatricia VazquezCristina RegueiroPatricio OrdazCristina SerbanPatrick A. HelmCristina Sorina StângaciuPatrick BoissyCristina Stolojescu-CrisanPatrick Chi-leung HuiCristina TuvèPatrick DumondCristóbal García ParientePatrick GioiaCsaba KorenPatrick HerwigCunjin XuePatrick HübnerCuong NguyenPatrick J. SkrodzkiCurtis WalkerPatrick Marques CiarelliCushla McGoverinPatrick WongCyril RonPatrik FlegnerCyrille MigniotPatrik KamencayCyrille RichardPatrik ŠargaCzesław DyrczPatrizia LivreriD. Arul KirubakaranPatrizia VizzaD. SaraswathiPatrizio MarianiDa Yang TanPatrycja WyszkowskaDabetwar ShwetaPatryk ZiółkowskiDac-Binh HaPattnaik Prasant KumarDadmehr RahbariPau Climent-PerezDaehan KwakPau Climent-PérezDaeho KimPau Redón LurbeDae-Hyun JungPaul C. OkonkwoDaejin ParkPaul CrockerDae-Ki KangPaul D. Rosero-MontalvoDagang LiPaul Eric DossouDagmar PavlůPaul FaragoDahou AbdelghaniPaul HemerenDa-Hua WeiPaul RuffinDaigo TerutsukiPaul SchattanDairi AbdelkaderPaul SellinDaisuke MatsuuraPaul Soto RodriguezDaisuke MurakamiPaul StaceyDaiva MikucionienePaul SummersDajun WuPaul TucanDalbert Matos MascarenhasPaula Corte-LeonDalei WangPaul-Émile BoileauDalia ElsheakhPaulina KrasnodębskaDalia NandiPaulina ParchetaDalibor CiprianPaulina RusanowskaDalibor L. SekulicPaulina Sierra-RosalesDalila DurãesPauline M. LefebvreDalin ZhangPaulius PaleviciusDalit Shach-PinslyPaulius SkačkauskasDalmiro MaiaPaulo A. Raymundo-PereiraDalton Martini ColomboPaulo CaldasDamian J. J. FarnellPaulo CostaDamian LedzińskiPaulo DiasDamian PęszorPaulo E. CruvinelDamiano PerriPaulo FloresDamien BrezulierPaulo Jorge CoelhoDamien ChablatPaulo Jorge Lourenço NunesDamien LeducPaulo M. MendesDa-Ming YangPaulo MonteiroDamir MadjarevićPaulo Sousa GilDamjan StrnadPavan ChaturvediDamjan VlajPavan Kumar B. N.Dan B. MarghituPavan PoudelDan ButnicuPavel AtanasoaeDan DobrotăPavel D. BobrovDan GotaPavel FialaDan IstratePavel GotovtsevDan Jeric Arcega RustiaPavel HazdraDan LiPavel KrasnovDan LuoPavel LafataDan MaromPavel LoskotDan NeculoiuPavel LyakhovDan PescaruPavel MlejnekDan SelisteanuPavel NosovDan VasilachePavel OtřísalDan YangPavel ProsrDana BadauPavel SedaDana CaultonPavel SegecDana ŠišmišováPavel StarovoitovDan-Alexandru SzaboPavel StasaDana-Mihaela PetroșanuPavel TofelDan-Cristian PopaPavel TuralchukDaneilo GomesPavlo LykhovydDanfeng HongPavlos LazaridisDanfeng SunPavol HelebrandtDang Ngoc Hoang ThanhPavol KajánekDangdang ShaoPavol LipovskýDanial KarimiPavol SokolDanial MohammadzadehPavol TanuškaDanial RoshandelPawan PathakDanica RosinováPaweł BoguszDaniel A. R. ChavesPaweł BorkowskiDaniel AmoPawel BurdziakowskiDaniel Basso FerreiraPaweł ChmielarzDaniel BigmanPaweł CichoszDaniel Boari CoelhoPawel D. DomańskiDaniel Bonilla LiceaPaweł DolibogDaniel Carnieto TozadorePaweł DroździelDaniel Chagas Do NascimentoPaweł HerbinDaniel CondurachePaweł JabłońskiDaniel Costa RamosPaweł JanikDaniel CremonsPawel KasprowskiDaniel DudaPaweł KomadaDaniel Ferrández VegaPaweł KrzaczekDaniel Filipe AlbuquerquePawel LadoszDaniel Flores TapiaPaweł LigęzaDaniel FuentesPawel LinekDaniel G. CostaPaweł LonkwicDaniel GroßmannPawel MadejczykDaniel HernandezPaweł MazurekDaniel HoffmannPaweł ModrzyńskiDaniel Iwao SuyamaPaweł NowakDaniel J. PlewsPaweł PawłowskiDaniel JancarczykPaweł PijarskiDaniel Jauregui-VazquezPaweł PiskurDaniel KrajzewiczPaweł RosaDaniel KramerPaweł RzucidłoDaniel LealPawel S. DabrowskiDaniel MaposaPawel SkokowskiDaniel MarconiPaweł SkruchDaniel Martinez-MarquezPaweł StefaniakDaniel MayerPawel StrumilloDaniel Morinigo-SoteloPaweł SzaroDaniel Mota-RojasPaweł SzcześniakDaniel Octavian MelintePawel TysiacDaniel Pérez-LópezPawel WegrzynDaniel PinardiPaweł WeichbrothDaniel PoenarPaweł WierzbaDaniel R. FesenmaierPaweł WronaDaniel RamosPawel ZajacDaniel RittschofPaweł ZiolkowskiDaniel Segovia VargasPaweł ZmarzłyDaniel SilvestrePayam SarirDaniel SolaPeđa MihailovićDaniel SweeneyPedro Aires MontenegroDaniel TonelliPedro ArceDaniel Tudor CotfasPedro Bertemes-FilhoDaniel VanekPedro C. LallanaDaniel Zhengyu HuangPedro C. Santana-MancillaDaniela De BartoloPedro Carmona MarquesDaniela DebonePedro CostaDaniela DragomanPedro CoutoDaniela FrankePedro CruzDaniela PerdukováPedro Cuesta-ValiñoDaniela PöhnPedro D. Frazão CorreiaDaniela-Elena PopescuPedro D. VazDaniele BorzelliPedro Dinis Loureiro SalgueiroDaniele CafollaPedro FonsecaDaniele CartaPedro GonçalvesDaniele ChiriuPedro Henrique Lopes SilvaDaniele CiofiniPedro Javier Herrera CaroDaniele E. LucchettaPedro Jorge CaridadeDaniele FarnesiPedro José Bernalte SánchezDaniele FunaroPedro M. WightmanDaniele GiansantiPedro MateusDaniele MasaronePedro Melo-PintoDaniele PannonePedro Mendonça Dos SantosDaniele PiconePedro Miguel RodriguesDaniele PiscitelliPedro MorouçoDaniele RossiPedro Oliveira Conceição JuniorDaniele VellaPedro Pablo Garrido AbenzaDanielli Araújo LimaPedro Pablo González-PérezDaniil N. BratashovPedro Paulo Menezes ScariotDaniil P. AksenovPedro RibeiroDanijel KorzinekPedro Silva GirãoDanijela BaricPedro SumanDanil IvanovPedro Vargas-ChableDanila GermanesePedro Vega-JorqueraDanilo D′AngelaPegah BagheriDanilo De RossiPei JiangDanish JaveedPei Song CheeDanish KhanPei-Chen WuDanny BrouardPei-Chi HuangDanny D. MannPeida HuDansheng WangPeiman ValipourDante Mujica-VargasPeipei LiuDanting YangPeipeng XuDanuta MiedzińskaPeiquan JinDanyu WuPeitao WangDao GongPeiwu QinDao Nguyen KhoiPeixiao WangDaoye ZhuPei-yao ChengDapeng YangPeiyuan ZhouDaphne GeersePejman RezaeiDarcy BullockPekka JounelaDaria BylievaPekka SiirtolaDaria HemmerlingPelin AnginDaria TishkevichPenelope IoannidouDaria WotzkaPeng Cheng WangDario AntonelliPeng FangDario AssantePeng GuoDario Di GiuseppePeng HangDarío GarigliottiPeng HuDario GioiaPeng HuangDario PasiniPeng LanDario TagliaferriPeng LiuDario VangiPeng LuDarius MigilinskasPeng MiaoDariush Rezaei OchbelaghPeng QinDariusz BoguszewskiPeng WuDariusz BuchczikPeng ZengDariusz GozdowskiPeng ZhangDariusz HorlaPengcheng ZhangDariusz JamrózPengfei JiaDariusz MakowskiPengfei LiuDariusz MichalakPengfei XuDariusz MikołajewskiPengfei ZhangDariusz PazderskiPengjiao JiaDariusz SawickiPengmin PanDariusz SzpicaPengpeng ShiDariusz UlbrichPengpeng ZhiDariusz WięcekPengyu WangDarko AndročecPensieri SaraDarko GalinecPere Marti-PuigDarko HuljenićPérez-Suay AdriánDarwin KurniawanPericle PerazzoDarya KlyamerPeristera BazianaDashuai WangPerumal EswaranDat NgoPervin DemirDave GilmorePeshal NayakDavid Ahmedt-AristizabalPetar B. PetrovicDavid Antonio Gómez JáureguiPetar KochovskiDavid BeserraPetar RadanlievDavid Boullosa-FalcesPete CulmerDavid CameronPeter A. GloorDavid CantusPeter BarcikDavid Cárdenas-PeñaPeter BelobrovDavid Chunhu LiPeter ChanDavid Cuadrado-CallePeter FinkelDavid EhrePeter FrickDavid G. GobbiPeter GirovskýDavid Gomez AndresPeter GorelkinDavid Granados-LiebermanPeter HeskethDavid HernandoPeter HinterdorferDavid J. KukulkaPeter HubinskyDavid JohnsonPeter KazanzidesDavid LeglandPeter KönigDavid López-IglesiasPeter KorbaDavid Luciano RosalenPeter KraemerDavid Manzano SánchezPeter LevineDavid MartinezPeter MorleyDavid MelendiPeter PoórDavid MishnePeter RogeljDavid MolyneuxPeter RolfeDavid Moreno MolinaPeter SandbornDavid Naranjo HernándezPeter SarcevicDavid O′SullivanPeter StützDavid PerpetuiniPeter SykacekDavid RodriguezPéter TamásDavid Ruiz GraciaPeter TrúchlyDavid Sanchez MonteroPeter ZollikerDavid SanioPetr BlechaDavid Sarabia-JácomePetr BouchnerDavid ScaradozziPetr DvořákDavid SedláčekPetr HlubinaDavid Serrano-GarcíaPetr HonzíkDavid ShapiroPetr HubacekDavid StratosPetr KudrnaDavid SziroczakPetr M. PivkinDavid T. FullwoodPetr PalackyDavid ThompsonPetr StastnyDavid ValientePetr VolfDavid VrbaPetra BudíkováDavid YeboahPetra VidnerováDavid ZumrPetre Daniel MatasaruDavide AstolfiPetri VälisuoDavide BorelliPetrica VizureanuDavide CalandraPetros DarasDavide ChiarellaPetros PatiasDavide ForcelliniPetros S. BithasDavide MarinoPetru GhenucheDavide MoroniPeyman Roodgar SaffariDavide PapurelloPham Duy ThanhDavide RoccoPhan Gia HoangDavide Settembre BlundoPhilip D. ChilibeckDavide SpinelloPhilip GardinerDavide SpiritoPhilip KilbyDavod PorehPhilip MooreDavorin AmbrušPhilipp Del HougneDawid BruskiPhilipp FechtelerDawid PołapPhilipp M. SchollDawid WajnertPhilipp OleynikDawit AberraPhilippe BenechDayal WijayarathnePhilippe BrunetDayi ZhangPhilippe GueguenDa-Yin LiaoPhilippe SoyerDaynier Rolando Delgado SobrinoPhilippe VelhaDe GongPhillip MillarDe Rosal Ignatius Moses SetiadiPhillis Soek Po TengDe Xing LioePhuong T. NguyenDebadatta DashPhuong T. TranDebanjan DasPi-Chung WangDebarun SenguptaPier Giorgio SpazziniDebasis MaityPier Luigi MazzeoDebasish JanaPier Matteo BaroneDeblina BiswasPier Nicola SergiDébora Regina Schneider LocatelliPierluigi ConfuortoDedalo MarchettiPierluigi RealiDedy WijayaPierpaolo LoretiDeepak BalramPierpaolo PalestriDeepak BansalPierre AmbsDeepak GhimirePierre ChavelDeepak KukkarPierre GiovenazzoDeepak Kumar DewanganPierre-Jean BouvetDeepak MishraPierre-Jean GauthierDeepak SinghPierre-Martin TardifDeepika KoundalPierre-Yves FoucherDeepti SharmaPierre-Yves Le BasDeGui SunPiervincenzo RizzoDegui YangPiet VerwilligenDeisy ChavesPieter De BeuleDejan DrajicPietro MaranoDejan G. ĆirićPietro MonforteDejan JokicPietro MonsurròDejan MilićPietro PiazzollaDelia FariasPietro PicernoDella Ragione LiviaPietro RanieriDelong HePietro ScanduraDelowar HossainPilar Alfageme-GarcíaDelphine ChadefauxPil-Sung YangDemba DialloPiltyay StepanDemetrio IeroPing JiangDemetrios E. TsesmelisPing LuDemi AiPing WangDemian Antony D′MelloPing ZhouDemis BassoPing-Hei ChenDemóstenes Zegarra RodríguezPing-Hui LeeDeng WangPingping ChenDeng-Guang YuPin-Ju TsaiDengpeng XingPinliang DongDeni Librado Torres-RomanPiotr ArtiemjewDenio DuartePiotr BonieckiDenis A. ZherdevPiotr BorkowskiDenis BourgeoisPiotr BorowikDenis ButusovPiotr DąbrowskiDenis ChikurtevPiotr DeuszkiewiczDenis Delisle RodriguezPiotr GajewskiDenis GuilhotPiotr GawdaDenis MartynovPiotr GazdaDenis N. SidorovPiotr GorniakDenis RosárioPiotr J. DurkaDenis TihonPiotr Jankowski-MihułowiczDenis V. MakarovPiotr JaworskiDenis Vasil′evPiotr KonopelskiDenise BellisarioPiotr KrauzeDenise Paschoal SoaresPiotr KulickiDenise WilsonPiotr KuropkaDenise-Penelope N. KontoniPiotr KuwalekDeniss StepinsPiotr LadyzynskiDeniz EkizPiotr LesiakDeniz GençağaPiotr LipińskiDennis OdijkPiotr MarkowskiDeqing KongPiotr MichalakDeqing ZhaiPiotr MillerDer-Chyuan LouPiotr MiluskiDerdouri AhmedPiotr MorasiewiczDerek Kwaku Pobi AsieduPiotr PorwikDerlis GregorPiotr RybackiDeron LiangPiotr S. SzczepaniakDerrick M. MottPiotr SobotkaDerya BirantPiotr TworekDesiderio Cano PorrasPiotr WitczakDésiré RasolomampiononaPiotr WrzecionoDesmond J. FitzGeraldPiotr ZawadzkiDeuk Jin ParkPisana PlacidiDeukhee LeePiyanut PinyouDevarshi ShahPiyush Kumar ShuklaDevianti DeviantiPlaban DebDewanto HarjunowibowoPlácido Moreno BeltránDeyi ZhouPlácido Rogerio PinheiroDeyun ZhouPo-Chun HsuDezheng YangPo-Chyi SuDezhong KongPoh Foong LeeDezun ZhaoPohl LászlóDhafer MezghaniPolona CasermanDhakshin RamanathanPolona Pavlovčič-PrešerenDhanamjayulu ChittathuruPolyzois SoumplisDhanasingh Sivalinga VijayanPonnusamy ThangarajDharmendra KumarPooja KamatDharmendra RajputPoornachandra SanjeevaDhasarathan VigneswaranPooya TaheriDhaya KanthavelPooyan AyarDhaya RamakrishnanPosen LeeDheyaa Jasim KadhimPoushali DasDhiah Abou-TairPo-Wen ChenDhiman DasPo-Yu KuoDhouha El HoussainiPrabal Datta BaruaDhruvajyoti RoyPrabhakar SathujodaDi JinPrabhat KumarDi LiPrabhishek SinghDi PengPrabhu SethuramalingamDi SunPradeeban KathiraveluDi WuPradeep KumarDi YunPradeep Kumar SinghDi ZhangPradip XavierDia ZeidanPradipta ChakraborttyDiana BerbecaruPradnya H. GhareDiana BorzaPragathi Priyadharsini BalasubramaniDiana C. Toledo-PérezPrahlad Kumar RouthDiana Francisca AdamattiPrakash MohanDiana ManjarresPramit MazumdarDiana RossiPramod K. B. RangaiahDiana-Margarita Córdova-EsparzaPranav A. BhounsuleDianbiao DongPranav GuptaDianpeng QiPranav J. ParikhDíbio BorgesPranav Kumar SinghDidi ShePrasanalakshmi BalajiDidier PradonPrasanna BhaleraoDiego BetancourtPrasant Kumar SahuDiego CalvettiPrasanta K. PanigrahiDiego Fernández IglesiasPrashant MalikDiego Leonel Cadette DutraPrashant PandeyDiego MejiaPrashant S. DeoreDiego MinciacchiPrashant ShrivastavaDiego Muñoz MarínPrateek BenhalDiego RenzaPrateek NallaboluDiego SušanjPrateek NegiDieter GollmannPratesh JayaswalDiganta DasPratheepa RasiahDigendranath SwainPratik M. PataniyaDilana Hazer-RauPratip K. BhattacharyaDilbag SinghPraveen ChoppalaDilovan ZebariPraveen Kumar DontaDiluka MoratuwagePraveen Kumar MalikDima CheskisPraveen Kumar Reddy MaddikuntaDimitra N. PapadimitriouPraveen LalwaniDimitri BelliPraveer SinghDimitri ThomopulosPrawin JayakumarDimitri WatelPredrag IvanisDimitrios A. ExarchosPredrag PetrovićDimitrios A. PatikasPreetha SharanDimitrios AmaxilatisPreeti GuliaDimitrios FilippiadisPreeti KumariDimitrios I. DoukasPreetom NagDimitrios KaterisPreety AhujaDimitrios KatsavelisPrem RanjanDimitrios KonstantinidisPremysl HudecDimitrios KosmanosPřemysl LubalDimitrios KoulocherisPrincy RandhawaDimitrios KoutmosPringgo Widyo LaksonoDimitrios LiarokapisPritam BoseDimitrios LoukatosPriyanka KarnatiDimitrios NikolopoulosProbir Kumar PalDimitrios P. NikolelisProdromos-Vasileios MekikisDimitrios PapageorgiouProloy Taran DasDimitrios PliatsiosProsanta GopeDimitrios StamovlasisPrzemysław Falkowski-GilskiDimitrios VrachatisPrzemysław OtomańskiDimitrios-Nikolaos PagonisPrzemysław PodulkaDimitris AmpeliotisPrzemyslaw StpiczynskiDimitris Dionissios KoutsourisPrzemysław TomalskiDimitris KaskaoutisPu MiaoDimitris KouisPu WangDimitry VasilyevPui Wah (Veni) KongDimo StoilovPukar MaharjanDimos TriantisPulak BhushanDimosthenis GiokasPumidech PuthongkhamDina IbrahimPurushotham SwarnalathaDinesh Babu DuraibabuPushpa Raj JoshiDinesh BhatiaPushpendra SinghDinesh Kumar SahPuspanjali MohapatraDinesh PatelPuttamadappa C.Dinesh RokayaPyke TinDinesh RotakePyo-Woong SonDinesh Veeran PonnuveluQaiser RiazDing NingQasem Abu Al-HaijaDing WangQasem Ahmed DrmoshDing-Quan NgQasem Al-MdallalDingwen ZhangQasim AhmedDinh-Thuan DoQasim UmerDinko OleticQazi Mazhar Ul HaqDino DobrinićQi WangDinos FerentinosQi XieDinu DraganQi ZhangDiógenes Cecilio Da Silva, JrQian HuangDiogo DuarteQian LiDiogo GomesQian LiuDiogo MattosQian XuDiogo RodriguesQianchuan ZhaoDiomadson BelfortQiang DuanDionisio RodriguezQiang LaiDionisios YoulatosQiang LiuDionysia-Georgia PerperidouQiang XuDionysios TheodosisQiang YuDios FedericoQiang ZhangDipankar BeheraQianggang WangDipankar MaityQiangyu ZengDipankar MitraQianqian ZhangDippong ThomasQichang MeiDirk H. OrtgiesQidi LiuDirk RueterQiguang HeDirk W. LachenmeierQiguo RongDirk ZeussQijia GuoDitta UngorQijin ZhangDivij BhatiaQijing LinDivya UtrejaQiliang LiDiyan DimitrovQilin HuaDjordje CantrakQing GuoDmitri MogilevtsevQing LuDmitri V. LouzguineQing YeDmitrii O. GlushkovQing YuDmitrii SolomitckiiQing ZhangDmitriy A. MartyushevQingchun GuoDmitriy A. PokamestovQingchun LeiDmitriy A. YagodnikovQingdang LiDmitriy TopolskyQingdong OuDmitry AydarovQingfeng CaoDmitry BaimelQinghai YangDmitry KaplunenkoQinghe ZhengDmitry KirsanovQinghua HeDmitry KorzunQingjun LiDmitry LazurenkoQingjun ZhangDmitry O. DolmatovQinglan DingDmitry RolduginQinglei GuoDmitry SitnikovQinglin WangDmitry SkvortsovQinglu MaDmitry TarasovQingquan LiDmitry TarkhovQingsong ZuoDmitry TelyshevQingwei LiaoDmitry YukhimetsQingyang WeiDmytro B. ButQinjun WangDmytro FishmanQinlin CaiDmytro TatarchukQinru SunDobrea Dan-MariusQinzhuo LiaoDoğan ÇelikQiong RanDohoon KimQiongshui WuDollaporn AnopasQipei (Gavin) MeiDolores María Llidó EscrivaQitao TanDolores R. SerranoQiugang LuDomenico CannatàQiwen QiuDomenico CiuonzoQizhen SunDomenico Di MauroQonita Kurnia AnjaniDomenico FlagielloQuan Bing Eric LiDomenico GattusoQuan ChaiDomenico PrisaQuan GuDomenico SurianoQuan JinDomenico UrsinoQuande YuanDomingo MarreroQuang Hung DoDomingos BarbosaQuang Hung TranDomini WeikertQuang-Cherng HsuDominik LenzQuero GiuseppeDominik OlsewskiQun CaoDominik RybarczykQun WeiDominik StrzalkaQuoc Bao DiepDominik TomaszukQuoc Bao LeDominik ZoekeQuoc-Dong HoangDominika Boguszewska-MańkowskaQuoc-Hung PhanDominika DąbrowskaQusay HassanDominika KaczorowskaQutaiba AlasadDominika SiwiecQuy KhanhDominika ZiajaR. NarayanamoorthiDominique LeporeR. Newlin ShebiahDomonkos VargaR. V. VinuDonald BaileyRaaid AlubadyDonald MossRabee HagemDonald R. ReisingRabha W. IbrahimDonatella DarsenaRabia RiazDonatella DominiciRabindra BistaDonatella PuglisiRabiu Muazu MusaDonato Hernández FusilierRachel VitaliDonato Luna MorenoRachid BenouiniDong GaoRachid SaadaneDong HeRachid ZagroubaDong HuangRadek StrnadDong Hyuk ParkRadhey Shyam JangidDong JiangRadoslav BeňušDong KimRadoslava KralevaDong LiRadosław BelkaDong LiuRadoslaw MiskiewiczDong Min KimRadostina A. AngelovaDong Sik KimRadovan HolubekDong WangRadovan MetelkaDongdong ChenRadovan ŠomplákDongdong ZhouRadu C. RacovitaDongfang LiRadu CiorapDonggu ImRadu ComesDong-Gyu LeeRadu Hanzu PazaraDonghee ParkRadu Paul MihailDongheon LeeRadu PetruseDonghoun LeeRadu PietraruDonghu NieRadu-Eugen BreazDonghua WangRaed AlsaqourDonghui YangRaed AlsiniDonghyun KangRaees Ahmad KhanDongil HanRaees SwatiDongil ShinRafael Abrantes PenchelDong-Jin KimRafael Aguilar-GonzalezDong-Jun YeomRafael CasadoDonglai ZhangRafael Corsino Rafael CorsinoDonglai ZhongRafael Escarela-PerezDongliang XiaoRafael EstepaDongmahn SeoRafael G. González-AcuñaDongping XiaoRafael GiustiDongsheng LiRafael Maestre-FerrizDongsheng LuoRafael Martos BuoroDongsheng YangRafael Perazzo Barbosa MotaDong-Shik KimRafael T. de Sousa, Jr.Dongsoo HanRafael Vargas-BernalDong-Soo KwonRafaela BortoliniDongwoo KimRafal BurdzikDongxue ZhaoRafał DreżewskiDongyi WangRafał GrądzkiDongyu ZhangRafał J. DoniecDong-Zhou ZhongRafał K. WyczółkowskiDoo-Hee ChangRafał KapelkoDora Luz OjedaRafał KaźmierczakDora M. BallesterosRafał KobyłeckiDora RoqueRafał KowalikDorian CojocaruRafał MelnikDorian J. BurnetteRafał MłyńskiDorian VerdelRafał ObuchowiczDorijan ŠpikićRafal SkowrońskiDorin BeuRafał SzczepańskiDorin BicăRafał SzukiewiczDoron GerberRaffaele CarliDoroshenko OlgaRaffaele MarinoDorota Bielińska-WążRaffaele MatteraDorota LewczukRaffaele SalernoDorota PawlusRaffaele SolimeneDorota StachowiakRaffel BogenfeldDorotea KovačevićRafik HamzaDoru Florin ChiperRaghuvir ParmarDoru-Laurean BaldeanRagini SinghDoru-Nicolae StanescuRaheem Kadhim AjeelDoston KhasanovRahisham Abd RahmanDouglas A. AddlemanRahman AliDouglas BarbinRahul Kumar SinghDouglas G. GoodinRahul RadhakrishnanDouglas M. GoltzRahul SharmaDouglas O′ShaughnessyRaimir Holanda FilhoDouglas Paulo Bertrand RenauxRaimon CasanovaDouglas SingletonRainer HipplerDounia ElfadilRainer TutschDovan ThomRaj VinnakotaDowan ChaRaja BalaguruDo-Youn LeeRajagopal MaheswarDoyoung KimRajakumar RamalingamDragan D. MilašinovićRajan KadelDragan Marinkovic (Germany)Rajani SinghDragan Marinković (Serbia)Rajat M. MahapatraDragan PamučarRajat MehrotraDragan RanđelovićRajavel KrishnamoorthyDragana BartolićRajdeep Kumar NathDragana PerićRajeev AgrawalDragana Ž. KrstićRajeev K. RanjanDrago PupavacRajeev KumarDrago StrleRajeev PadbhushanDragoljub GajicRajeev TiwariDragos ArotărițeiRajendirane RajmohanDragos D. TaralungaRajendra DhakalDragos EneRajendra Kumar KhadangaDragoş Ioan SǎcǎleanuRajendra PatrikarDragos IsvoranuRajendra Prasath PalanisamyDragos VicoveanuRajesh AnbazhaganDragoslav NikezicRajesh BiswalDragutin LisjakRajesh GuptaDražan JozićRajesh KaluriDrew KernRajesh KumarDror MalkaRajesh SinghDrozdstoi StoyanovRajeshwara Chary SriramadasuDu HuynhRajiani IsmiDua OezsoyluRajit NairDuangrudee KositgittiwongRajiv Kumar SharmaDuarte FernandesRajiv SharmaDubravka SladićRajiv SinglaDubravko RogaleRajkishor KumarDuc-Duy HoRajkumar BandiDuc-Phuc NguyenRajkumar SainiDuc-Thinh PhamRajkumar Singh RathoreDuke BulanonRajneesh Kumar MishraDuk-Sun ShimRajni JainDulani Apeksha MeedeniyaRaju AnandDulce María Romero-AyusoRajveer Singh YaduvanshiDumitru CiolacRajvir YadavDumitru OlaruRajwali KhanDumitru-Clementin CercelRakesh AmetaDung Nguyen TrongRakesh Chandra JoshiDuo ShengRakesh ChaudhariDuo Wai Chi WongRakesh Nath TiwariDuong LeRakesh RajaboinaDurdu GuneyRakesh ShiradkarDurmuş KoçRakesh SinghĐuro BarkovićRakeshkumar MahtoDursun Zafer SekerRakhi TiwariDusan GajicRalf LübbenDusan MagaRalf LucklumDušan ŠimšíkRalf MethlingDušan StrušnikRalf StetterDusanka BoskovicRaluca Andreea FelseghiDuško DudićRam Bilas PachoriDuy-Hai VuRam NarayananDweepna GargRamabalan SundaresanDwueng-Chwuan JhwuengRamachandran ManickamDyah Kurniawati AgustikaRamachandran VenkatesanDyi-Cheng ChenRamaiah PrakashDylan HicksonRamalingam SakthivelDzati Athiar Bt RamliRamalingam UdhayakumarEanes Torres PereiraRaman KumarEbadat Ghanbari ParmehrRamanaskanda BraveenthEbenezer EsenoghoRamanathan LakshmananEbha KoleyRamandeep BehlEbrahim ElsayedRamasamy AsokanEbrahim GhaderpourRameez AsifEbrahim KaranRamesh Kumar MuralimanoharEbrahim MattarRamesh PeriasamyEbrahim Mohammed SenanRamesh RajuEbrahim SharifiRamez HajjEckhard KirchnerRami AlazraiEd HabtourRami HawilehEdelberto Franco SilvaRami MansourEdgar ArribasRamin Banan SadeghianEdgar GutiérrezRamin GhiasiEdgar Taya AcostaRamin JaberiEdiguer Enrique FrancoRamin KhatamiEdison Pignaton de FreitasRamiro Pillco ZoláEdith Osorio-De-La-RosaRamiro RoblesEdmund J. ZolnikRamiro Samano RoblesEdmund WidlRamiro VelázquezEdna BarrosRamon BarberEdna Dias CanedoRamon BragosEdnaldo J. FerreiraRamón CarrilesEdoardo De TommasiRamón Castañer BotellaEdoardo FaddaRamón González-CamarenaEdoardo GiustoRamunas AntanaitisEdoardo IdàRamy S. A. AfiaEdouard IvanjkoRamya ShenoyEdris A. F. MahtabRamzi MahmoudiEdson BimRan LiaoEdson Caoru KitaniRan LiuEdson de Miranda, Jr.Rana I. ShakoorEdson Roberto SantanaRana Muhammad AdnanEdson UrsiniRanajoy BhattacharyaEduard AgeevRandhir KumarEduard LlobetRangeet MitraEduard StoicaRaniyah WaziraliEduarda VieiraRanjan MishraEduardo Antonio SperanzaRanjeet ThakaliEduardo ArtalRanjit ShresthaEduardo B. FernandezRanka GodecEduardo Cabal-YepezRao Naveed Bin RaisEduardo CañeteRaouf Senhadji-NavarroEduardo Corral AbadRaoul R. NigmatullinEduardo CuestaRaoul ReiserEduardo De AlmeidaRapeepan PitakasoEduardo Ferro Dos SantosRaphael FalqueEduardo GodoyRaphael PfattnerEduardo Leonel BottegaRaphaël TrouillonEduardo Moreira Da SilvaRaquel Bouça-MachadoEduardo PalermoRaquel CervigónEduardo Peters RodríguezRaquel Leirós-RodríguezEduardo ScheerenRaquel P. F. GuinéEduardo SuárezRaquel Pérez-AloeEdward BormashenkoRaquel Ramirez-VazquezEdward GuillenRasa PauliukaiteEdward KozłowskiRashad AlfkeyEdward LawsRashad M. RamzanEdward MallenRashid Hanif AbbasiEdward MunteanRashid MehmoodEdward SabolskyRashmi Sharan SinhaEdward Stanisław OczeretkoRasiah ThayakaranEdward VerbreeRasool BukhshEdwin Valarezo AñazcoRasoul KhnadanEdyta KardasRateb JabbarEdyta KucharskaRatheesh Kumar MeleppatEdyta PasickaRathnakara MalateshaEero SalliRatko GrbićEfraín Santiago-RodríguezRatko ObradovicEfthimios KarymbalisRatko PilipovićEfthymios KarabetsosRaudel Sánchez-CampusanoEfthymios TripsanasRaúl Alberto Reyes-VillagranaEftihia NathanailRaul FizesanEftim ZdravevskiRaul LandEftychia PinakoulakiRaul OnetEgidijus KazanavičiusRaul Parada MedinaEgidio LofranoRaul RabinoviciEhab EssaRavi KumarEhab MohamedRavi PandiselvamEhrenfried ZschechRavi Reddy ManumachuEhsan EsmaeilzadehRavi S. HegdeEhsan HarirchianRavi ShankarEhsan HosseiniRavi Shankar ReddyEhsan KhoramshahiRavi Teja NallapuEhsan Kiani OshtorjaniRavinder KumarEhsan Madadi-KandjaniRavindra DhuliEhsan MohseniRavindra KumarEhsan NajafiRavindra ThakkarEhsan YaghoubiRavindranadh KoutavarapuEhsanul Hoque ApuRaviteja VangaraEhtisham LodhiRawshan AliEicher LowRay HintzEimuntas ParšeliūnasRay-Kuang LeeEithne DempseyRaymond K. ZhaoEjay NsugbeRaymond LowEkaterina KopetsRaymundo Buenrostro-MariscalEkaterina TolstayaRaza QaziEkaterina V. KunitsynaRazali YaakobEkaterini DelegouRazaque AbdulEkin OzerRazieh RazaviEl Houssein Chouaib HarikRazvan BocuEl Khalil CherifRăzvan Gabriel BobocElaheh Dalir AbdolahiniaRazvan Ioan PacurarElbaz I. AbouelmagdRazvan Liviu NistorElder Alpes De VasconcelosRăzvan MocanuEleazar Aguirre AnayaRazvan UdroiuEleazar Samuel Kolosovas-MachucaRazzaqul AhshanEleftheria SergakiRebeca EstradaEleftherios KofidisRebeca P. Díaz RedondoEleftherios ParaskevopoulosRebecca A. StatesElena Carlotta OlivettiRebeen Ali HamadElena DilonardoRecep GüneşElena DoynikovaRedha AliElena FerrettiRedmond R. ShamshiriElena HelereaRegio A. MichelinElena HusanuRegis BarilleElena KozlovaReham MostafaElena Mainer-PardosRehan SiddiquiElena MorottiReinis CimursElena OlivettiRemco BaggenElena PopovaRemedios Lopez-LiriaElena PotapovaRemigiusz RajewskiElena SavvateevaRemis BalaniukElena Simona LohanRemy NicolasElena SolovyevaRenan TrevisoliElena TomsikRenata Kelly MendesElena TorskayaRenata Lopes RosaElena TortaRenata RutkauskaiteElena V. EfremovaRenata SahaElena ZaitsevaRenata SistoElena-Daniela GrigorescuRenata Tobiasz-SalachEleni StaiRenata WagnerováEleni StrouliaRenata WłodarczykEleonora AlfinitoRenato GiacominiEleonora IottiRenato MaaliwEleonora Maria TronciRenato MiyagusukuEleuterio A. Sanchez RomeroRenaud LeturcqElgar BarbozaRencheng JinElham AhmadianRendi KurniawanElham RastegariRene E. JativaElias AlisaacRene HartanskyElias HamannRené J. F. MelisElie El HelouRene JarošEliete CaldeiraRené M. RossiEligiusz PawłowskiRene Romero-TroncosoEligiusz PostekRene Valdez-LazaldeEline van der KrukRenee ZahnowElio RomanoRenfeng MaElis KullaRen-Hung HwangElisa BassoliRenjie WangElisa DigoRenxin WangElisa KallioniemiRenzhe BiElisa PaneroResul DasElisa RojasReuben GovenderElisa ScalcoReuben TamakloeElisabet Huertas-HoyasRex X. TanElisabetta GenoveseRey David Vargas-SánchezElisabetta MartiniReza AlaviElisabetta PatronReza AlayiElisabetta PeriReza SadaatiElisabetta RonchieriReza ShahbazianElishai Ezra Tsur‪Reza ShahidiElkenany Brinse ElkenanyReza ZamaniEllen Siobhan MitchellRiaz Ullah KhanElmar TräbertRicard BoquéEloisa MacedoRicard VilaltaEloy Martinez-HerasRicardo Augusto Rabelo OliveiraElpidio RomanoRicardo Borda Lopes VieiraElrasheed Ismail M. ZayidRicardo C. MelloElsa Díaz-MontesRicardo Escobar-JiménezElsa Margarida GonçalvesRicardo GomesElsawaf AhmedRicardo KamikawachiEl-Sayed M. El-kenawyRicardo M. F. FernandesÉlvio GouveiaRicardo MalheiroElvira MaranesiRicardo Neves Correia SantosElżbieta JasińskaRicardo OliveiraElżbieta KaweckaRicardo PereraElżbieta KuberaRicardo Salazar-CabreraEmad Abdul-Hussain YousifRicardo Salido RuizEmad F. NewairRicardo Sánchez De MadariagaEman H. AlkhammashRicardo VardascaEmanuel PuschitaRicardo VergazEmanuela ProiettiRicardo Villanueva PolancoEmanuele CardilloRiccardo CarotenutoEmanuele Gabriel MargheritaRiccardo De GaudenziEmerson Rodrigo AlmeidaRiccardo GasbarroneEmil CazacuRiccardo OrtaleEmil DumićRiccardo RossiEmil FaureRicha SharmaEmil SmykRichard B. FairEmile MartincicRichard Demo SouzaEmilia BiffiRichárd FiáthEmília HroncováRichard GordonEmilia ScalonaRichard GreenwaldEmilia SiposRichard HopperEmiliano DescroviRichard J. PovinelliEmiliano Martínez-PeriñánRichard J. SheridanEmiliano ZampettiRichard John Bruce FrancisEmilio AndreozziRichard KotterEmilio Olías-RuízRichard MistrickEmilio OrsegaRichard TutwilerEmilio RapuanoRichard WatsonEmily HandRichie MoalosiEmin YildirizRick SturdivantEmine SezerRick VoßwinkelEmir PoskovicRida GadhafiEmma ColamarinoRidha GhayoulaEmmanouil G. SpanakisRidvan YamanogluEmmanouil SkondrasRidwan P. PutraEmmanuel Karlo NyarkoRigoberto Martínez-MéndezEmmanuel Merchán-CruzRihab HadjiEmmanuel MkpojioguRija ErvešEmmanuel Ojo AdemolaRiki PatelEmmanuel TopoglidisRiko ŠafaričEmmanuel TuyishimireRiku KlénEmmanuele PelusoRinaldo PaarEmmanuelle Abisset-ChavanneRio Prasetyo LukodonoEmrah ZerdaliRisely Ferraz AlmeidaEmre Ozan Emre Ozan PolatRisely Ferraz-AlmeidaEmrullah AcarRishabh DasEmrullah KorkmazRita Girão SilvaEn Te HwuRita KadikovaEnder BugdayRita LavikkaEnder SevinçRita Yi Man LiEndra JoeliantoRitamay BhuniaEnfeng DengRitayan MitraEngin CiftyürekRitesh Kumar SinghEngin EyceyurtRitu ChauhanEngin ZeydanRivero-Ángeles Mario EduardoEniye TebekaemiRiya ChatterjeeEnnio GambiRizwan Ali NaqviEnrica ZolaRoald M. TiggelaarEnrico FraccaroliRob B. N. ScharffEnrico G. CaianiRobbi RahimEnrico MilaneseRobert Alexandru DobreEnrico PetritoliRobert BarańskiEnrico StaderiniRobert BialikEnrico VezzettiRobert CarleerEnrico VirgilliRobert DickinsonEnrique A. Navarro-CambaRobert DonatelliEnrique AldaoRobert DuchnowskiEnrique BerjanoRobert DyjaEnrique BronchaloRobert FullérEnrique García-MacíasRobert G. OlsenEnrique Hernández-BalagueraRobert GrozaEnrique HortalRobert JasionowskiEnrique Mario SpinelliRobert KarpińskiEnrique Soriano-HerasRobert Konrad SzymczakEnrique Urrea-MendozaRobert KraaijEnwei SunRobert LiebichEnzo IulianoRobert LjubicicEnzo M. MumoloRobert M. NowakEpimitheas GeorgitzikisRobert MingeszEraldo OcchettaRobert OjstersekEraldo PassinoRobert PalovicsErcan IsikRobert PaszkowskiErcan KoseRobert RusinekErcan YilmazRobert Ryszard ChodorekErdal AydemirRobert S. BradleyErdem KüçüktopcuRobert SchmittErdem YazganRobert Simon SherrattErdenebayar UrtnasanRobert SitnikErdi AkmanRobert StarostaEren ŞahinerRobert StrongErez RibakRobert W. JacobEri Sato-ShimokawaraRobert W. PetersEric BusvelleRobert WaltersEric CampoRobert WójcikEric Chan-TinRoberta CalegariEric ChappelRoberta D′AurelioEric ChaumetteRoberta Maia SabinoEric DavisRobertas DamaševičiusEric de Souza GilRoberto Antonio MartinsEric FelliRoberto Augusto Gómez-LoenzoEric KirchnerRoberto Barcala-FurelosEric LefèvreRoberto CannataroEric LuiRoberto CasarinEric MaslenRoberto CorizzoEric Minwei LiuRoberto CorvajaEric MosierRoberto D′AmoreEric ParteliRoberto de FazioEric PetersenRoberto de J. León-MontielEric WadeRoberto De La RicaEric WebsterRoberto Di CapuaErica Isabella ParisiRoberto Di MarcoErica MenegattiRoberto FrancesconErica VoltaRoberto Giovanni Ramírez-ChavarríaErick Andres Perez AldayRoberto GuarinoErick OgamRoberto La RosaErick Paul Gutiérrez-GrijalvaRoberto Linares Y MirandaErik CrosmanRoberto LleraErik HeijneRoberto M. R. Di MartinoErik KépešRoberto Merino-MartinezErik SonnleitnerRoberto MerlettiErika MandaràRoberto PastoreErika RoviniRoberto PierdiccaErika ZemkováRoberto PilotErnest KurniawanRoberto RaffaeliErnesto A. Lagarda-LeyvaRoberto RomanielloErnesto D. R. Santibanez-GonzalezRoberto SaiaErnesto MarínRoberto Sanchis-SanchisErnesto Moya-AlborRoberto Sañudo OrtegaErol GelenbeRoberto StradaEros MontinRoberto VerucchiErsan ArslanRoberto Vincenti GattiErsan BaşarRobin Singh BhadoriaErsin DemirRobyn JonesErvin MoungRocco AlaggioErwin KristenRocco Antonio RomeoErzhu LiRocco CancelliereEsakki BalasubramanianRocco CitroniEshwar ThoutiRocio Alba-FloresEsmaeil HeydariRocío L. PérezEsmaeil MirzaeeRoderjan João GabrielEsmeidel LealRodica HolonecEsmeralda ChiorescuRodica OlarEsposito LucaRodica VoicuEstácio Tavares Wanderley NetoRodney Martinez AlonsoEsteban GarzónRodolfo HaberEsteban IngaRodolphe BoudotEsteban Tlelo-CuautleRodrigo CaliliEstefania Munoz DiazRodrigo Colnago ContrerasEstefanía Núñez CarmonaRodrigo F. O. PenaEster D′AccardiRodrigo FernándezEsther HontañónRodrigo RameleEsther RamírezRodrigo SantosEsther RebollarRodrigo Sávio PessoaEtienne HerthRodrigo Tumolin RochaEtienne LabytRoel JordansEuclides Lourenço ChumaRoemi FernandezEugen PfannRoer Eka PawinantoEugene EremchenkoRogelio de Jesus Portillo-VelezEugene KaganRoger DannenbergEugenia Fagadar-CosmaRoger WatkinsEugenia TomasiniRogério Luís De Carvalho CostaEugenio CavalloRohit TanwarEugenio Marino MerloRohtraud PichnerEugenio MartinelliRok DolenecEugenio MatteiRoland BouffanaisEugenio PucciRoland Kuen Ren ChenEugenio ZimeoRoland Van den TillaarEugeniusz RosolowskiRolf IngoldEui Su LeeRoman A. ZakoldaevEui-Jong KimRomán Alcides Lara-CuevaEui-Nam HuhRoman FedunovEuiwon BaeRoman GrillEulalia BalestrieriRoman PishchalnikovEun Jeong ChoRoman PopielarzEun Seo ChoiRoman RadilEun Soo ParkRoman RegulskiEung Joo LeeRoman RužarovskýEunho LeeRoman SkidanovEurico Farinha PedrosaRoman SzewczykEuripedes NobregaRoman SzostakEuripidis GlavasRoman TrochimczukEusebio BernabeuRoman V. KlyuevEusebio BugarinRoman VítekEusebiu Ilarian IoneteRoman ZeleznikEustace DogoRomeo MarianEva BagyinszkyRomuald BoucheronEva H. DulfRomualdas BausysEva Lorenzo IglesiasRomualdas NavickasEva MaiaRon AddieEva Maria García Del ToroRon Van SchyndelEva María Martínez-JiménezRonaldo C. PratiEva MelnikRong ChenEva Mihalyko-OrbanRong FanEva Vargas OrgazRong XiangEvan L. PannkukRong YinEvando AraújoRong ZouEvangelia KouidiRongrong LiuEvangelos AnastasiouRong-San JiangEvangelos BaltasRongwang ZhangEvangelos G. KarvelasRonnie O. Serfa JuanEvangelos MarkakisRonnie S. Concepcion IIEvangelos SkotadisRoohallah AlizadehsaniEvangelos SpyrouRoohollah TalebitootiEvelio Teijón-López-ZuazoRoozbeh Abedini NassabEvens EmmanuelRoque CalvoEverardo Granda-GutiérrezRoque José BerenguerEverardo Vargas-RodriguezRoque TorresEverton De MatosRosa BasagoitiEvgenia NovikovaRosa María Fernández AlcaláEvgenii KuzinRosa Maria Ferreira BaptistaEvgenii MaltsevRosa Maria Tapia-HaroEvgeniy M. LobovRosalinda InguantaEvgeny Gennadievich BazulinRosalynn NankyaEvgeny KarshakovRosamaria CapuanoEvgeny KhorovRosanna Suzuky PintoEvgeny MirkesRosaria CozzolinoEvgeny NikulchevRosario FedeleEvgeny PanidiRosario GrammautaEvgeny PikhayRosario OlivaEvgeny TrofimovRosario Schiano Lo MorielloEvghenii HareaRosca RaduEvizal Abdul KadirRosemary CollierEvren ÇatakRosendo Romero-AndradeEwa Burszta-AdamiakRoshan ThotagamugeEwa GieysztorRosli OmarEwa GorodkiewiczRoss UnderhillEwa Joanna GodzińskaRossella SvigeljEwa KorzeniewskaRostam Affendi HamzahEwelina Świa̧tek-NajwerRoua YoussefEyad HamadRouhollah AhmadiEyoab Zegeye TeshaleRoungsan ChaisricharoenEzequiel VidalRoushan KumarFabao YanRoveda LorisFabian Andres CastañoRoxana-Mariana BeiuFabian Andres Lara MolinaRoy MüllerFabian BraunRoy PembertonFabian SchrumpfRozenn AllanicFabien ChaixRuan Delgado GomesFabien ColonnierRubab SaherFabien FerreroRubén IzquierdoFabien MieyevilleRubén Martín-ClementeFabio BagarelloRuben MedinaFabio CaldarolaRuben San-SegundoFabio CortiRuben SpecognaFabio D′AndreagiovanniRuber Hernández-GarcíaFabio Di NardoRucha NatuFabio FerlazzoRuchire WijesingheFabio GoncalvesRudiati Evi MasithohFábio Henrique Rojo BaioRudiger GensFabio J. W. A. MartinsRüdiger PryssFábio Juner LanferdiniRudnik KatarzynaFabio MaggioRudolf AndogaFabio Mazzariol SanticiolliRudolf JanosFabio RoccaRuey-Beei WuFabio SantacaterinaRuey-Ching TwuFabio SartoriRuey-Kai SheuFabio SauliRuggero ReggianniniFabio SchiroRugved LikhiteFabio SolariRuhollah Taghizadeh-MehrjardiFabio TangoRui AntunesFabiola SpolaorRui ChenFabrice SaffreRui DilãoFabrice SeguinRui GuoFabricio Augusto Barone RangelRui HirokawaFabrício Braga Soares De CarvalhoRui LançaFabrício Daniel Dos Santos SilvaRui LiFabrizio CutoloRui LiuFabrizio de CaroRui MarcelinoFabrizio De VitaRui MartinsFabrizio GiulianoRui MinFabrizio MurgiaRui PengFabrizio PalmaRui S. MoreiraFabrizio SpanoRui Tadashi YoshinoFabrizio StesinaRui WangFabrízzio SoaresRui WuFacundo AguileraRui XuFadhil Al-AboosiRui YangFadi AlsaleemRui YueFadi Al-TurjmanRui ZhangFadi MohsenRui ZhaoFadis F. MurzakhanovRuifeng LiFady El-NahalRuifu ZhangFaezeh ShanehsazzadehRuinian JiangFahad AhmadRuiqing FanFahad Iqbal KhawajaRuiqing ShenFahad UsmanRuitao YangFahd AlhaidariRuiting WangFahed AlkhabbasRuizhi YangFaheem KhanRumana HossainFahimeh NazarimehrRung-Ching ChenFahmi F. MuhammadsharifRunjia DuFahmi KhalifaRunmin DongFaisal JamilRunsheng XuFaisal K. ShaikhRunwei LiFaisel TubbalRunze LiFaiz Ul MuramRuobing QianFaiza LoukilRuofei ZhongFaizah Safina BakrinRuoliang TangFaizan QamarRuoqi WeiFakhroddin NazariRuoxi SunFan DangRuoxiu XiaoFan JiangRuoyu SuFan LiRuoyu YangFan LiuRupesh Kumar ChikaraFan YangRushit DaveFang HouRuslan IbragimovFang WangRuslana ZiubinaFang XieRüstem KeçiliFangchen FengRuszczak BogdanFangfang JiangRute SantosFanglai ZhuRūtenis PaulauskasFanglong YinRuth M. MuthokaFanglue ZhangRuth MacKayFangmin SunRuth Prieto-MonteroFangming LiuRuth ShinarFang-Shii NingRuxandra TapuFangtong JiaoRuya XiaoFangwei ZhongRyad ZemouriFang-Yie LeuRyan A. KerekesFan-Hsun TsengRyan GibsonFanli MengRyan MattfeldFanny SpagnoloRyan McGrathFansheng ChenRyan RamirezFanyu LiuRyan T. WhiteFaouzi DerbelRyo KurachiFaouzi KammounRyo NatsuakiFaranak ShamsafarRyo TakedaFarbod KhameneifarRyota SakamotoFares RedouaneRyszard JanickiFarhad AhamedRyszard KorbutowiczFarhad HuseynovRyuhei YamadaFarhad Soleimanian GharehchopoghRyumin DmitryFarhan AminRyushi FujimuraFarhan MumtazRyuta SatoFariba GoodarzianS. K. Hafizul IslamFariba MollarasouliS. K. YadavFariba MostajeranS. L. BoranaFarid García-LamontS. M. ArifuzzamanFaris A. AlmalkiS. M. Hadi SadatiFarman AliS. M. Mizanoor RahmanFarman UllahS. M. Shatil ShahriarFarnaz FaridS. SmysFarooq Ahmed ShahS. SuprijantoFaroq KasimS. Zohreh HomayounfarFarrukh NadeemSaad Al-SaadiFarshad Moradi KashkooliSaad DarwishFarshad ShakeriaskiSaad Fawzi Al-AzzawiFaruk Fonthal RicoSaad MekhilefFaruk ÖzgerSaad MustafaFarzad KaramiSaad RehmanFarzad KianiŞaban ÖztürkFarzan Majeed NooriSabapathy ThennarasanFarzeen MunirSabato MelloneFarzin PiltanSaber KazeminasabFasheng WangSabina BarakovicFateh Mebarek-OudinaSabina BottiFatemah MofarrehSabina LicenFatemeh BabaeianSabina SzymoniakFatemeh RezaeiSabri AllaniFathi GhorbelSabrina DemarieFatih OzSabrina Gensberger-ReiglFatih Özkan AlkurtSachi Nandan MohantyFatih OzkaynakSachin KumarFatima SalahdineSachin Kumar GuptaFatima Zohra BenhaloucheSachin NavaleFatimah KhalafSachin SharmaFatma Dogan GuzelSachindra Kumar RoutFausta LoffredoSacit M. CetinerFausto GrandaSadaf Bashir KhanFawad KhanSadaf GilaniFaycal BouhafsSadanand PandeyFayez Tarsha KurdiSadegh ModiriFayna MammeriSadeq SalehFazal A. TalukdarSadeque Reza KhanFazal MuhammadSadik Ozgur DegertekinFederica CaselliSadiq AbdulhussainFederica CavalloSadiq AliFederica PardiniSadiq HussainFederica PianiSadok RezigFederica RolloSadullah ÖztürkFederica VerdiniSae-Bae NapaFederica VernuccioSaed MoradiFederica ZonziniSaeed Ahmed KhanFederico A. León-ZerpaSaeed AligholiFederico BecattiniSaeed AlsamhiFederico BertiSaeed AsiriFederico CarotenutoSaeed BabFederico Grasso ToroSaeed Hamood AlsamhiFederico MariniSaeed Mian QaisarFederico MoroSaeed OlyaeeFederico Vázquez HurtadoSaeed ShavvalpourFedor S. FedorovSaeid AminiFeezan AhmadSaeid RashidiFei HeSaeid SaneiFei KangSafak DoganFei LiSagadevan SureshFei LiuSagar JinachandranFei LyuSagar M. DoshiFei MaSagar ManeFei PanSagit ValeevFei QinSagnik ChakrabortyFei ShenSahameddin Mahmoudi KurdistaniFei WangSahar AndalibFei XiaSahin YildirimFei XingSahraoui DhelimFei YanSaid El KafhaliFeipeng DaSaid QuqaFeixiang ZanSaifur RahmanFeiyan HuSaifur Rahman SabujFeiyue TengSaikat DuttaFelice CrocettoSajad SabziFelice SiricoSajal ShrivastavaFelicia Ramona BirauSajid AliFelipe Augusto Pereira De FigueiredoSajid KhanFelipe CostaSajid MahmoodFelipe Da Rocha HenriquesSajjad A. GhauriFelipe Dos Santos LoureiroSajjad MehmoodFelipe JiménezSakdirat KaewunruenFelipe Muñoz La RiveraSakorn MekruksavanichFelix AdochieiSakthivel GnanasekaranFelix CarrielloSalah ElsayedFelix Hamza-LupSalah Hameed JumaaFelix LeachSalah I. YahyaFelix ScholkmannSalah ObayyaFen XueSalah Ud-Din KhanFeng ChenSalam KhamasFeng GanSaleem AhmedFeng GaoSaleh AbujaradFeng JiangSaleh AlyahyaFeng KangSaleh MobayenFeng KeSalekul IslamFeng LiSalem Al-AmeriFeng LiangSalih AlcayFeng QiSalih Serkan ArtaganFeng WeiSalil BharanyFeng YeSalil KaripottFeng YinSalim HeddamFeng ZhuSalim LamineFeng ZiangSalimeh KimiagarFengbin TuSalina Abdul SamadFeng-Cheng ChangSally McCleanFeng-Cheng LinSalma AbedelmalekFengchun ShuSalma ElsayedFenghe WuSalman A. KhanFenghua LiSalman AliFengman LiuSalman ArainFengqiang LiSalman GhafoorFengqing WangSalman KhalidFengyan YiSalmon LandiFengyu LiSalome OnianiFengyuan LiuSalvador Gutierrez SalcedoFerdinando ChiacchioSalvatore AlmavivaFerdinando Di MartinoSalvatore Antonio BiancardoFerdinando NunziataSalvatore BarbaFerenc BognárSalvatore EspositoFermín MiraSalvatore Flavio PileggiFernanda Cristina CorrêaSalvatore GalloFernando A. Monteiro SantosSalvatore GrazianiFernando AlmeidaSalvatore LombardoFernando BoavidaSalvatore MicalizioFernando CastañoSalvatore PastaFernando Castaño RomeroSalvatore RomanoFernando De Souza CamposSalvatore SerranoFernando ElySalvatore StivalaFernando FerreiraSalwa SaidiFernando Luis-FerreiraSam PeringFernando M. Araujo-MoreiraSam SalehianFernando MoreiraSaman FarhangdoustFernando Paz PellatSaman Yaghmaei-SabeghFernando RubioSamapd Kumar PandaFernando Sánchez LasherasSamarendra Nath SurFernando SerranoSambit BakshiFernando SotoSambuddha GhosalFernando Suparregui DiasSameena PathanFernando VeigaSameer AlaniFernando Vidal-VerdúSameer Chakravarthy VedulaFerry Wahyu WibowoSamer HanounFidel Núñez-RamírezSami MnasriFikret YildizSami MyllymaaFilipa JoãoSami MyllymäkiFilipa SousaSami ViskinFilipe AlvesSamih M. MostafaFilipe Arroyo CardosoSamineni PeddakrishnaFilipe CaldeiraSamir KhatirFilipe FerreiraSamir Kumar BandopadhyayFilipe NevesSamir MalakarFilipe SantosSamira BriongosFilippo BastianiniSamira NaghdiFilippo BattagliaSamit Kumar GhoshFilippo BiondiSamo RauterFilippo ChiarelloSampath BoopathiFilippo D′IppolitoSampath Dakshina Murthy AchantaFilippo DiaraSampath Kumar VenkatacharyFilippo FurfaroSampathkumar ArumugamFilippo GiannettiSamsul Ariffin Abdul KarimFilippo MantovaniSamuel Baraldi MafraFilippo PaganelliSamuel BurriFilippo SarviaSamuel Deslauriers-GauthierFilippo UbertiniSamuel Fernández CarneroFilippos VallianatosSamuel Kofi ErskineFilomena CarnideSamuel Montejo SánchezFilomena MaurielloSamuel SepulvedaFiorenzo MarinelliSamuel T. OrangeFirdous A. ShahSamuel WilsonFirooz SalamiSamuel YousefiFiroz Babu KadumudiSamuele De BartoloFisnik DalipiSamuele MarinelloFityanul AkhyarSamy BakheetFlávia Aparecida GraçaSana Ullah JanFlavio BarbosaSanaul HaqueFlavio BorfecchiaSanaz PournajafFlavio CapraroSanda Florentina MihalacheFlavio Celso TrigoSandeep JagtapFlavio EspositoSandeep KumarFlavio LupiaSandeep SamantarayFlavio MendoncaSandeep SharmaFlávio Soares Corrêa Da SilvaSandeep Singh SengarFleming GudaguntiSandeep SonyFlorenc DemroziSandeep SuryaFlorence LagardeSandi Baressi ŠegotaFlorentina GolgoviciSandi RahmadikaFlorentina-Daniela MunteanuSandip DuttaFlorentinus Dika Octa RiswantoSandip RoyFlorian EllsäßerSándor ValkaiFlorian Ion Tiberiu PetrescuSandra CanoFlorian KrompSandra Maria Gonçalves Vilas Boas JardimFlorian KunnemanSandra McFaddenFlorian Kurt PaternosterSandra PralgauskaitėFlorian LipsmeierSandra RodriguesFlorian SchaffSandra VerhagenFlorin Doru HutuSandrine BernardiniFlorin Dumitru PopescuSandrine LorimierFlorin MariasiuSandya SubramanianFlorin PostolacheSanford MeekFlorin RaduSang Bok KimFlorina S. IliescuSang Jun LeeFlorina UngureanuSang Kyoo LimFloris ErnstSang Min WonFoivos MichelinakisSangho ChoeFoivos PsarommatisSanghoon LeeFokko WieringaSangkil KimFolea SilviuSangman MohFons J. VerbeekSangmin LeeFortunato B. SevillaSangrok JinFotios GioulekasSangsoon LimFotis IliopoulosSangwoo LeeFouzi HarrouSanichiro YoshidaFow-Sen ChoaSanislav TeodoraFowzia AkhterSanja Mahovic PoljacekFranc DimcSanjay GhoshFranc JagerSanjay KumarFrancesc AlíasSanjay MauryaFrancesc SebéSanjay MisraFrancesca BorghiSanjiban RoyFrancesca CecchiSanjida MouryFrancesca Di TuroSanjoy DasFrancesca LeonardiSanjoy PaulFrancesca NonisSanju GuptaFrancesca PennatiSanjukta PookulangaraFrancesca RossiSankar SekarFrancesca VenneriSanskruti PatelFrancesca VenturiniSantiago CepedaFrancesco Alessio DicandiaSantiago Felici-CastellFrancesco AsciSantiago LainFrancesco BanterleSantiago RúaFrancesco BeritelliSantiago Tello-MijaresFrancesco BiancoSantiago ZabaloyFrancesco BuonamiciSantos VillafainaFrancesco CaridiSantosh Kumar MahtoFrancesco Carlo MorabitoSantosh PitlaFrancesco CauteruccioSantosh SubediFrancesco CenturelliSanzhar KorganbayevFrancesco ClementiSapam Ranjita ChanuFrancesco CrennaSapna JunejaFrancesco Di RenzoSaptarshi MukherjeeFrancesco DuranteSaqib SaeedFrancesco FidecaroSara A. WingesFrancesco FischettiSara CasacciaFrancesco FontanellaSara ComaiFrancesco FusoSara CoppolaFrancesco GaziaSara Gonizzi BarsantiFrancesco Giacinto LavaccaSara GullaceFrancesco GrecoSara InvittoFrancesco GuidiSara Palomo-DíezFrancesco GuzziSara PimentaFrancesco LamonacaSara PourjamalFrancesco MaitaSara SekkateFrancesco MarcheseSara StančinFrancesco MarchettiSara StolyarovaFrancesco MarianiSara ZanniFrancesco MarinelloSarah PriorFrancesco MartelliSarah TonelloFrancesco MassariSaravanakumar ArumugamFrancesco MoscatelliSaravanan KrishnanFrancesco P. ChieteraSarfraz HashimFrancesco PascaleSarinporn VisitsattapongseFrancesco PoggialiSarita Mazzini BruschiFrancesco PotortìSarma MutturiFrancesco RendeSaroj PanigrahyFrancesco SicurelloSartajvir SinghFrancesco TornabeneSaša AdamovićFrancesco VerdeSasa MrdovicFrancesco VilleccoSasa RajsicFrancesco ZanettoSascha FleerFrancine KriefSasikumar AsaithambiFrancis ChipemSathish Kumar SelvaperumalFrancis Morais Franco NunesSathish SanjeeviFrancis StevensSathishkumar Veerappampalayam EaswaramoorthyFrancisco Airton SilvaSathiskumar JothiFrancisco AlonsoSatinder GillFrancisco Alvarez RodríguezSatish KumarFrancisco Avila GomezSatish Mahadevan SrinivasanFrancisco Beltran-CarbajalSatish R. MoreFrancisco BulnesSatoru HiwaFrancisco C. Franco, Jr.Satoshi OsukaFrancisco CercasSatoshi SagaFrancisco Cuevas De La RosaSatoshi UchidaFrancisco Del Cerro VelázquezSattar DorafshanFrancisco F. C. RegoSattianadan DasarathanFrancisco FalconeSatya Prakash YadavFrancisco FernándezSatyabrata AichFrancisco Haces-FernandezSatyendra Kumar MishraFrancisco J. Aparicio-NavarroSaud AltafFrancisco J. González-CastañoSaúl E. Pomares HernándezFrancisco J. PeñaSaulius BaleviciusFrancisco J. Rodríguez-PulidoSaulius JuodkazisFrancisco Javier Ferrero MartínSaulius LukoševičiusFrancisco Javier Gimeno-BlanesSaurabh AgarwalFrancisco Javier González-CañeteSaurabh SahuFrancisco Javier González-CastañoSaurabh SinghFrancisco Javier MadrugaSaverio FarsoniFrancisco José Amaro-GaheteSavio PoovathingalFrancisco José Gomariz CastilloSavita AhlawatFrancisco Jose Torcal MillaSavitri BevinakoppaFrancisco Lopez-LinaresSayan Kumar RayFrancisco LozanoScott A. MitchellFrancisco Manuel Melgarejo MeseguerScott WhiteFrancisco Maroto-MolinaSean FainFrancisco MendezSebastià GalmésFrancisco Pérez-OcónSebastian BraunFrancisco PizarroSebastian BudzanFrancisco Rubén Castillo-SoriaSebastian DittmeierFrancisco TovarSebastian GłowińskiFrancisco Troncoso-PastorizaSebastian HeglerFrancisco ValeraSebastian Ion CeptureanuFrancisco W. De Sousa LimaSebastian IwaszenkoFrancisco ZampellaSebastian KozlowskiFrancisco-J. Renero-CarrilloSebastian OpalińskiFranck BadetsSebastian SaniukFranck MultonSebastian TschaunerFranco CallegatiSebastian WernerFranco CicirelliSebastião Simões CunhaFranco FrattolilloSebastien BalmeFranco PaveseSébastien CombéfisFranco ZanottoSébastien FontanaFrançois GrondinSebastien GabouryFrançois OuelletteSeda CamalanFrancois RameauSedig S. AgiliFrancois VidalSeema ShedoleFrank DenkSeghier TaharFrank DuschekSegun OlatinwoFrank NarducciSegundo Moisés Toapanta ToapantaFrank R. IhmigSegyeong JooFrank WoudaSeiichi TakamatsuFrank-Michael MatysikSeiji NishifujiFransiscus SuryadiSejin ParkFrantišek KlimendaSejoon LimFrantišek LopotŞekip Esat HayberFrantišek RacekSelahattin KosunalpFrantišek SynákSelcuk EkiciFrantisek TothSelim BozkurtFrantišek ZbořilSelim HanayFred BarezSelim SolmazFred E. DaumSelma RizvićFreddy OdilleSelma RizvičFrederic FontSenfar WenFrédéric GillotSeng LokeFrédéric LiSengul DoganFrederic MarinSenthil Kumaran SelvarajFrédéric MuttinSenthil Murugan NagarajanFrédéric VanderhaegenSenthilkumar MohanFrederik WernerSeok-Cheol KeeFrigyes Samuel RaczSeok-Joo KohFrouke HermensSeok-Kyoon KimFuchao LiuSeokwon YeomFuchuan DuanSeon Bong LeeFugo TakasuSeong Tak WooFuiboon KaiSeong Yong MoonFujian MaSeong-eun YooFujian TangSeongjin ChoiFulden Ulucan KarnakSeong-Jin KimFuliang YinSeong-Min LeeFulin LuoSeongwook LeeFulvio BabichSeong-Yoon ShinFulvio LavecchiaSeonyeong HeoFuming SunSepideh KhoshnevisFuminori MisaizuSerafeim MoustakidisFumio OgawaSerban Georgica ObrejaFuqiang LiuSerban MezaFurui WangSerdar DindarFusang ZhangSerdar ErolFushan TangSerena DattolaFu-Shiung HsiehSerena De SantisFutoshi KobayashiSergei EremenkoFuwen HuSergei I. IvanovFuxin DuSergei KhotiaintsevFuxing GuSergei KulinichFuyuan XiaoSergei PopovG. H. M. J. Subashi De SilvaSergei SavinG. Lloyds RajaSergei Sergei KurashkinG. M. ShafiullahSergei SoldatenkoGabor J. BartonSergei Stel′makhGábor KertészSergei Vasil′evich ChekalinGabor KosaSergejs KodorsGábor NégyesiSergey A. DenisovGabor SoosSergey AleshinGábor VértesySergey DizhurGabriel CarloSergey GudoshnikovGabriel Dias RodriguesSergey I. FomenkoGabriel FedorkoSergey KobtsevGabriel Gonzalez-SernaSergey KonovalovGabriel Kamilo Pantoja-BarriosSergey M. AndreevGabriel Lucian RaduSergey N. ShevtsovGabriel Mario BilmesSergey Pavlovich OsipovGabriel MurariuSergey PirogovGabriel NallathambiSergey ShchukinGabriel Nicolae PopaSergey ShevtsovGabriel OlteanSergey ShoydinGabriel Ramos-OrtizSergey SokolovGabriel Sanchez-PerezSergey SuslovGabriel VéluSergey UchaikinGabriel YbarraSergey VasilievGabriel YesufSergey VinogradovGabriela GabajováSergey YablochnikovGabriela LimaSergii BabichevGabriela Statkiewicz-BarabachSergio BarrachinaGabriela Valdés-RamírezSergio CalixtoGabriele BarellaSergio CelaschiGabriele MagnaSergio Cuevas-CovarrubiasGabriele MalavasiSergio DavidGabriele MelegariSergio De SalvatoreGabriele NardoneSergio Fuentes Del ToroGabriele PapadiaSergio Gonzalez-SevillaGabriele ScaliaSergio Iglesias-ParroGabriella BalestraSergio J. C. Do CarmoGabriella CasalinoSérgio Junichi IdeharaGabriella GiganteSergio López BuedoGabriella OlmoSergio Luiz StevanGabriella TognolaSergio QuinteroGabrijel OndrasekSergio RestainoGaddi BlumrosenSergiu Dan StanGael CloseSergiusz PatelaGael SebaldSergiy LavrenkoGaetano D. GargiuloSergiy YepifanovGaetano De LucaSerguei P. MurzinGaetano LicitraSerguei PrimakGaetano SantulliSerguei SekatskiGaffar HossainSerhat SimsekGai-Ge WangSerhii StepenkoGajdoš Kljusurić JasenkaSerioja Ovidiu TatuGaku ImamuraSerioja TatuGalina DemidovaSerosh Karim NoonGalina NikolovaSeung Hyun JeonGalina V. KurlyandskayaSeung Jae OhGalina V. PortnovaSeung-Bok ChoiGaliya Khasanovna KitaevaSeung-Ho LeeGalya Georgieva-TsanevaSeunghoon HwangGamani KarunasiriSeunghwan LeeGandhimathi VelusamySeunghyun HwangGanesh BabuSeung-Jong KimGanesh KarriSeungku KimGanesh KoyyadaSeungyub LeeGanesh Kumar SrinivasanSevda MohammadiGang HuSever-Gabriel RaczGang KouSevki CesmeciGang LiuSeweryn LipińskiGang MeiSeyed Ali GhahariGang WangSeyed Ali MosayebiGang WeiSeyed Amir HosseiniGang XiongSeyed Asaad HosseiniGang XuSeyed Ehsan HosseiniGangadhar AndaluriSeyed Mohammad RazaviGao LiSeyed Mojtaba Marvasti-ZadehGaobo XuSeyed Mostafa SiadatmousaviGaochang WuSeyed Saeid MohtasebiGaofeng ZhengSeyed Vahid Razavi TermehGaoge HuSeyed Yahya NikoueiGaopeng LuSeyedali MirjaliliGaoyang LiSeyed-Ali Sadegh-ZadehGaoyang ShanSeyyed Sina KourehliGarmendia IñakiSezgin ErsoyGarry KuanShaban A. A. MahmoudGary Li-Kai HsiaoShabana HabibGary StoreyShabbir Syed-AbdulGaspar RegoShabir ParahGaspare GiovincoShabnam Sadeghi EsfahlaniGastón MárquezShadab AlamGaurab DuttaShaddrack Yaw NusenuGaurav ChoudharyShadi AlzoubiGaurav MisraShady H. E. Abdel AleemGaurav VarshneyShady MagedGautami TripathiShafiqul HaiGautham PrasadShafiqul IslamGayathri RajagopalShafiullah KhanGee-Soo LeeShah NazirGeeta KasanaShahid HussainGeetesh K. MishraShahid IqbalGeetha SubbiahShahid TufailGefeson PachecoShahinur AlamGeir StorvikShahram HeydariGelu Ovidiu TirianShahram PayandehGemma María Gea GarcíaShahram SattarGen LiShahrokh HatefiGenaro OchoaShahzad AhmedGenda ChenShahzaib AhamadGeng-Bo WuShaik AkramGengyan ZhaoShaik Vaseem AkramGennaro VessioShailendra RajputGeno PeterShaina RazaGeorg FischerShakeel SoomroGeorg HähnerShaktivel ManavalanGeorge A. PapakostasShalei SongGeorge BelgiuShalendra KumarGeorge Cristian LazaroiuShalini DograGeorge Cunha CardosoShalli RaniGeorge D. ManolisShameem AhmadGeorge D. VerrosShamim Hasnat RiponGeorge DinosShamin SadrafshariGeorge FlorosShams A. M. IssaGeorge GrouiosShan JiaGeorge J. BesserisShan JiangGeorge KnopfShan Shan WangGeorge KyriacouShan ZhouGeorge MelillosShane SiebenalerGeorge ObaidoShangbin JiaoGeorge PantazisShang-Pin MaGeorge PapagiannopoulosShangzhi ChenGeorge R. KasyanShan-Hsiang ShenGeorge SuciuShan-Jen ChengGeorge ThéShanmugam ParivallalGeorge TsogkasShanmugan SengottainGeorge VargemezisShanmugavadivu PitchaiGeorge Z. ForristallShanshan HuGeorge-Othon GlentisShanshan LiGeorgi Gary RozenmanShanshan WangGeorgia KontogianniShanshi ZhouGeorgia KoukiouShanti K. SwarupGeorgia SakellariShanwu LiGeorgiana SimionShanyong ChenGeorgios BouloukakisShaobo HeGeorgios KavallieratosShaocheng LuoGeorgios LeontidisShaoguang HuangGeorgios ManikisShaohua WanGeorgios N. TsigaridasShao-I ChuGeorgios PanouShaoju WuGeorgios PapagiannisShaojun ZhuGeorgios PappasShao-Ku KaoGeorgios TsantopoulosShaopeng HuGeorgios TsaramirsisShao-Ping LuGeorgios TsigaridasShaoxiong SunGeorgios TsoumanisShaoyang LinGeorgios ViolakisShao-Yi HsiaGe-Peng JiSharad Chandra RajpootGerald Berger-WeberSharareh SharififarGeraldo Pereira Rocha FilhoSharat Chandra BarmanGerardo ArangurenSharmila GaikwadGerardo Arturo López MuñozSharnil PandyaGerardo FebresShashank GahlautGerardo FloresShashank PathakGerardo Gutiérrez-JuárezShaul OzeriGerardo Romero GalvánShaun AzzopardiGerardo Ulises Diaz-ArangoShaun WestGerasim Vladimirovich KrivovichevShawn ShengGerasimos RazisSheeba SujitGerasimos VonitsanosSheeraz ArifGerasopoulos DimitriosSheeraz IqbalGerd DobmannSheetal SonavaneGergely VakulyaShefa DawwdGerhard MüllerSheikh Ariful IslamGermain FaitySheikh Safiul IslamGerman CastilloShen Hin LimGermán GonzálezShen YanGhada KhoribaShen YinGhaith KhalilSheng ChengGhaleb HoblosSheng Cheng YehGhani-Ur RehmanSheng LiGhanshyam TejaniSheng TanGhareb HamadaSheng XuGhasem DerakhshanShengbo HuGhazal A. FahmyShengbo ShanGhazal RouhafzayShengbo YeGheorghe GavriloaiaShengchen LiGheorghe PaltaneaShengchuan WuGheorghe ParaoanuShengchun PiaoGheorghe SebestyenShengheng LiuGheorghe-Daniel VoineaShenghong HeGhizlane OrhanouShenghong LiGholam Hossein RoshaniShenghua CuiGholamhosein MoloudianSheng-Lih YehGhorban TaghizadehSheng-Lung PengGhulam AliShengping LvGhulam DastgeerSheng-Po ChangGhulam GilanieSheng-Sheng YuGhulam HafeezSheng-Shih WangGhulam MuhammadShengwen XuGi Pyo NamShengyuan LiGiacomo CabriShengzhou BaiGiacomo ColombattiShenhao WangGiacomo InnocentiSheraz AlamGiacomo MuntoniSheraz AslamGiacomo NavarraSheraz KhanGiacomo PeruzziSheridan MayoGiacomo ValleSherief HashimaGiacomo ZiniSherif AbdelhamidGiambattista GruossoSherif Ahmed ZaidGiampaolo VettaSheroz KhanGiampiero de CesareShervin HashemiGiampietro CasasantaShervin MinaeeGian Luca GiorgiShervin Rahimzadeh ArashlooGian Marco DumaSherzod TuraevGiancarla AlbertiShiang-Yi HanGiancarlo CondelloShibnath SamantaGiancarlo IannizzottoShibo ZhangGiancarlo OrengoShican QiuGiandomenico CarusoShichao DingGiandomenico SpezzanoShidan WangGianfranco AvitabileShieh-Kung HuangGian-Franco Dalla BettaShigeho TanakaGianfranco GagliardiShiguo HuangGianina DodiShihab JimaaGianluca CaparraShih-Hsun ChenGianluca CiardiShih-Hung YangGianluca Di FlumeriShih-Jeh WuGianluca EspositoShih-Jung WangGianluca GattiShih-Lin LinGianluca L. ZingariniShih-Meng HsuGianluca PalermoShiho KimGianluca ZazaShih-Wei SunGianluigi TiberiShih-Wen HsiaoGianmarco DinelliShijian WuGianni CerroShikha TripathiGianni StanoShilei LvGianpaolo CoroShilong SunGiansergio MenduniShilpa GiteGibin GeorgeShilpi AgrawalGiedre SabaliauskaiteShimaa A. Abdel Hakeem AbdelhakeemGiedrius JanusasShiming HeGilbert De MeyShin HurGilbert LimShin Jun ParkGilberto Anzueto-SánchezShin UtsuzawaGilberto CorsoShin′ichi WarisawaGilberto J. ArrudaShingo YamaguchiGilberto Pérez LechugaShinichi SobueGil-Jin JangShinichi YamagiwaGilles ParentShinji TamuraGilliard SilveiraShinpei OgawaGina AmbrosioShintaro SukegawaGiner Alor-HernándezShiqi ZhengGinés García-MateosShishir ShandilyaGinno MillánShital S. ChiddarwarGino DardanelliShiuhchuan HerGintautas BureikaShiv K. SharmaGinu RajanShiv Kumar LohanGiordano FranceschiniShivani SathishGiorgia AgrestiShivlal MewadaGiorgia FioriShiwei LiuGiorgia GiovanniniShiyang TangGiorgia ManfèShiyang YanGiorgio BiagettiShiyi TianGiorgio DavicoShiyuan LiuGiorgio FiccoShize HuangGiorgio FumeraShizhuang WengGiorgio MauroShmuel SpringerGiorgio PennazzaSho KojimaGiorgio TerracinaShoaib FarooqGiorgio VassenaShobit AgarwalGiorgos KountiosShofarul WustoniGiovanna CalòShogo MisuGiovanna CaparelloShogo OkamotoGiovanna ConcuShohreh GhorbaniGiovanna Maria DimitriShoji TominagaGiovanna Maria Gautier Di ConfiengoShokoufeh Monjezi KouchakGiovanna Maria SannaShou FengGiovanna OrsiniShoulin YinGiovanni AngiulliShouzhen ZengGiovanni ArtaleShovan BhaumikGiovanni CeccioShow-Shiow TzengGiovanni CollodiShoyebmohamad F. ShaikhGiovanni CrupiShreen El-SapaGiovanni Di DomenicoShreyas SrivatsaGiovanni DiracoShriyam ShauryaGiovanni FabbrocinoShruti KangaGiovanni GiganteShruti VyasGiovanni GugliandoloShu Ting GohGiovanni LeucciShuai LiGiovanni LucchettaShuai MaGiovanni MaccioniShuai WangGiovanni MauroShuai ZhouGiovanni MottolaShuaihang PanGiovanni PalleschiShuaijie WangGiovanni ParagliolaShuang YangGiovanni PecoraroShuangxi LiuGiovanni RalliShuangyi YanGiovanni SchmidShubham SharmaGiovanni VinettiShubhangi ShuklaGiovanni VitaleShubhra JainGiraudo AlessandroShu-Chen HsiehGi-Ren LiuShuchi UpadhyayGisbert RiessShuchuang DongGiseop NohShudong YuGiulia BaldiniShu-Fen TuGiulia ButtazzoniShuhui GongGiulia GerminarioShu-Hui LiuGiulia MoroShuichi ObayashiGiulia SaccoShuiliang FangGiuliana BilottaShuisheng ZhouGiuliana RamellaShuitao GuGiuliana VitielloShuja AnsariGiuliano GrossiShujie YangGiuliano VitaliShuke HuangGiuliano ZanchettaShukra Raj PaudelGiulio ArcangeliShuli FanGiulio CaldarelliShun Hsyung ChangGiulio D′EmiliaShun HuGiulio M. BiancoShun-Chi WuGiulio RosatiShun-Fa HwangGiulio VialettoShunichi HattoriGiuseppe AndreoniShunping ZhangGiuseppe ArrabitoShun-qing RenGiuseppe ArteseShunsuke NagahamaGiuseppe BrunettiShuo LiGiuseppe CampobelloShuo YangGiuseppe CastaldiShuo ZhaoGiuseppe CattaneoShuo-Han ChenGiuseppe ChitarinShuo-Tsung ChenGiuseppe CovielloShuqiang WangGiuseppe Del CoreShuqing ZhangGiuseppe Di LeoShuting WanGiuseppe Di MassaShuvodeep DeGiuseppe Francesco Cesare LamaShuwen ZengGiuseppe LacidognaShuwen ZhangGiuseppe Marco TinaShuxiang GuoGiuseppe Maria Luigi SarneShuxiao LiGiuseppe Maria PaternòShu-Yi LiawGiuseppe MinerviniShuzeng ZhangGiuseppe MussumeciShuzheng WangGiuseppe NittiShu-Zhi LiuGiuseppe PalestraShweta J. MalodeGiuseppe PelosiShyi-Chy ChengGiuseppe PetrilloShynu Padinjappurathu GopalanGiuseppe PoleseSibao WangGiuseppe RiccioSibo LiGiuseppe RuvioSichen YuanGiuseppe SancataldoSicheng ZhaoGiuseppe Schirripa SpagnoloSicong ShaoGiuseppe ScottiSiddhartha BhattacharyaGiuseppe SimoneSiddhartha Bikram PandayGiuseppe VaroneSidheswar RoutrayGiuseppina GiniSidike PahedingGiuseppina PappalardoSidnei Alves De AraujoGlauco FontgallandSidnei PaciornikGlauco PedrosaSidney GoossensGleb YurkovSie Long KekGleifer Vaz AlvesSiegfried ReichGleiston DiasSifat MomenGlen CooperSigurd EskelandGlen SegellSigurður BrynjólfssonGlenn Van SteenkisteSihan HuangGloria BeraldoSihao ZhaoGloria Cerasela CrisanSi-ho ChaGnanou Florence SudhaSihua ShaoGo YamakoSihyung LeeGobinath JegannathanSiim HeinsaluGodfrey MutowoSijo MathewGokhan OzturkSijun DongGomaa A. M. AliSijung HuGonçalo DiasSikai ZhaoGoncalo JesusSikandar AminGonçalo MarquesSikandar KhanGong XiangSikha BaguiGongjian ZhouSilje Ekroll JahrenGongming ZhaoSilvan RagettliGongping HuangSilvana AlborésGong-Xu LiuSilvana FaisGonzalo De MiguelSilvano DragonieriGonzalo Sanz-Diez De Ulzurrun CasalsSilverio García-CortésGoorak KwonSilvestro RoattaGopal KrishanSilvia BiasottiGopal Ramdas MahajanSilvia CarraGopal TadepalliSilvia CorchsGopinath SamantaSilvia de Miguel-BilbaoGoran B. MarkovicSilvia GaftandzhievaGoran KvaščevSilvia KrugGoran PoparićSilvia LogozzoGoran S. RistićSilvia LoriGoran T. DjordjevicSilvia Martínez SatorresGorana BaršićSilvia MironeasaGorazd BombekSilvia OrtínGordan GledecSilvia RizzatoGordana JakovljevićSilvia Soria HuguetGórnicki KrzysztofSilvia ZiegerGosteva AlevtinaSilvio LorenzettiGou KoutakiSilvio QuincozesGour Mohan DasSilvio SciortinoGovind K. MakhariaSilvio SimaniGovind VashishthaSilviu ButnariuGovindan Suresh KumarSima NoghanianGovindarajan MuraliSimas RackauskasGovindasamy ManiSimon AnnaheimGovindasamy NarayananSimon J. BleikerGowtham MunirajuSimon KolmaničGraham BrookerSimon VrhovecGrazia Giuseppina PolitanoSimon WangGrazia IadarolaSimona Di MeoGrazia Lo SciutoSimona MiclausGraziella ScandurraSimona MirelGreg DrogeSimona MoldovanuGreg J. TallentsSimona PetrakievaGreg W. BishopSimona RamanauskaitėGrégoire CattanSimona ViolinoGregor BanoSimonas RamanavičiusGregor DonajSimone CavaleraGregor GersakSimone CiampiGregor HarihSimone DonadelloGregor StrleSimone GrassiGregorio AndriaSimone PorcuGregory GrigoropoulosSimone Quondam-AntonioGregory KowalskiSimonetta CaponeGregory LewisSimranjit SinghGregory P. MeyerSina Shaffiee HaghshenasGregory ProvanSinan Melih NigdeliGrigor Y. MihaylovSinan Q. SalihGrigoriy Isaevitch GreisukhSin-Chi KuokGrigory Ivanovich DolgikhSingaravelu RamalingamGrzegorz BartoszSiniša HusnjakGrzegorz BlakiewiczSiniša Mastelić IvićGrzegorz BorowikSiniša RandjićGrzegorz FilcekSin-Jin LinGrzegorz FiloSitakanta SatapathyGrzegorz GrunwaldSiti Salasiah MokriGrzegorz Henryk KopeckiSitthichok ChaichuleeGrzegorz IlewiczSiu Shing ManGrzegorz JanickiSivakandan ManiGrzegorz JasinskiSivaramapanicker SreejithGrzegorz KarońSizhen BianGrzegorz KoniecznySkibicki JacekGrzegorz KrzanSlah YaacoubiGrzegorz MieczkowskiSlavcho BozhkovGrzegorz PsujSlavko RakicGrzegorz SiekluckiSlavko RupčićGrzegorz SobczakSławomir CięszczykGrzegorz ŚwirniakSławomir CzarneckiGrzegorz TytkoSławomir JakiełaGrzegorz UlachaSlawomir JudekGrzegorz WiczyńskiSławomir KujawskiGrzegorz ZającSlawomir SujeckiGrzegorz ZielińskiSlawomir WiakGrzybek DariuszSlim NaifarGu Eon KangSlobodan BabicGuan GuiSlobodan DudićGuanci YangSlobodan RadojevicGuang LiSmaranika NayakGuangda ChenSmith Kashiram KhareGuangfu WuSnehalatha UmapathyGuanghao SunSnježana Firšt RogaleGuanghua SunSnježana Rimac-DrljeGuanghui RenSobia BaigGuanglei WangSocrates BasbasGuangli DaiSofia AgostinelliGuangliang LiSofia PillaiGuangtao ZanSofia SuvorovaGuangwei HuangSofiane BououdenGuangwei YangSofiane KarouiGuangwu FangSofiene MansouriGuangyan HuangSofya P. KulikovaGuangyang FangSofyan A. TayaGuangyou FangSohaib LarabaGuangyuan LiSohail MumtazGuangyuan ZhaoSohgawa MasayukiGuanqiu QiSoi-Hoi Michael LamGuanyi MaSokratis KatsikasGuanying HuoSolehuddin ShuibGuarino AlfonsoSolmaz Maleki DizajGuennadi SaikoSomayeh B. ShafieiGuenter ReissSomayyeh AsgariGueorgui GueorguievSomayyeh KoohiGuichao YangSomdip DeyGuichen LiSomsubhra ChakrabortyGuido BelforteSonain JamilGuido BorghiSong GaoGuido MarsegliaSong JiangGuido PanzarasaSong LiGuido PerroneSong LuGuido RubinoSong YangGuido ToselloSongdong ShaoGuijin WangSonghua YanGuilherme B. LucasSongsong LiuGuilherme Lucio Abelha MotaSongyuan ZhangGuilherme Luiz MoritzSonia BahraniGuillaume DufourSonia FreddiGuillaume PolidoriSónia Matilde Fonseca MateusGuillermo Asín-PrietoSoo Fun TanGuillermo AzuaraSoochang ParkGuillermo Cámara-ChávezSoohwan KimGuillermo Fernando RegodónSoo-Hyung KimGuillermo IglesiasSooman LimGuillermo Lopez-ReyesSoonchul KwonGuillermo M. ÁlamoSoon-Jae KweonGuillermo PlazaSoon-Jong JeongGuillermo Rey GozaloSophie ArmaniniGuillermo Rodriguez AbitiaSorin CiortanGuillermo Royo CalderónSorin HinteaGuillermo Valencia-PalomoSorin Ioan DeaconuGuillet ValérySorin MunteanGuiping RenSorin ZoicanGuisheng XuSoslan A. KhubezhovGuiwei LiSotirios ApostolakisGuiying HeSotirios DiamantasGul RahmanSotirios SpantideasGulistan RajaSotiris AvgoustiGulshan VermaSoufiene Ben OthmanGulyas AndrasSouhir ZghalGunhee HanSoumendu SinhaGunnar RittSoumya BanerjeeGunter HagenSourabh SolankiGuobiao HuSourav BanerjeeGuochen WangSoyang KwonGuodong ZhouSoyeon LeeGuogang ZhangSpandan RoyGuoliang ShangSpanu AndreaGuo-Lun LuoSparsh SharmaGuomei ZhangŠpela ČučkoGuoniu ZhuSpilios GiannoulisGuoqi QianSpiridoula V. MargaritiGuoqiang LiuSpyridon KintziosGuoqiang ZengSrdjan BrkićGuoqing ZhangSrdjan ZuskinGuoqing ZhaoSrecko StopicGuosheng HuangSreenu SreekumarGuo-Shiang LinSreeprasad SreenivasanGuowen YaoSrete NikolovskiGuoXia XuSri Arttini Dwi PrasetyowatiGuoxu LiuSridhar ArjunanGuozhong XingSridhar ChandrasekaranGupta SandeepSrijana AcharyaGuru PrakashSrikanth ItapuGurumadaiah Ajay KumarSrinivas KolluruGururaj Kudur JayaprakashSrinivas KoppuGustavo De Novaes Pires LeiteStamatios AmanatiadisGustavo De Oliveira AlmeidaStamatios GiannoulakisGustavo PérezStan ZurekGustavo PintoStanislav Grigoryevich DolgikhGuy BurroughesStanislav HarizanovGuzek MarekStanislav LenartGwan-Hui LeeStanislav MarchevskýGwenn EnglebienneStanislav OndasGwo-Jiun HorngStanislav PopelkaGyeongsik YangStanislav ZvánovecGyorgy JonaStanisław BaciorGyörgy KovácsStanisław DrożdżGyörgy SzabadosStanisław DuerGyörgy WersényiStanislaw FlagaGyudo LeeStanislaw GallaGyula GrófStanisław HożyńGyula MesterStanislaw LegutkoGyusun HwangStanisław MazurHa Duong NgoStanislaw SzwajaHabib HamamStanisława KoronkiewiczHabib KraiemStathis C. StirosHabibollah HaronStavros KoubiasHabib-ur-Rehman AtharStavros SouravlasHadeed SherStef VandermeerenHadeel AlazzamŞtefan BodiHadi FarhadiStefan BrebanHadi HadshemzadehStefan FischerHadi KordestaniStefan K. EhrlichHadiseh NasariŠtefan KohekHadush Kidane MeresaStefan KrauterHaechul ChoiStefan Marian FrentHaewon ByeonStefan MarschnigHafiz Abd Ul MuqeetStefan PosladHafiz Imran Ahmad QaziStefan SchusterHafiz Muhammad Salman AjmalStefan WeiglHafiz Munsub AliStefania RomeoHafiz Tayyab RaufStefano BellucciHagit MesserStefano ColafranceschiHai DongStefano Di PaoloHai HuangStefano FaralliHai LiuStefano LauretiHai Van PhamStefano LegnaioliHai WangStefano LenciHai YuStefano LettieriHaibin CaiStefano MangioneHaibin SunStefano MauroHaibin WangStefano MotturaHaibin ZhangStefano PalermiHaibo ZhangStefano ParrinoHaibo ZhouStefano PastorelliHaider Al-KhateebStefano QuerHaidi IbrahimStefano RamatHaigang WangStefano RossiHai-Han LuStefano SalvatoriHaijun PengSteffen DerlienHailiang ChenSteffen GrautoffHailiang WangSteffen MoritzHailiang ZhangSteffen ReichelHailu XuŠtefica MrveljHailu YangStefka AtanassovaHaim MazarStela GeorgievaHaiming GanStelian BradHaipeng LiuStelian LupuHairong WangStelios ZimerasHaitao HuStella MarkantonatouHaitao LiStepan GorgutsaHaitao YuStephan BodenHaitao ZhengStephan HavemannHaixing LiuStephan PaulHaiyan ZhaoStephan StreuberHaiyang LiuStephan van GasseltHaiyang MaoStephan WarnatHaiying MaStephane BarlandHaiyun LiStéphane ManciniHakan KuntmanStephane RioualHakan OztopStephanie SahuguedeHakeem O. AyindeStephen CainHak-Seon KimStephen CzarnuchHala MostafaStephen D. ArcherHalil İbrahim CeylanStephen David WorrallHalina TarasiukStephen Gbenga FashotoHalit S. TurkmenStephen HollerHamad SyedStephen Mayowa FamurewaHamada EsmaielStephen OjoHamadjam AbboubakarStephen U. EgarievweHamdy SolimanStephen UgwuanyiHamed GholamiStephen WeisHamed Hashemi-DezakiStephen YoungHamed KhodadadiSteponas AšmontasHamed MalekmohammadiSteve ChanHamed MoayyedSteve CutchinHamed Mohebbi-KalkhoranSteve GaoHamed Nabizadeh RafsanjaniSteve MajerusHamed NosratiSteve MannHamed SaghaeiSteven ChattertonHamed YazdanpanahSteven G. SchauerHamid AL-AsadiSteven Solethu NkosiHamid Ali Al-AsadiSteven WagnerHamid MoghaddasiStević ZoranHamid MukhtarStewart McWilliamHamid RazmjooeiStoean RuxandraHamida HallilStrahinja DosenHamidreza HeidariStuart AndersonHamidreza KhaleghzadehStylianos E. TrevlakisHamidreza TaghvaeeSu Man NamHammad AfzalSuaib AkhterHammad AlotaibiSuan LeeHammoudi AbderazekSubash DahalHamoon ZohdiSubba Rao CheekatlaHamza BoukabacheSubhan KhanHamza FahimSubhayan MukherjeeHamza GabrielSubhi R. M. ZeebareeHamza Mohammed Ridha Al-KhafajiSubhradeep PalHamzeh BardaweelSubir MajumderHan Eol LeeSubramanian MuthaiahHan LiSubrata GhoshHan XiongSubrata NandyHan ZhangSuchinder K. SharmaHana CharvátováSudeep TanwarHana PacaiovaSudeep ThakuriHanaa SalemSudheer Kumar BattulaHanan A. Hosni MahmoudSudhir K. SinghHanbai ParkSudip PoddarHancheng LuSue LordHanchi ChenSufia RehmanHancong WuSufyan Ali MemonHanfei MeiSufyan GaroushiHang GaoSugato HajraHang Nga MaiSugeng SupriadiHang QuSugwon HongHang SuSuhail M. OdehHang YiSuhyun ParkHanjui ChangSujala ShettyHank WhiteSujan ShresthaHan-Kyu ChoiSujan YenugantiHanlin TanSujatha RamaniHanlin YeSujeong LimHanna BandarenkaSujin BureeratHanna JärveläinenSujit Kumar MandalHanna PazniakSujit PardeshiHanns de la Fuente-MellaSukanta Kumer ShillHanqi ZhuangSukanya RamarajHans Georg FeichtingerSukhveer SinghHans Peter LangSukjin LeeHans-Peter BischofSukrith DevHan-Wei ChangSulaiman Mohammed IbrahimHanwei ZhaoSuleiman Aliyu BabaleHanxin ChenSuleiman YerimaHany AtlamSüleyman EkenHan-You JeongSuliman MohammedHan-Yu LinSu-Ling YehHanzhong WuSultan Daud KhanHao ChenSultan S. AlshamraniHao CuiSultan ShoaibHao GuoSumaiya ThaseenHao LiSuman DuttaHao LuSumana BiswasHao ShenSumant P. RadhoeHao SongSumanta SahooHao WuSumedha MoharanaHao XiongSumer SinghWalHao YanSumeth YuenyongHao YuSumit KumarHao ZhangSumit KushwahaHao ZhuSumita GoswamiHaochong HuangSumita MishraHaojun ZhangSun Young KimHaoqi SunSundara Tejaswi DigumartiHaoqian HuangSundaram GopikrishnanHaoqing LiSundaravaradan Navalpakkam AnanthanHao-Qing YangSunday EkpoHaoran QuSung Min ParkHaoran WeiSung ParkHaoran WenSung Ryul ShimHao-Tian WuSung Wook BaikHao-Ting LinSungchul LeeHaotong CaoSungchul MunHao-Wen DongSung-Hak LeeHaowen XuSunghan KimHaowen YanSungho KimHaoxin WangSungho LeeHaoyang CuiSungho SuhHaoyi MaSunghoon ParkHaq NawazSunghwan KimHarald KlimachSungjun KimHarco Leslie Hendric Spits WarnarsSungjun YooHardik Jeetendra PandyaSung-Jung HsiaoHaret RosuSung-Liang ChenHari Kishan KondaveetiSungmin HanHari Shankar SinghSung-Min KimHarihara GopalanSungmook JungHariharan LalgudiSungtek KahngHariharan MuthusamySung-Won ChoHarikumar RajaguruSungwon KimHariprasath ManoharanSungwook ChoHarish ChanderSungwook PaekHarish KumarSungyong ChoiHarjit SinghSun-Hong MinHarkaitz EguiraunSunil GuptaHarleen KaurSunil KaramchandaniHarold KimSunil Kishore ChakrapaniHaroldo MarquesSunil Kumar SharmaHaroldo Takashi HattoriSunil Kumar SukumaranHaroon AmanSunipa RoyHarri HakulaSunita DhavaleHarry SeptantoSunita KumbhatHarshani WijerathneSuniti SuparpHartmut BarteltSun-Je KimHartmut WitteSunjung KimHasan Abdullah Hasan NajiSunwook KimHasan F. AlesarySuparerk JanjarasjittHasi Rani BaraiSuparshya Babu SukhavasiHasmat MalikSupriyo RayHassan AbuhassnaSuraj AmatyaHassan AlamiSuraj PawarHassan AlgadiSuraj Prakash SahooHassan El AlamiSurajit KunduHassan El-RamadySuram Singh VermaHassan Haes AlhelouSuraparb KeawsawasvongHassan QjidaaSurasak KasetsirikulHassan RabahSurender Reddy SalkutiHassan SarmadiSurendiran BalasubramanianHassane HotaitSurendra Krushna ShindeHassanein AmerSuresh Kumar KailasaHassen M. OuakadSuresh MeruguHayato SatoSuresh NeethirajanHayder AlmosaSuresh NuthalapatiHayder Al-ShukaSuresh VermaHayder I. MohammedSurjeet DalalHayley Treloar PadovanoSusan RobertsonHayrettin KöymenSusana CatarinoHayriye GidikSusana CostaHaytham MiligiSusana de MarcosHazem El-AlfySusana Hornillo MelladoHazim ElbakrySusana NovaisHe RuiSusana Ortega CisnerosHeba AhmedSusanna SpinsanteHector Eduardo RomanSusanna SummaHéctor García-MartínezSusanne Taina Ramalho MacielHéctor Guillen BonillaSusanta MahatoHector KaschelSushank ChaudharyHéctor Manuel Pérez-MeanaSushil KumarHéctor MigallónSushil Kumar SinghHector PomaresSusilawati SusilawatiHéctor RotsteinSusrutha Babu SukhavasiHéctor Vega CarrilloSutanu BhowmickHee-Cheol KimSuvash Chandra PaulHeechul JungSuyel NamasudraHeechul YoonSu-Yen ChenHee-Dong KimSuyu LiHee-Jo LeeSuyun WangHeejun RohSveinung SægrovHeena RathoreSven G. BilénHeeseok OhSven GotovacHeeso NohSvetislav SavovicHeewon JungSvetlana BičárováHeeyoul KimSvetlana BoudkoHegazy RezkSvetlana KrasnovaHehao NiuSvetlana V. PolevovaHeike ArgstatterSvetlana Von GratowskiHeike KahlertSwagato SarkarHeikki AilistoSwakkhar ShatabdaHein VenterSwaminath VenkateswaranHeinrich RuserSwaminathan RamabadranHeinz BurtscherSwapan TalukdarHeinz-Dietrich WuttkeSwarniv ChandraHelbert Eduardo EspitiaSwarup BiswasHélder FonsecaSwathi IppiliHelena GripSwati SachanHelena NavasSwetava GanguliHelena TeteryczSyed Abdul Rehman KhanHélène DebedaSyed Ahmad Chan BukhariHe-Liang HuangSyed Ali Abbas KazmiHeling WangSyed Ali HussainHélio Luiz SimonettiSyed Basit Ali BukhariHelmut Karl LacknerSyed Furqan QadriHemendra KumarSyed Khairul BasharHendrik GronauSyed MasoodHendrik RotheSyed Mohammad Hassan ZaidiHendry HendrySyed Muhammad ImranHeng WangSyed Saad Azher AliHenk KoppelaarSyed Sabir Hussain BukhariHenri Vähä-YpyäSyed Sajid UllahHenrik SchiolerSyed Tahir Hussain RizviHenrik ZsiborácsSyed Thouheed AhmedHenrique SilvaSyed Turab RazaHenry CarvajalSyeda Iffat NaqviHenry InegbedionSyh-Yuan TanHenryk BednarskiSylvain FeruglioHenryk KaniaSylvain MagneHenryk KasprzakSylvie HermouetHenryk KrawczykSylvie PourchetHenryk PalusSylvio Barbon JuniorHenryk RatajkiewiczSylwester KłyszHeping ShuSylwia AndruszczakHerbert Michael HeiseSylwia BalutaHeri NurdiyantoSylwia GwoździewiczHerman C. MyburghSylwia Werbińska-WojciechowskaHerman L. OfferhausSylwia WłodarczakHerman Van WerkhovenSyoji KobashiHermann GillySyuichi ItahashiHerminio Martínez-GarcíaSzabó Ferenc JánosHernán Gonzalo-OrdenSzabolcs FischerHernâni Miguel Reis LopesSze Shin LowHessam GolmohamadiSzeidert IosifHeung Soo KimSzidónia LefkovitsHeung-No LeeSzilvia NagyHeyang (Thomas) LiSzi-Wen ChenHeydar Toossian ShandizSzonya DurantHezhen LouSzu-Yu KuoHichem MrabetSzymon ChmielewskiHidekazu NishimuraSzymon MalinowskiHideki NaitoSzymon SiecińskiHidenori SuganoSzymon WojciechowskiHideyuki HasegawaTabitha RothHideyuki KanematsuTad GonsalvesHieu NguyenTadashi ItoHiginio Rubio AlonsoTadatsugu MorimotoHilario Gómez MorenoTadeusz KwaterHilman PardedeTadeusz MikolajczykHimadri Nath SahaTadeusz MoskalikHimal A. SuraweeraTadeusz PisarkiewiczHimanshu MittalTadeusz SalacinskiHimanshu SharmaTadeusz SondejHiram CalvoTadeusz StacewiczHiram Galeana ZapiénTadeusz StepinskiHirayuki EnomotoTadeusz WszołekHiroaki KikuchiTaekeun OhHiroaki KuzeTaek-Kun NamHiroaki MaedaTaha A. ElwiHiroaki YokotaTaha BakhshpooriHirokazu MadokoroTaher S. HassanHiroki ChoTahereh EftekhariHiroki IshibashiTahesin Samira DelwarHiroki IshizukaTahir AlyasHiroki NishikawaTahir HussainHiroki TamuraTahir MahmoodHiromi KurokawaTahir MaqsoodHiroo KunimoriTahseen Al-wattarHiroshi ChureiTahsin ShoalaHiroshi FuketaTaihong LiuHiroshi MatsuoTai-Jin SongHiroshi SasamotoTaikyeong Ted JeongHiroshi TanakaTairan LiuHiroshi TsunodaTairong KuangHiroshi UchidaTaisiya KozlovaHiroyuki SatoTaiyong LiHiroyuki WakiguchiTaj Muhammad KhanHisashi SatohTakanori TaninoHisham ArafatTakashi HikageHla Myo TunTakashi MatsumaeHo NamgungTakashi YamauchiHo Pham Huy AnhTakashiro TsukamotoHoang D. LeTakaya YuizonoHoang NguyenTakayasu MishimaHoang Van XiemTakehito KikuchiHoang-Yang LuTakeshi FujinoHockguan GohTakeshi KurataHoda SarparastTak-Shing ChingHoda ZamaniTakuhiro OtosuHodam KimTakumi AsakuraHoe Joon KimTakumi MitsuhashiHoh Peter InTakuo SakonHoi-Shun Antony LuiTakuya SasataniHojjatollah EsmaeiliTakuya TaniguchiHolger HillTakuya TsukagoshiHolger MauneTakuya TsutsumiHolly HollingsworthTalgat R. GazizovHolly Sullivan-TooleTalha Mahboob AlamHonarvar Shakibaei AsliTallha AkramHong DingTam Q. NguyenHong GuoTamal RoyHong LiTamara BasovaHong LiuTamara JurinaHong PengTamara RadivilovaHong TangTamara ŠkorićHong WangTamara TomašegovićHong XieTamara V. TulaikovaHong ZhangTamás D. NagyHong ZhaoTamás DócziHong ZhengTamás HaideggerHongbao XinTamás OroszHongbin LinTamas PflanznerHongbin MaTamás RuppertHongbin SunTamer KhatibHongbin YuTamer ShamseldinHongbing ChenTamir ShaqarinHongbo MuTan Phan XuanHongbo WangTang Song NienHongbo ZhangTangao HuHongchang LiTangirala Venkata Krishna KarthikHongchen MiaoTanja ZidaričHongfeng YuTan-Jan HoHonghao GaoTanmay BhowmikHonghui HeTanmay KulkarniHongji XuTanmaya Kumar SahuHongjie HuTanmoy MajumderHongjie JiangTanmoy ShankarHonglei XuTanveer AhmadHongli HuTanveer FaridHongliang HeTanveer HussainHongliang LiTanveer ZiaHongmin WuTanweer AlamHongming LyuTanweer AliHongming XuTanya HutterHong-Ning DaiTanzilur RahmanHongping HuTao GengHongtian ChenTao HuangHongwei HuangTao JiangHongwei MeiTao LiHongwei QuTao LiangHongwu WangTao LiuHongxiang WeiTao SongHongxing ZhangTao WangHongyan ZuoTao YanHongyang MaTao YangHongyin ShiTao YinHongying LiuTao YuanHong-Youl HaTao YueHongyu WangTao ZhangHongyu ZhaoTao ZhaoHonor PowrieTao-Hsing ChenHooman EsfandiariTapan SenapatiHoracio Legal AyalaTapio A. HeikkiläHoracio PaggiTara L. McIsaacHorng-Horng LinTarakeswar ShawHorn-jiunn SheenTarek DjerafiHosein Mohammadi MakraniTarek GaberHosein NaderpourTarek Saleh AmerHossam DonyaTareq AssafHossam KotbTareq Hasan KhanHossam SelimTarik RashidHossein HassaniTariq Abed MohammedHossein KabirTariq AliHossein KarimpourTariq AzizHossein MardaniTariq UmerHossein MoayediTarnita DanielaHossein ShahinzadehTaro UedaHossein TaheriTasadduq ImamHotaka TakizawaTaseer MuhammadHou-bin LiTa-Shun ChuHoufang LiuTat Thang Vo-DoanHoussem JerbiTata Sanjay Kanna SharmaHovannes KulhandjianTatevik ChalyanHoward MatisTatiana JaworskaHo-Youl JungTatiana PetrovaHoyoung KimTatsat PatelHristos AnastassiuTatsuaki KanaiHrvoje GlavasTatsuhito HasegawaHrvoje KalinićTatsuya IwataHrvoje KarninčićTatu RojalinHsiang-Chen WangTatyana KrupnovaHsiang-Chieh ChenTaufiquar KhanHsiao-Chung LinTauheed Khan MohdHsiao-Lung ChanTawheed HashemHsiao-Ting TsengTawsifur RahmanHsieh-Fu TsaiTayab MemonHsien-Kuo ChangTaye Girma DebeleeHsien-Tsung ChangTayfun TanbayHsin HsiuTaylor ChomiakHsin-Jang ShiehTecla BonciHsin-Ta ChiaoTeddy CraciunescuHsin-Wei HsuTeeranoot ChanthasopeephanHsin-yi WenTeguh HerlambangHsiu-Ming WuTeh-Lu LiaoHsiu-Wen ChangTein-Yaw ChungHsuanling KaoTejasvi ParupudiHsueh-Chun LinTe-Jen SuHu DingTeng LiHua LiTengfei XuHua LiuTengfei ZhengHua ShiTeo Tee HuiHua SunTeodor IlievHua YanTeodorico CaporasoHua YuTeodoro MontanaroHua ZhangTeodor-Viorel ChelaruHua ZouTeresa CarvajalHuabiao ZhangTeresa Maria Pinto Coelho Amado VasconcelosHuabin WangTeresa PamułaHuachao MaoTeresa ZawadzkaHuadan ZhengTerézia PošivákováHuafeng LiuTero TuovinenHuai ZhengTerry LichtorHuailong ShiTeruo OnishiHuaizhe YuTesfalem WelearegayHuakang LiangTessai HayamaHuaming ChenTetsuhiko F. TeshimaHuaming WuTetsuo YamaguchiHuan ZhouTetsuya AkitsuHuang WeiguoTetsuya HiraishiHuang-Hsing PanTetsuya IshikawaHuansi HuTetsuya KaiHuanyu ChengThabet KacemHuan-Yu ZhaoThanapon NorasetHuaping WangThanh Duc DangHua-Yi HsuThanh-Canh HuynhHubertus HimmerichThanh-Phong DaoHuck C.-H. YangThanh-Phong PhamHudyjaya Siswoyo JoThanh-Tuan NguyenHugo AbundisThanh-Van HoangHugo AguasThanigainathan PrakashHugo Celso Dutra De SouzaThashmee KarunaratneHugo F. MartinsThatiane Lopes Valentim Di Paschoale OstolinHugo F. Posada-QuinteroTheam Foo NgHugo FerreiraThembelihle DlaminiHugo Gonçalo OliveiraTheng Choon OoiHugo José Nogueira Pedroza Dias MelloTheodore GanetsosHugo Leiria NevesTheodore KotsilierisHugo LouroTheodore P. PachidisHugo R. W. TouwTheodoros KarakasidisHugo Rojas-ChávezTheodoros LaopoulosHugo S. OliveiraTheodoros N. KapetanakisHugo SobralTheodoros SemertzidisHugo Valadares SiqueiraTheofanis OrphanoudakisHugo Yañez-BadilloTheofilos PapadopoulosHuh Tae MyungTheyazn AldhyaniHui HuangThiago BazzoHui LiThiago OliveiraHui LiuThibault LaplaceHui PangThibault NapoléonHui XiongThierry BoschHui YanThierry MoulinHui YangThierry RibeiroHui ZhangThierry VillemurHui ZhaoThies LüdtkeHuicheng LaiThi-Kien DaoHuidong LiThilo PfauHui-Hsin HsiaoThippa Reddy GadekalluHuihua Kenny ChiangThirunavukarasu RamkumarHuiling ChenThirupathi RavulaHuimin ZhaoThi-Thu-Huong LeHuiping ZhouThittaporn GanokratanaaHülya Gökalp ClarkeThokozani ShongweHuma ZiaThomas C. HutchensHumaira NisarThomas CilloniHumberto LagunaThomas ClausenHumberto Lobato-MoralesThomas E. DickHumberto Martinez BarberaThomas ErikssonHumberto VerdejoThomas FarrellHung CaoThomas GreinerHung-Chi ChuThomas HerlitziusHung-Fu ChangThomas J. BaumgartenHung-Yin LinThomas KissingerHung-Yu ChienThomas KoutsosHung-Yu WangThomas KraussHuong Nguyen ThuThomas KusserowHuong-Giang DoanThomas McKenzieHurevich MattanThomas P. KarnowskiHurriyet YilmazThomas PoratheHusam A. FoudehThomas RobertazziHusham Farouk IsmailThomas RuhtzHussain Al-RizzoThomas StieglitzHussain AltammarThomas UchidaHussein AttiaThomas VasileiadisHussein Hasan AlRikabi AliThomas Voglhuber-BrunnmaierHuthaifa ObeidatThomas W. F. MittlmeierHuu-Tuan NguyenThomas WendtHuy Trung NguyenThorsten LuxHwang-Cheng WangThrishantha NanayakkaraHwansoo LeeThye Foo ChooHyam Nazmy Bader KhalafTiago Henrique MachadoHyemin KimTiago J. RatoHyeokhyen KwonTiago Jonatan GirelliHyeongtae AhnTiago Manuel Ribeiro GomesHyeon-June KimTiago Rodrigues TavaresHyeonseok KimTian WangHyeran NohTian ZhaoHyo Jong LeeTian-Bing XuHyogon KimTiancheng LiHyokeun LeeTiancong HuangHyong Doo JangTianfei ZhouHyoukryeol ChoiTianhan LiuHyoung-Kyu SongTianhao YuHyowon BanTianju SuiHyun KwonTianlong LiHyun Sung KimTianlong ZhangHyunbum KimTianou ZhangHyunchae ChunTianqi SongHyunchol ShinTianqi YuHyung D. BaeTianshi WangHyungil ChaeTianshuang WangHyung-Jin MunTianwei XingHyungjoo KimTianxing LiHyung-Joon SeoTianyi JiaHyung-Tae KimTianyiyi HeHyungwoo KimTianyue ZhangHyun-Ha ParkTianyun ZhangHyunhee ParkTiberiu LetiaHyunseok SeoTibor GuzsvineczHyunsoo LeeTibor HorakHyunsu LimTibor IžákHyunwoo LeeTibor KrenickyHyun-Woung KwonTibor SzkaliczkiI. Jénnifer GómezTibor TajtiIacopo TamellinTiebing LuIakov LyashenkoTiedong ZhangIan ChurchTie-Jun WangIan McAndrewTieling ZhangIan PapautskyTien Dat NguyenIan SharpTien NguyenIan WilliamsTien Yin ChouÍbis A. P. MoraesTien-Lung SunIbraheem Kasim IbraheemTien-Ying KuoIbrahim A. ElgendyTihomir DovramadjievIbrahim AlsohaimiTilen BreceljIbrahim B. M. TahaTill SieberthIbrahim MahariqTim HulsenIbrahim SadiekTim VanbellingenIbrahim ShaabanTim W. C. BrownIbrahim ZeidTim W. NattkemperIbrar AhmadTim WehnerIbukun Gabriel AwolusiTim ZiemerIchiro FujitaTimo LauteslagerIchiro YamadaTimo LipkaIclia Villordo JimenezTimo RantalainenIda E. TyschenkoTimofey ShevgunovIdawaty AhmadTimothy AkpenpuunIdilia BatchkovaTimothy ClaypoleIdris YazganTimothy HustonIeva MisiunaiteTimur KarimovIevgen ZaitsevTimur N. MustafinIevgeniia KuzminykhTimur Sh. AtabaevIftikhar AbbasovTin LukićIgnacio Díaz-BlancoTina SabelIgnacio Enrique Zaldívar-HuertaTina TomazicIgnacio LijarcioTina VrabecIgnasi CorbellaTing BiIgnazio LicataTing GeIgo PaulinoTing HuIgor BelyaevTing JinIgor BuzalewiczTing On ChanIgor FilanovskyTing ZhangIgor GisterekTinghai ChengIgor GolyakTinghao ChenIgor GruicTingkui MuIgor MeglinskiTingting ZhangIgor S. LitvinchevTirthankar GhoshIgor TomicicTitu SamsonIgor UrmanovTitus SanduIgor VelkavrhTiziana MargariaIgor ZubryckiTiziana PietrangeloIhn-han BaeTiziana PolichettiIhor ShchurTiziano ZarraIhsan AliToader VictorinIhsan Ullah KhanToan DinhI-Hsi KaoToan Thanh DaoIjaz AhmadToan-Van NguyenIjaz GulTobias BraunIker Muñoz PérezTobias FeiglIker Pastor LópezTobias HabisreutherIkhlas Abdel-QaderTobias HemselIkram Ud DinTobias LappanIl Dong YunTobias MeisenIl Song HanTobias MeuserIlan ShimshoniTo-Cheng WangIlaria BortoneTodd T. SchlegelIlaria CacciariTohru SasakiIlaria De MunariTohru YamadaIlaria MatacenaTolga YükselIlaria MiletiTom DeFeliceIlda AbeTom WhyteIldus KhasanovTomas BrandaoIleana BaldiTomás Gómez Álvarez-ArenasIleana PirovanoTomas JandaIleana-Diana NicolaeTomáš MartinecIl-Gu LeeTomáš PotužákIliana MarinovaTomáš ŘezníkIlias MavroidisTomás Salgado-JiménezIlija BašičevićTomás TorrobaIlker PolatogluTomaso VairoIlker S. BayerTomasz BakonIlkka NissinenTomasz BieniekIlknur TelkesTomasz CudejkoIllyas Bin Md IsaTomasz FalkowskiIlma MufidahTomasz FiglusIlsun RhiuTomasz GóreckiIlya JacksonTomasz GrzesIlya ShenderovichTomasz JakubowskiIlya TurchinTomasz KapuscinskiIlya VolodinTomasz KisielIlyas PotamitisTomasz KryjakIlyes KhelfTomasz MarciniakIlyos KhujayorovTomasz NiedobaImad RidaTomasz NowakowskiIman AbduljaleelTomasz OrczykIman AskerzadeTomasz OsuchIman IzadgoshasbTomasz PanderIman Soltani BozchalooiTomasz PetelenzImed Ben DhaouTomasz RakImranTomasz RokickiImran AliTomasz RunkaImran Mohd IbrahimTomasz SzczegielniakImran ShahTomasz SzymborskiImran TasadduqTomasz TeleszewskiImre CikajloTomasz WandowskiImre KissTomasz WasilewskiImtiaz MahmudTomasz WojciechowskiIn Young KimTomasz WolnyInacio YanoTomi T. LiInam UllahTomislav KeserInam Ullah KhanTomislav LetnikInayat KhanTomislav LipicInbarasan MunirajTomislav MaticInder GuptaTomislav RožićInderjeet SinghTomislav StipancicIndika KumaraTommaso BragattoIndrajith D. NissankaTommaso CardonaInès ChihiTommaso ErcoliInes DelfinoTommaso FedulloInes DespotovićTommaso GiovanniniInes KožuhTommaso GrecoInga Griskova-BulanovaTommaso PolieroIngo AhrnsTommaso ZoppiIn-Ho LeeTomoaki OhtsukiInho NamTomoki MaedaIn-ho RaTomoki MatsudaIñigo González De ArrietaTomoya NakataniInjamamul AriefTomoyuki MiyamotoInma Mohino-HerranzTong WangInmaculada AparicioTongchuan WeiInmaculada Ortiz-GómezTonggang ZhangInna BukreevaTongyu DaiInna E. StepanovaTongyun LiInna Skarga-BandurovaTonko KovacevićInsaf UllahTony JanInsung IhmTony LuczakIntan Rosalina SuhitoTony ZhangIoan BurdaToomas RangIoan SusneaToralf BeitzIoan TudosaToru KuriharaIoan UngureanToshiaki TsujiIoana CiuciuToshihiko NakataIoana-Gabriela SirbuToshihiko YoshinoIoan-Dragos DeaconuToshiki KanamotoIoanna KyriakouToshio ItohIoannis AnagnostopoulosToshitaka WakayamaIoannis ApostolopoulosToshiya ArakawaIoannis D. ChasiotisToshiyo TamuraIoannis HatzilygeroudisTouhidul AlamIoannis KarabagiasTouqeer AhmadIoannis KarydisTove HelldinIoannis KatsidimasToyoaki OhbuchiIoannis KortidisTracy Anne HammondIoannis LiritzisTraian MaziluIoannis NalbantisTraianos V. YioultsisIoannis Nestorios ParasidisTrent W. BiggsIoannis NtintakisTresna DewiIoannis O. VardiambasisTri Dev AcharyaIoannis P. GravasTri Niswati UtamiIoannis T. GeorgiouTrieu NguyenIoannis TheologidisTrojnacki MaciejIoannis TsimperidisTrong-The NguyenIoannis TsoulosTrong-Thuc HoangIolanda Valentina PopaTrung (Tim) LeIoli PanidiTrung Duy TranIon BogdanTrung Thanh NgoIon MironescuTrung-Kien DaoIon SanduTruong Cong ThangIonel ChiricaTshiamo SigweleIonel ZaganTsubasa MaruyamaIonica OncioiuTsuji ToshilhiroIonuţ Daniel GeoneaTsukuru MinamikiIonut Emil IacobTsuneo HoriguchiIonuţ MineaTsuneyoshi SugimotoIonut SchiopuTsung-Chin HouIosif Vasile NemoianuTsung-Han TsaiIosko BalabozovTsung-Heng TsaiI-Pin ChangTsung-Hung LinIppokratis SartzetakisTsung-Lin ChenIqbal QasimTsu-Yang WuIqram HussainTsz Him ChowIra ValenzuelaTsz Nam ChanIren ValovaTuan Anh NguyenIrena GalićTuan-Hung VuIrena Ištoka OtkovićTuan-Minh PhamIrena IvaniševićTuan-Vinh LeIrena JekovaTuanwei XuIren-Adelina MoldovanTuchen HuangIrene Lara-IbeasTudor PaladeIrene NutiniTudor PopIrene TagliaroTudor SalageanIrene VilàTugrul YakupogluIreneusz DominikTu-Liang LinIreneusz KubiakTullio GiuffrèIrfan Ul HaqTunc AsurogluIrina A. BorodinaTuomas HietaIrina B. VendikTuong LeIrina E. BocharovaTurker DagdelenIrina Georgiana MocanuTurker TuncerIrina IonicaTurki TurkiIrina PicioroagaTushar Kanti RoyIrina S. KipyatkovaTyler BellIrina SeverinTymoteusz HorbińskiIrraivan ElamvazuthiTyson ReimerIs FatimahTzu-Hsien SangIsa AnshoriTzu-Ling ChenIsaac Machorro-CanoTzung-De LinIsaac NooniTzung-Han LinIsaac Oyeyemi OlayodeU. Mohan RaoIsabel EscobarU. UbaidillahIsabel Martínez-AlcaláUan Carlos Briede-WestermeyerIsakovic AbdelUchenna DialaIsibor Kennedy IhianleUday Kumar SinghIsidora StankovicUday TupakulaIsidoro Garcia-GarciaUdo FreseIsidro CalvoUdo SchilcherIslam MosaUferah ShafiIslam Taj-EddinUgljesa MarjanovicIsmael LenguaUgo Della CroceIsmael PayoUgur AlganciIsmail AhmedUgur AvdanIsmail FidanUğur ErkanIsmail ShahUgurhan KutbayIsmini E. PapageorgiouUillian DreyerIsrael David Hinostroza SáenzUjjwal Kumar DeIsrael Hinostroza SaenzUk SimIsrael Miguel AndrésUkadike Chris UgbolueIstván BodnárUliana MonakhovaÍtala MarxUlrich MeschederItamar LeviUlrich TimmItay BarneaUlrike KluehItthipon JeerapanUlrike SteinmannItzamá López YáñezUlysses BernardetIulian PetrilaUma N. DulhareIuliana MarinUmair IqbalIva ChianellaUmair KhanIva LakićUmamaheswari RajajiIvalina AvramovaUmapathi ReddicherlaIvan A. AleksandrovUmapathy SnekhalathaIvan A. NikolovUmar DrazIvan A. ParinovUmar IqbalIvan AleksiUmar OzgunalpIvan BratchenkoUmberto AmatoIvan CamponogaraUmberto GaliettiIvan CukUmberto RaucciIvan ĐurekUmer SaeedIvan FerrariUmesh PatiIvan GiorgioÜmit AydinIvan IudiceUmit OgrasIvan IvanovÜmit ŞentürkIvan JovovićUmut ÖzkayaIvan Julian-RochinaUnal DevrimIvan Kristianto SinggihUnit Three KartiniIvan Lim Chen NingUpul GunawardanaIvan MachadoUrban M. FietzekIvan MarićUriel Haile Hernandez-BelmonteIvan MatveevUrmita SikderIvan Miguel PiresUroš ČakarIvan P. ChristovUrszula BłaszczakIvan PavlenkoUrszula KużelewskaIvan PetruninUrszula MalinowskaIvan PopovićUrszula SomorowskaIvan PuchadesUsman GhafoorIvan RacetinUsman HabibIvan RogovskyiUsman TariqIvan StoikovUtkal Ranjan MuduliIvan SyniavskyiUtpal N. RoyIvan VirgalaUtsav BanerjeeIvana IvanićUwe FrießIvana MihajlovićUwe HampelIvana NovakovicUwe RitschelIvana PanžićUzair IqbalIvanna DronyukUzair SajjadIvanovitch SilvaV. K. UnnikrishnanIvar ZekkerV. M. ManikandanIve WeygersVaclav BuncIveta ZolotováVáclav KrysIvica LukićVaclav MatousekIvica PetrovicVáclav NežerkaIvo AllegriniVáclav RancIvo SilvaVáclav ŠmídlIvo StachivVaclava PioreckaIvona ZakarijaVadim A. ParfenovIvor NissenVadim BolshevIwan DarmadiVadim FetisovIwan SetyawanVadim KramarIwona GrabarekVadim R. GasiyarovIwona GrabowskaVadim V. GrubovIwona GrobelnaVadim ZhmudIwona PaprockaVadym AvrutovIyyakutti Iyappan GanapathiVahab Youssof ZadehIza Sazanita IsaVahid AbolghasemiIzabela Luiza PopVahid AkramIzabela PiechVahid NasirIzabela RejerVaidas PacebutasIzabela Sadowska-BartoszVaidotas BarzdėnasIzabela ŚliwaVaidotas MarozasIzabella KrucińskaVaiju KalkhambkarIzaz RaoufVairavasundaram IndragandhiIztok FisterVairavasundaram SubramaniyaswamyIztok KrambergerVaiyapuri GovindasamyIzzah ShahidVálber César Cavalcanti RozaJ. Andrew OnesimuValdecir Antonio PaganinJ. Sathish KumarValderi R. Q. LeithardtJabar H. YousifValene Garr BarryJabbour ChadiValentim RealinhoJacek CabanValentín BarralJacek GębickiValentin BuiculescuJacek IzydorczykValentin Cardeñoso-PayoJacek JakuszValentin IoniţǎJacek LubczonekValentina FranzoniJacek Łukasz Wilk-JakubowskiValentina GecevskaJacek MarciniakValentina GraciJacek MatysValentina MarascuJacek MichalskiValentina PalazziJacek MisiurewiczValentina ValleJacek NowakowskiValentina VassilenkoJacek OlenderValentina YakovlevaJacek OskarbskiValentina ZegaJacek PaśValentino PelusoJacek RozanskiValentino SantucciJacek SalachValeria BelluscioJacek SobczykValeria Cesario TimesJacek SzmaglińskiValeria GiampaoloJacek WilczyńskiValeria LupianoJacek Wilk-JakubowskiValeria Soto-MendozaJacek WozniakValeria SpizzichinoJacek WytrębowiczValeria-Ersilia OnigaJacek ZebrowskiValerii OstrovskiiJack MerrinValerio BaiocchiJacob BergslandValerio BeniJacobus MullerValerio BiancalanaJacopo BoagaValerio De LucaJacopo SiniValerio Flavio GiliJader CabralValerio MarianiJadwiga SołoduchoValerio ScordamagliaJae Hong ShimValeriu Manuel IonescuJae Hoon LeeValeriu VrabieJae Joon KimValeriy KrivetskiyJae Min LeeValery KovalevJae Mok AhnValery V. KalinchukJae One LeeValliappan RamanJae Seang LeeVallikutti SathananthavathiJae Yeol LeeValner BrusamarelloJaechang ShimVamsi BorraJaehan KohVamsi InturiJae-Hoon KimVamsi Krishna PasamJaehwa ParkVan Hoang LuanJae-Hyun KimVan Linh NguyenJaehyun YooVan Nam GiapJaejong ParkVan Thanh NguyenJaemin BaekVan-An DuongJae-Myung RyuVandana KhannaJaepil KoVander Alkmin RibeiroJaesun LeeVandoir BourscheidtJaewon NamVan-Duc TranJae-Young ChungVanesa Fernandez-CaveroJae-Young JangVan-Hai BuiJaeyoung LeeVan-Hung LeJaeyul LeeVânia GuimarãesJafar AlzubiVarameth VichiensanJafar TavoosiVardan ApinyanJagadeesh PasupuletiVarlei RodriguesJagannath AryalVarsolo C. SunioJagat Jyoti RathVarun GuptaJagatheesan AravinthVarun JeotiJagdeep RahulVarun Narayan Mishra MishraJagriti NarangVaruna De SilvaJagriti SainiVasa RadonicJahangir MasudVasanth IyerJai OtmanVasco PoncianoJai PrakashVasil D. SimeonovJaime Andrés Pérez TabordaVasil YordanovJaime DevineVasile A. PopescuJaime Duque-DomingoVasile BaltacJaime LloretVasile-Gabriel IanaJaime Martinez-CastilloVasileia PapathanasopoulouJaime Moll De AlbaVasileios C. KapsalisJaime MorenoVasileios GkioulosJaime OrtegonVasileios MemosJaime Vásquez-GómezVasileios VlachosJain-Shing LiuVasiliki KinigopoulouJairo Alexander Osório SarazVasiliki N. IkonomidouJairo LópezVasiliki VasilatouJaiteg SinghVasiliki VitaJaka SodnikVasilis ChristofilakisJakaria RabbiVasilis DimitriouJakub BernatVasilis EvagelopoulosJakub CiazelaVasilis KanakoudisJakub HorákVasiliy ElaginJakub KrejčíVasiliy OsipovJakub Krzysztof GrabskiVasily DesnitskyJakub LengiewiczVasko JovanovskiJakub NalepaVassilios C. MoussasJakub Sławomir GąsiorVassilios KappatosJakub WagnerVassilis G. KaburlasosJakub WięckowskiVassilis PapadakisJakub ZdartaVassilis PoulopoulosJalal Ali Sadeq AbdulhadiVasyl TeslyukJalal MoradiVeasna SoumJalal Taheri KahnamoueiVedang ChauhanJamal MabroukiVedran BudinskiJamal NabulsiVedran MrzljakJames BowenVeenu MangatJames ChowVeerabhadrappa T.James Chung-Wai CheungVeerachai TanpipatJames CovingtonVeeramanickam MurugappanJames HahnVeeraraghava Raju HastiJames J. WolffsohnVelibor IlicJames L. OldsVeljko PetrovićJames M. GriffinVeljko SrzicJames MagidiVenkata Chaitanya ChirumamillaJames P. McGilliganVenkata Lakshmi Narayana KomanapalliJames P. WissmanVenkata Nagesh Kumar GundavarapuJames ReynoldsVenkata Narayana PalakolluJames SpicerVenkata P. YanambakaJames T. GristVenkata Prasanth YanambakaJamie WubbenVenkata Ravibabu MandlaJamshaid Ul RahmanVenkata Surya N. ChavaJamshed Ahmed AnsariVenkataiah GorigeJamshed IqbalVenkatakrishnan RengarajanJán BačíkVenkatesan RajinikanthJan BarwickiVenkatesh VeeraraghavanJan BudroweitVera GramignaJan CornelisVera Z. PérezJan EggerVeraldo LiesenbergJán ErdélyiVeralia Gabriela SanchezJan FlusserVered Silber-VarodJan FrancVern HartJan GebauerVeronica EgidoJan H. SchmidtVerónica Gracia-IbáñezJan HavlíkVeronica SberveglieriJan HeřmanVeronica SofianosJan HofsteeVeronika KovacovaJan HošekVeronika SzücsJan J. DubowskiVeselin N. IvanovicJán KizekVesna BlagojevicJan KleinertVesna Popovic-BugarinJan KlimaszewskiVessela KrastevaJan KoltermannVessela TsakovaJan KomarekVetturi DavidJan KoubaViacheslav OsadchyiJán LabunVibha PatelJan LancokVicente Ferreira De LucenaJan LatalVicente Hernández DíazJán LižbetinVicente J. P. AmorimJan MaresVicente Martí-CentellesJan MućkoVicente Matus IcazaJan NikodemVicente ParotJán PapajVicente Romo PérezJan PetersenVicente S. Fuertes-MiquelJán PiteľVicente TraverJan PoelaertVicenza TorrisiJan PolzerVictor A. S. V. BittencourtJan SaligaVictor CharpenayJan SchürVíctor Cuadrado-PeñafielJan ŠebekVictor Diogho Heuer De CarvalhoJan SkalkaVictor Fernandez NascimentoJan StroblVictor GuerraJan TuranVictor H. DuenasJán VachálekVictor J. BarrancaJan Willem BorstVíctor Javier Cruz-DelgadoJan ZebrowskiVictor KornevJana ChovancováVictor KotlyarJana Faganeli PucerVíctor M. CampelloJana JeffersVictor MilenkovicJanaina Antonino PintoVíctor Monzón BaezaJanez TronteljVictor MultanenJanghoon YangVíctor MurrayJangPyo BaeVictor RamosJanina BahnemannVíctor Ruiz-Valdepeñas MontielJanis AlnisVictor SanchezJanis BalodisVictor SantibañezJanis BraunfeldsVictor SysoevJanis JudvaitisVictor TcherdyntsevJanis SpigulisVictor VillagráJanja Dermol-ČerneVictor-Alexandru BriciuJanos BotzheimVictoria A. RyzhovaJanusz DudczykVictoria HodgeJanusz JakubiakVictoria ShpacovitchJanusz Marian BȩdkowskiVidal MorenoJanusz NarkiewiczVidas ŽuraulisJanusz RomanikViet Anh TruongJanusz RusekViet Thanh NguyenJanusz SmulkoViet-Thanh PhamJanusz SosnowskiVignesh SureshJanusz StrzeleckiVigouroux NadineJanusz SzpytkoVijander SinghJanusz WaloVijay BabbarJaouhar FattahiVijay Bhaskar SemwalJarmo AlanderVijay KakaniJaromír HradVijay KumarJaromír KlarákVijay Kumar VermaJaroslav HlavaVijay TambrallimathJaroslav RozmanVijayachandra RamachandraJarosław ChodórVijayakumar PonnusamyJarosław DomaszewiczVijayanandh RajaJaroslaw GalkiewiczVijey ThayananthanJaroslaw JanusVika G. GrigorievaJarosław Jaszczur-NowickiVikas Anand SaharanJaroslaw KrygierVikram MailthodyJarosław KurekVikram MittalJaroslaw PytkaViktor MolnárJaroslaw SelechViktor SydorukJarosław TokarczykViktor TaneskiJarosław W. KłosViliam HromadaJarosław ZubrzyckiVille KaajakariJaser Sa′EdVilma RatautaitėJasmina Lozanovic SajicVilmos BilickiJasmine YanVimal Kumar Singh YadavJason BeechVimal ShanmuganathanJason BrenkerVinay GautamJason FriedmanVinay K. KayethaJason G. ZeibelVinay Kumar JadounJatinder ManhasVinayak ElangovanJavad GhalibafanVinayak VijayanJavad GhofraniVinayakumar RaviJaved SayyadVincent NyangeresiJaveed HussainVincent Omollo NyangaresiJaveed ShaikVincenzo ContiJavier Del CampoVincenzo CostanzoJavier Díez-GonzálezVincenzo De AngelisJavier Garcia-GuzmanVincenzo DeufemiaJavier Lopez-SolanoVincenzo GuidiJavier Macias-GuarasaVincenzo Mariano MastronardiJavier Martínez TorresVincenzo MazzaracchioJavier Ortuño SierraVincenzo NiolaJavier Sanchez-EspigaVincenzo PalleschiJavier ToledoVincenzo Romano MarrazzoJawad AhmadVincenzo Saverio AlfioJawameer HamaVincenzo VespriJawwad LatifVinh Thong TaJay M. KhodadadiVinh Truong HoangJay SinghVinh Tung LeJayantilal N. PatelVinh Van TranJayashri KulkarniVinícius ManzoniJayavelu Udaya PrakashVinicius RofattoJayendra KumarVinod BelwanshiJayme Garcia Arnal BarbedoVinod KushvahaJayoung KimVinod MishraJayshri KulkarniVinoth Babu KumaraveluJa-Yu LuVioleta LazicJea Uk LeeVioleta MonasterioJean Christophe CousinVioleta NiculescuJean D. Hallewell HaslwanterVioleta PurcarJean Paul DouzalsVioleta SimionJean-Baptiste ThomasViorel GoanţăJean-Charles BolomeyViorel PaleuJean-Fu KiangViorica Rozina ChifuJean-Jacques PoncianoVipin BalyanJean-Marie SleewaegenVipul A. ShahJean-Michel LegerVira ShendrykJean-Paul CalbimonteViraj MuthugalaJean-Philippe DidonVirgil DobrotaJean-Pierre BarriotVirgil DumbravaJean-Pierre BrescianiVirginie FelizardoJean-Pierre HuignardVisar FarhangiJee-In KimVishal DwivediJeesu KimVishal SharmaJeevithan ElangoVishal SrivastavaJee-Youl RyuVishnu S. PendyalaJeffrey A. TuhtanVishnu SureshJeffrey AmelseVishwanath BijalwanJeffrey C. F. HoVit NovacekJeffrey K. WuenschellVitali F. NesterenkoJeffrey NeashamVitalii I. SysoevJeffrey PengVitalii IvanovJegadeeshwaran RakkiyannanVitaliy B. MykhaylykJeganathan ChockalingamVitaliy YakovynaJehad AliVitaly LevashenkoJehan-Antoine VayssadeVito PagliaruloJehangir ArshadVito TagarelliJeih-Jang LiouVítor CarvalhoJelena D. VujančevićVitor FialhoJelena MuncanVitrice Ruben Folifack SigningJelena PopovicVittorio AstaritaJelena StankevicieneVittorio MemmoloJelena Vapa-TankosicVittorio RampaJelena VidakovićVivek P. ChavdaJeng-Shyang PanVivi TornariJeniffer CarrilloVivian VimarlundJennifer BunnViviane PillaJennifer HowcroftVjaceslavs LapkovskisJennifer M. VojtechVlad CarleucuJennifer N. A. SilvaVlad DiaconitaJenn-Kaie LainVlad PopescuJenny HuangVladan PapicJens JensenVladeanu CalinJens KirchnerVladimir AnfinogentovJens NeuVladimír BulejJens Onno KrahVladimír ChmelkoJens-Peter ZöllnerVladimir DivićJen-Yuan (James) ChangVladimir DmitrievskiiJen-Yung LinVladimir DyoJeon LeeVladimir E. ZharovJeong Geun KimVladimir GurauJeong Jae WieVladimir HeiskanenJeong Min KangVladimir JotsovJeong Sik LimVladimir JoukovJeongguk KimVladimir KasikJeong-Hwan KimVladimir KazeiJeonghyun KimVladimir KnyazJeongmoo HuhVladimir KolesovJeongyeup PaekVladimir KorkhovJerdvisanop ChakarothaiVladimir KozlovskiyJernej RoskerVladimir KuptsovJernej VičičVladimir L. KodkinJeroen BergmannVladimir L. VaksJeroen MeersmansVladimir LukinJérôme LaurentVladimir MilovanovicJérôme Tchoufang TchuindjangVladimir RajovićJerome ThevenotVladimir RazevigJerónimo Aragón-VelaVladimir ReshetovJeronimo Segovia-FernandezVladimir S. ShiryaevJerry ChouVladimir SeredaJerry Chun-Wei LinVladimir ShakhovJerry TrahanVladimir SinitsinJerzy BalickiVladimir SkripnyakJerzy BaranowskiVladimir StojanovicJerzy JackowskiVladimir TadićJerzy JózwikVladimír TlachJerzy KisilowskiVladimir UlanskyJerzy KonorskiVladimir V. ArlazarovJerzy ŚwiątekVladimir Yu. VenediktovJerzy SzyszkaVladimíra BiňasováJerzy Wojciech MietelskiVladislav DemyanovJerzy WtorekVladislav KalinnikovJés De Jesus Fiais CerqueiraVladislav KuchanskyyJesenka PibernikVladislav ToronovJesse Pentzer PentzerVlado DelićJesse RumboVlastimil MasekJesús Ángel Román GallegoVoitech StankevicJesus Antonio Sosa HerreraVojkan DavidovicJesus Eduardo LugoVojko MatkoJesús Emmanuel Solís-PérezVojtěch PetruchaJesus Enrique GarcíaVolkan AydogduJesús Enrique Velázquez-PérezVolker KörstgensJesus Fernández GaviraVolker LohwegJesus Garcia SanchezVolker SteinhageJesús García-HerreroVolkov PetrJesus GrajalVolodymyr MosorovJesús Hernández-SazVolodymyr PonomaryovJesús M. Vegas HernándezVolodymyr SokolovJesús María López-LezamaVorathin EpinJesus MirapeixVrchota JaroslavJesús Pablo Lauterio-CruzVsevolod YutsisJesús Pérez-RíosVule ReljićJesús Sáez PadillaVusi Mpendulo MagagulaJesús Sergio Artal-SevilVyacheslav RybinJesus TejadaVytautas OstaseviciusJesus VilladangosVytautas PaulauskasJeungchan LeeVytautas RudžionisJeyeon KimW. G. C. W. KumaraJhantu Kumar SahaWadii BoulilaJhilik BhattacharyaWael A. AltabeyJi Hwan ParkWael Abdullah AhmadJi WangWael AhmedJia KeWael El-DeredyJia LiWael Hosny Fouad AlyJia LiuWael Mohamed Shaher YafoozJia PengWael SaabJia RongWafa ElmannaiJia SongWafaa SalehJia UddinWagner Coelho De Albuquerque PereiraJia WangWagner Ourique De MoraisJia WuWahab MohyuddinJia YanWahaj Abbas AwanJiafeng FengWaheb AbdullahJiaguang KanWahyono WahyonoJiaguo ZhangWahyu CaesarendraJiahuan JiangWahyu Hendra GunawanJiahui ChenWahyu RahmaniarJiajie LiWahyu SrigutomoJiakai LiWai Tung NgJiamao LiWai-Hang KwongJiameng FanWala SaadehJiaming WuWaldemar C. Leite FilhoJiamu CaoWaldemar JendernalikJian FengWaldemar MinkinaJian LiangWaldemar OdziemczykJian LuWaldemar SwiderskiJian PangWaldir SabinoJian SunWaleed Abd-ElhameedJian WangWaleed AliJian Wei TayWalid El-ShafaiJian WuWalid HariziJian XuWalter ChenJian YangWalter E. KaufmannJianan WangWalter HolwegerJianbang GeWalter J. SalcedoJianbin LiuWalter LegnaniJianbing ShenWalter MaetzlerJianchao PangWalter Takashi NakamuraJiancheng LaiWan Azani MustafaJiancheng ZhengWan Hazman DanialJiandong WuWan KhairunizamJianfa WuWanbin RenJianfei SunWanda UrbanovaJianfeng ChenWandah WibawantoJiang GuoWanghui BuJiangbin LyuWanguo JiaoJiangbo XuWangyan LiJiangchen LiWanhao CaiJiangpeng HeWanheng LuJiangtian YangWanich SuksatanJianguo LiuWan-Jung ChangJiangzhen GuoWanke LiuJian-Hong LiuWanli WenJianhong YangWann-Yun ShiehJianhui YuWanquan LiuJianing SongWan-Xing YangJianing WuWaqar Shahid QureshiJian-Jiun DingWashington Yotto OchiengJianjun ChenWasim DarJianjun TongWasiq KhanJiankai YuWasiq UllahJiankang HuangWayne MooreJianlei CuiWei ChenJianliang SunWei ChengJianlong JiWei GaoJian-Long KuoWei GuanJianmei WangWei HeJianming WenWei HuJianping ChenWei JiJianping WangWei JinJianqi WangWei LiJianqing PengWei LinJianqing WuWei LuJianshuang LiWei Min HuangJiantao YaoWei PanJiantong LiWei QianJian-Wei LiWei Ru WongJian-Wei LiuWei ShangJianwei ShuaiWei ShaoJianwen MengWei SongJianwu DouWei SuJianxiao MaoWei TangJianxin JiaWei WangJianxing LiuWei WuJianxiong GuoWei XingJianxiong ZhuWei YanJianyi ZhangWei YinJianyu FuWei YuanJianyu GuWei ZhaoJianzhong HaoWei ZhouJianzhong ZhangWei ZhuangJianzhou WangWeibing LuJianzhu HuaiWeibing WangJiaoyang LiWei-Bo ChenJiaqi JuWei-Chang YehJiaqi LiuWei-Che ChienJiaqi XuWeicheng CuiJiaqi ZhuWeicheng LinJiashen TehWei-Chiang HongJia-Shing SheuWeichuan ZhangJiawei ChangWeicun ZhangJiawei ChenWeida HuJiawei XiangWeidi LiuJiawei YaoWeidong KuangJiawen HuWeidong YangJiaxuan LiaoWeidong YuJiayao ChenWeifeng JinJiayuan LiWeifeng PanJiayuan LinWei-Feng SunJiayuan ZhuangWei-Han ChenJia-Yush YanWeihe XuJiazeng ShanWeihua HuJibin ZhengWeihua LiuJibum KimWeihua OuJichao HongWeijie LuoJicheng YuWeijie TanJidesh PadikkaWeijin GuoJidesh PadikkalWeijing ShiJidong J. YangWei-Kai ChengJidong WangWeili DengJie ChenWeili ZhangJie GuoWeilin YeJie HanWeimin ChenJie JiangWeimin HuangJie LiWei-Min LiuJie ShaoWeimin ZhouJie SunWeiping FuJie TianWeiping LiJie YangWeiping WenJie ZhangWei-Ping ZhuJiehao LiWeiqing MinJiehui LiWeishi ZhangJieru YaoWei-Shun LiaoJiewen LaiWeiwei DuJifa GuoWeiwei JiangJiheng LiWeiwei SunJih-Fu TuWeiwen ZhangJihong LiuWeixia ZhangJihoon MoonWeiyan HouJihwan MoonWeizhen HouJihyoung ChaWeizhong TianJijia WangWeizhuo WangJikai WangWellington Pinheiro Dos SantosJim TangWen Chang HuangJiming SongWen DongJimmy Aurelio Rosales-HuamaníWen LiJin BenWen LuJin HeWen SunJin LiWen WangJin LiuWen ZhangJin LuWenai ShenJin NingWenan YuanJin QiWenbin ZhangJin WangWenbo LiuJin YanWenbo ZhouJin YangWenchen MaJin Young LeeWencheng GuoJin YuanWen-Cheng KuoJin-A ChoiWen-Cheng LaiJinan GuanWenda LiJinbo WuWendi WeimarJinbo XieWendy Flores-FuentesJinbo XiongWen-Fang XieJinfeng LiWenfei GuoJing BaWenfeng HaoJing HanWenfeng ShenJing LiuWenfeng ZhengJing QiuWenge GuoJing QuWen-Hsi ChengJing WangWenhui PeiJing WeiWen-Hwa LiaoJing WenWenjie ZuoJing XuWenjin WuJing YangWenjing HuangJing ZhangWenju LiuJing ZhouWenjuan ZhangJing′an LiWenjun (Chris) ZhangJingang LiWenjun WuJingcao CaiWenkai LiJingdong ZhangWenlong FuJingfeng HuangWenlong LiJin-Ghoo ChoiWenlong ZhangJinghua LiWen-Nung LieJingjing XuWen-Pin HuJingjing YangWenping MaJinglun FengWenqiang CuiJingmin YangWen-Ran ZhangJingru YiWen-Shan ChenJingui MaWensheng ZhaoJingwei SongWentao ZhangJingwei ZhangWenting DaiJingyu WangWenxi ChenJinhua LiWenxu ZhangJin-hui ChenWenya ZhouJinhui SongWenyuan LiuJinhyoung ParkWenzhen YueJin-Jia HuWenzhu HuangJinjin YanWerner BrockherdeJin-Kyun LeeWerner M. GrafJinlong LiWerner MänteleJinlong LiuWesley BeccaroJinlong XiaoWhai-En ChenJinlong YuanWidiastuti SetyaningsihJinping LiuWiesław LeonskiJinquan LiuWieslaw PajaJinrong HeWiktor B. DaszczukJinse ParkWiktor HaleckiJinsheng LuWiktoria WilkowskaJinshuo SuWiktoria WojniczJin-Shyan LeeWilbert L. IJzermanJinsong WuWilfrid BoireauJinsu GimWilkley Bezerra CorreiaJin-Taek SeongWilliam ArrasmithJinwei CaoWilliam C. WilsonJin-Wei ShiWilliam Edward LeeJinwei SunWilliam HammondJinwoo JangWilliam J. KirkwoodJin-Woo JeongWilliam KaalJinwook KimWilliam KleindlJinwu KangWilliam RomineJinxing LiangWilliam ScheidelerJinyang JiaoWilliam WangJinzhou ZhuWillian DimitrovJiong ShiWillie LeungJiong ZhouWilmar HernandezJiří JandaWilson Cesar Sant′AnaJiří Jaromír KlemešWilson RamírezJiří KrejsaWinko W. AnJiri KroutilWinston PercybrooksJiri MaxaWioletta Mędrzycka-DąbrowskaJiri PlasekWislei Riuper OsórioJiri PrinosilWitold ByrskiJiří ŠilhaWitold KazimierskiJiri TesarWitold WęglewskiJiri ValaWładysław SkarbekJitendra Narayan DashWłodzimierz FredaJitendra PalWłodzimierz KasprzakJitendra PrajapatiWoei-Jiunn TsaurJiuling LiWojciech FabianowskiJiun-In GuoWojciech GawlikJiwoo KangWojciech GiernackiJi-Woong ChoiWojciech GregaJiyoung JungWojciech KaczmarekJiyu LiWojciech KieratJize YanWojciech LitwinJizhan LiuWojciech PaprockiJo Woon ChongWojciech PaslawskiJoachim ClemensWojciech PietrowskiJoachim SchnurbusWojciech SałabunJoan A. Ruiz-de-AzuaWojciech SikorskiJoan BasWojciech SkrzeczanowskiJoan BausellsWojciech SteczJoan CasasWojciech SulejJoan Vila-FrancésWojciech WaloszekJoana PereiraWojciech WięcławekJoan-Francesc Fondevila-GascónWojciech WolanśkiJoanna CzajkowskaWojciech ZaganJoanna DipnallWolfgang BeinJoanna IwaniecWolfgang HübnerJoanna MichalowskaWolfgang LeisterJoanna MystkowskaWolfgang SchreinerJoanna OrtylWolfram SpreerJoanna Piotrowska-WoroniakWon BaeJoanna RutWon Chul LeeJoanna Sekulska NalewajkoWon Hee LeeJoanna ZajdaWong Chin HongJoão Alberto Passos FilhoWon-Jae RyuJoão AvóWonkeun ChangJoão Carlos GiacominWontae KimJoão CastelhanoWoo June ChoiJoão CoblinskiWoo Sik YooJoão CostaWoo-Jin ChungJoão F. OliveiraWoojun ChoiJoão Fernando Pereira GomesWooyeol ChoiJoão KleinschmidtWorradorn PhairuangJoão Lucas Della SilvaWouter Baernd Verschoof-van Der VaartJoão M. P. CoelhoWu ChenJoão M. P. Q. DelgadoWubin WengJoão Marcelo TeixeiraWudhichai AssawinchaichoteJoão Nuno MatosWujie ZhouJoão Paulo do Vale MadeiroWujun ChenJoão Paulo LimaWujun SiJoão Paulo Pereira do CarmoWuliang YinJoão Paulo Ramos TeixeiraWuqiang YangJoão Paulo WiniarskiWusheng ZhaoJoão Pedro Matos-CarvalhoWuyi MingJoão Pedro PaviaWuyong TaoJoão PetricaXavier FernandoJoão ReisXavier García-MassóJoão RibeiroXavier Larriva-NovoJoaquim AzevedoXenophon ZabulisJoaquim ChalerXhevahir BajramiJoaquim Gabriel MendesXi PengJoaquim Manuel Trigo MarquêsXia LiJoaquim SousaXia WeizhongJoaquin ChungXian SunJoaquín LuqueXian TaoJoaquin Paez-MoguerXian ZhaoJoaquín Torres-SospedraXian-Bo WangJob BeckersXianchuang ZhengJobst WurlXiandong LengJoceli MayerXiang LiJochen LangXiang QianJoe FlemingXiang XuJoel Alter GreenbergXiangang LuoJoel Antonio Trejo SánchezXiangbing ZhouJoel MartinXiangdong ChenJoel ParthemoreXiangdong WangJoeri GerloXiangjie KongJoginder Singh DuhanXiangjun LiJohan CassirameXiangjun ZouJohan J. Estrada-LópezXianglin LiuJohan N. Van Der MeerXianglong WanJohan WahlströmXiangnan HeJohan ZetterbergXiangning LuJohann MartonXiangrong ShenJohanna NärväinenXiangtao LiJohannes BetzXiangtao ZhengJohannes FresnerXiangwei KongJohannes GruenwaldXiangwei ZhuJohannes HinckeldeynXiangxi MengJohannes SchelvisXiangxiong KongJohannes SchobelXiangyu GaoJohannes StrunkXiangyu GeJohannes VorwerkXian-Hua HanJohn BellXianjian JinJohn BlakeXianlin ZengJohn D. ChaseXianming LangJohn D. McCamleyXianta JiangJohn DermentzoglouXianwen WangJohn E. Candelo-BecerraXiao ChenJohn EnrightXiao LiJohn Fredy Barrera RamirezXiao LiuJohn GialelisXiao TianJohn GreenhallXiao XiaoJohn I. B. WilsonXiao YuanJohn J. GuersXiao ZhangJohn Jairo Villarejo MayorXiao(Kieran) WangJohn KalomirosXiaobao YangJohn NassourXiaobin YinJohn Ojur DennisXiaobing DengJohn R. SaffellXiaobing LiJohn Richard JenningsXiaobing PangJohn S. HoXiaobing YuJohn S. MitchellXiaochen GuoJohn ShutskeXiaochuan XuJohn StanleyXiaochuan ZhangJohn ThomasXiaochun ChengJohn TsiligkaridisXiaocong YuanJohn William BranchXiaodong WangJohn WilliamsXiaofeng LiJohn-Lewis Zinia ZaukuuXiaofeng WangJohnn P. JuddXiaofeng ZhouJoko MariyonoXiaoguang DongJolanta GawalekXiaoguang JiangJon Glenn GjevestadXiaohua (Edward) LiJon YounXiaohua HuangJonas A. PramuditaXiaohui HanJonas AudaXiaohui LiJônata Tyska CarvalhoXiaojiang PengJónatas ValençaXiaojie XuJonathan RealmutoXiaojing MuJonathan S. WeerakkodyXiaojun TanJonathan S. ZiaXiaoliang WangJonathan SeyboldXiaomei ZhangJonathan YuenXiaoming DengJonay ToledoXiaoming ZhangJones Luís SchaeferXiao-Nan ZhangJong Chan HongXiaoning ShiJong Seok BaeXiaoning TangJongbeom LimXiaopeng HuJong-Chan KimXiaopeng LiJong-Ho ShinXiaoqiang DiJong-Hoon WonXiaoqiang LiJonghyun ChoiXiaoqiang YanJonghyun KimXiaoqin ZangJong-Hyun KimXiaoqing LvJonghyun OhXiaoqing WuJong-Myon KimXiaoqiong QiJongpil JeongXiaorui ShaoJong-Ryul ChoiXiaoshan BaiJong-Seok KimXiaoshuang LiJong-Seok OhXiaosong WangJongseong Brad ChoiXiao-Song ZhuJongshill LeeXiao-Ting HeJongsub MoonXiaowei ZhouJong-Uk HouXiaoyan KangJongwon KimXiaoyang KangJongwon ParkXiaoyang WangJongwook JangXiaoyi FengJong-Wook LeeXiaoyi ZhangJonhatan SilvaXiaoyu ChenJonne PohjankukkaXiaoyu ZhangJonqlan LinXiaoyuan WangJoo Beom EomXiaoyuan ZhuJoo Chuan YeoXiaoyue NiJoo Hee KimXiaozhen YanJoohwan ChunXiao-Zhou LiuJoon Hyong ChoXiaozhou LüJoon Young KwakXiaozhou ZhouJoongjin KookXibin YangJoon-Jae LeeXie ShengjieJoon-Mo YangXieling ChenJordan G. BarrXihui BianJordan KralevXijun YeJordi CusidoXilin WangJordi Mongay BatallaXilong LiuJordi Palacín RocaXimeng LiuJordi SacristanXiming RenJordi-Roger RibaXin BiJordy ThielenXin ChenJörg FelderXin ChengJörg MiehlingXin FanJörg OpitzXin GaoJörg SchäferXin LiuJorge Antonio Silva CentenoXin LuJorge Bañuelos PrietoXin ShenJorge BrievaXin SunJorge Calvillo-ArbizuXin WangJorge Cerqueiro-PequeñoXin WuJorge E. Ibarra-EsquerXin XiaJorge Ernesto Espinosa OviedoXin ZhangJorge GomezXin ZhengJorge GonçalvesXin ZhongJorge GonzalezXin ZhuangJorge GosalbezXinbao ZhangJorge GuerraXinbin LiJorge Herrera-TapiaXinbing SongJorge L. CandiottiXing ChaoJorge Luis García-AlcarazXing XuanJorge Luis Victória BarbosaXingao ZhangJorge Luis Zambrano-MartinezXingbang YangJorge Maestre VidalXinge YouJorge MárquezXinghao HuJorge Pedro LopesXinghua LiJorge Pérez-GómezXinghui LiJorge PortillaXingliang HuoJorge Ricardo Mejía-SalazarXinglin YuJorge Rodolfo BeingoleaXinglin ZengJorge Rodríguez-ArceXinglong JuJorge TobonXingming ZhengJorge VazquezXingquan CaiJos ElfringXingwang LiJose A. ParedesXingwang ZhangJose A. Rabadan BorgesXingxing ZouJosé A. RoversiXingyu WangJosé A. Siqueira DiasXingyu ZhangJosé Alberto Benítez AndradesXining XuJosé Alberto GonçalvesXinjun WuJose Alejandro Galaviz-AguilarXinli DuJose Alfredo Alvarez-ChavezXinmai YangJosé Antonio Álvarez BermejoXinmin ZhangJosé António C. SantosXinMing ZhangJose Antonio Gonzalez PrietoXinqiang ChenJosé António SimõesXinqiang WangJose Balsa-BarreiroXinqin LiaoJosé C. S. dos SantosXinqun ZhuJose Cabeza-LainezXintian LiuJosé Carlos FonsecaXinxiang ZhangJosé Carlos Palomares-SalasXinyang YuJosé CecílioXinyi RenJose ClaverXinyi TangJosé Cleydson F. SilvaXinyou LiuJosé Cruz Núñez PérezXinyu WangJose De Jesus Agustin Flores CuautleXinyu XueJosé de Jesús Rangel-MagdalenoXinyu ZhangJose Fernando ValenciaXinyue WangJose Francisco Benavente CuevasXinzhe YuanJosé Francisco Gómez AguilarXiong YangJosé Francisco Serrano ClaumarchirantXiongkuo MinJosé GonçalvesXiongYing YeJose GonzálezXiping LiJose Ignacio Lijarcio CarcelXiran CaiJosé Ignacio PagánXisha WangJosé Ignacio Rodríguez MolanoXiufeng HanJosé J. Alonso Del RosarioXiufu ShuaiJose Jaime Camacho-EscotoXiujian LiuJosé Jair Alves Mendes JuniorXiuli WangJosé Joaquín CerónXiupei YangJose JosephXiuyou HanJosé Juan García HernándezXiwei BaiJosé L. HernándezXiwei MiJosé Luis Clúa EspunyXiyu ShiJosé Luis Hernández-HernándezXiyuan ChenJosé Luis OlazagoitiaXizhang GaoJose Luis Outón MéndezXizheng KeJose Luis Pérez-CastrillónXizhong ShenJose Luis Vazquez-AvilaXu ChenJosé Luis Viveros-CeballosXu FengJose M. Del AlamoXu GuoJose M. LlorensXu HeJose M. RamirezXu LexiJosé Machado Da SilvaXu LiangJose Manoel BalthazarXu LongJosé Manuel AlmeidaXu MengJose Manuel Cano GarciaXu WangJosé Manuel Cano-GarcíaXu XuJosé Manuel M. M. de AlmeidaXu YangJosé Manuel MeraXuan MaJosé Manuel TorresXuan SongJose Maria ArmingolXuan Truong NguyenJosé María Garrido BalsellsXuan ZhaoJosé María Martínez-OtzetaXuance ZhouJosé María Vicente-SamperXuan-Ha NguyenJosé MarínXuanwen TaoJosé Martínez-LilloXuanyi DongJosé MaurícioXubing YangJosé MetrôlhoXuchuan JiangJose Miguel Monzon VeronaXudong GeJose Miguel Sanchez BornotXudong JiJosé NevesXudong WangJose NuñezXudong YangJosé Paulo Ferreira LousadoXudong ZhaoJose Paulo MolinXudong ZouJosé Pedro AmaroXue HanJosé Pedro SantosXue LiJose Priego-QuesadaXue QiJosé Ramón Gaxiola-CamachoXuebo ZhangJose Ramón Quevedo PérezXuechao DuanJosé Ramón Rodríguez-PérezXuedan WuJose RangelXuefen ChiJosé Reinaldo SilvaXuefeng DingJosé Roberto CardosoXuefeng ZhangJose Ruiz-PinalesXuefeng ZhouJosé SaenzXuejing WangJose Sebastián Velázquez BlázquezXueju ShenJose SeixasXuening QinJosé Simão Antunes Do CarmoXueru BaiJose V. RieraXuewu LiJose V. Ros-LisXueying WangJosé ValdezXuezhi MaJose Vicente Abellan-NebotXuezhi WangJoseb BlazekXuezhi ZengJosef DobešXufei YangJosef KederXugang LuJosef KollmitzerXuke HuJosef KřečekXukun YinJosefina GutiérrezXumeng ZhangJose-Maria Buades-RubioXunxun ZhangJosemaria Malgosa-SanahujaXuwei ChenJose-Maria Serrano ChicaXuyou LiJosep Carles Benítez-MartínezXuzhou LiJoseph BoydY. B. EismaJoseph DoyleYaarob Al-NidawiJoseph HupyYabin GuoJoseph KvedarYa-Chin KingJoseph P. AlbanesiYachun MaoJoseph P. HornakYacine AchourJoseph Randall MoormanYael NemirovskyJoseph Sunday OjoYaewon KimJosé-Victor RodríguezYafei WangJoshua P. BaileyYagya Raj PandeyaJoshua TroppYahia SaidJosias BatistaYa-huei WangJosip IvšinovićYajun JiangJosip KasaćYakoub BaziJosip MusićYakov RoizinJosko SodaYakov StrelnikerJosué Álvarez BorregoYakun JuJothi Prabha AppaduraiYalew Zelalem JembreJovan S. BajićYali ZhangJovan TrajkovskiYan Chai HumJovanka LukicYan GongJoy Iong-Zong ChenYan HongJoydev GhoshYan KazakovJoyjit ChatterjeeYan LiJoyraj ChakrabortyYan LiuJoze TavcarYan LuJozef BockoYan Naung SoeJózef DrewniakYan ShiJozef GawlikYan V. ZubavichusJozef HusarYan XiangJozef JuhárYan XuJozef KrajnakYan YanJozef KrilekYan ZengJozef MinďášYan ZhangJozef PapanYan ZhaoJozef PeterkaYanan LiangJozsef KatonaYanbin GaoJózsef VásárhelyiYanchao MaoJu LinYanchao SunJu WangYang BuJuan Alberto Besada PortasYang ChenJuan Antonio Castro-GarciaYang ChengJuan Antonio Guerrero-IbáñezYang HuJuan Antonio López RamosYang LiJuan Antonio Martinez NavarroYang LinJuan Antonio Martinez RojasYang LiuJuan Antonio RayasYang LuoJuan Antonio Rojas-QuinteroYang MiJuan Antonio Valera-CaleroYang MiaoJuan Arturo SquellaYang MingJuan BoteroYang PengJuan C. GrandaYang RanJuan C. Infante-MoroYang SongJuan CalderonYang WangJuan Carlos Cuevas MartínezYang WeiJuan Carlos Martínez EspinosaYang XingJuan Carlos OlivaresYang XuJuan Cota-RuizYang YangJuan CruzYang YuJuan David González-RuizYang ZhangJuan De Dios Sanchez-LopezYang ZhouJuan Diego Ania-CastañonYanghanzi ZhangJuan Ernesto Solanes GalbisYangjie SunJuan EspinozaYanglong LuJuan F. PrietoYanglong ZhongJuan Felipe Pérez-Juste AbascalYangong ZhengJuan Francisco ColomaYangquan ChenJuan Francisco Sánchez-PérezYangRong ChenJuan Gabriel Avina-CervantesYang-Wai ChowJuan I. GuerreroYang-Xin YuJuan I. LarruquertYanhu ZhangJuan Irving VasquezYanhua MaJuan José CubillasYanhua ShaoJuan José FernándezYanjie GuoJuan Jose Galiana-MerinoYanjie ZhouJuan José López EscobarYanki AslanJuan Jose Saucedo-DorantesYanli ZhaoJuan LiYanliang ZhangJuan Luis Navarro-MesaYanling SchneiderJuan M. CalderonYanling SunJuan M. VilardyYanmin WangJuan Manuel EscañoYanni YangJuan Manuel GutiérrezYannick BidelJuan Manuel Mayor-TorresYannick VerbelenJuan Monzó-CabreraYanping XuJuan OrtegaYanqun TangJuan P. Domínguez-MoralesYanru LyuJuan P. VasconezYanwei HuangJuan Pablo Amezquita-SanchezYanxiao LiJuan Pablo PascualYanyu XuJuan Pascual-GarcíaYanzhe XuJuan Pedro Muñoz-GeaYao GuoJuan Prado-OlivarezYao LiangJuan Rodrigo Vélez-CorderoYao MaoJuan ShanYao TongJuan Suardíaz MuroYao ZhangJuan TervenYaobing ZhaoJuan-Antonio Cordero-FuertesYaochun ShenJuan-Manuel Belda-LoisYaodong GuJuan-Pablo Ramirez-ParedesYao-Feng ChangJuarez Bento Da SilvaYaogai HuJude HemanthYao-Hsin ChouJudith M. DawesYaohu LeiJudith RosenowYaokai FengJudy AnithaYaolu LiuJue WangYaoming MaJuerg NeuenschwanderYaoran LiuJuhyeon HongYaoxiang LiJuhyeong RyuYaoyu CaoJui-Hung LiuYaping ZhangJui-Pin YangYaqeen Sabah MezaalJui-Yung ChangYaqoob MajeedJulen Castellano PaulisYara AlmubarakJules ThibaultYarjan Abdul SamadJulia KörnerYaser Daanial KhanJulian EvansYash GuptaJulian HothYashwant SinhaJulian L. WebberYasir AliJulian M. Estudillo-AyalaYasir HamidJulian Maria Gonzalez EstevezYasser AlbagoryJulián TomaštíkYassine BouteraaJuliana Aparecida AnochiYassine HimeurJuliana DushanovaYassine MalehJulianne OliveiraYasue MitsukuraJulie WilsonYasuhiro AkiyamaJulien BustilloYasuhiro YoshimuraJulien LesoupleYasunari MatsuzakaJulien MagnienYasushi HayakawaJulien PerchouxYasushi NumataJulien VieillardYasushi SumiJulieta Domínguez-SoberanesYasutaka KitahamaJulio Ariel Hurtado AlegríaYasutoshi MakinoJulio Bastos-ArrietaYasuyuki HirakawaJulio Calleja-GonzálezYat Sze ChoyJulio Cesar De OliveiraYatao RenJulio César Mello-RománYatin WadhawanJulio César Ponce GallegosYau Hee KhoJulio Cesar Rosas CaroYawar AbbasJulio HenriquesYazan A. AlqudahJulius WelzelYazeed Yasin GhadiJumaa Salman ChiadYazhou YuanJumabek AlikhanovYe HeJun Cheng CaoYe LuJun Cong GeYee Kit ChanJun FuYee Loo FooJun GaoYee Loon SumJun Hui ParkYejin KimJun Ki KimYeming ZhangJun LiYen-Da ChenJun LinYen-Kuang LinJun Long LimYenny Villuendas-ReyJun Min SuhYeong Hwan KoJun RenYeong-Hwa ChangJun SuzurikawaYeongin KimJun TakamatsuYeon-Mo YangJun TangYeonwoo LeeJun WangYeou-Jiunn ChenJun WuYeoun-Jae KimJun YanYevhen DavydenkoJun YangYexiang TongJun ZhaYi BaoJun ZhangYi CenJun ZhuYi ChangJun ZouYi HuJunaid AneesYi JiangJunaid ArshadYi LiangJunaid RashidYi QinJunaid ShujaYi SuJunbo ShiYi WangJung Hwan ParkYi XuJung Kim SiYi YangJung Min PakYi ZhangJung Sun YooYi ZhaoJung Tae (Steve) KimYi ZuoJung Woo SohnYiannis KantarosJungang WangYibing LiJung-Dong ParkYi-Bing LinJunghee HanYibo WangJung-Hong MinYichao YuanJunghun ChoiYichao ZhaoJunghwan HanYichen CaiJunghwan HwangYichuan WuJunghyeon OhYide ZhangJunghyuk KoYi-Fei WangJung-Mu KimYifeng GaoJungpyo HongYih JengJungsan ChoYih-Min WuJung-Su KimYihua KangJung-Sup UmYihua TanJunho ChunYihui PanJunho JeongYi-Hung LiaoJunho YeoYi-Jen MonJunhong ParkYijia ZhangJunichi InoueYijie XunJunichi NaganawaYi-Ju TsaiJunjian HuangYike LiJunjie WuYikun YangJunjing DengYilin SongJunjue WangYilun JiangJunjun CaoYilun ZhuJunlei QiYiming HuoJunlong GuoYiming JiJunpeng ShiYiming TangJun-qi XuYin Chai WangJunqing ZhuYin LiJunsik KimYin QiJunwei SunYinan HeJunwen ZhongYinbin MiaoJun-Won HoYing BaiJunxiang ZhuYing CuiJunyan LiuYing HongJunye LiYing LiJunyeong KimYing WangJun-yi SunYing WengJunyi ZhaiYing ZhangJunyue ZhengYing ZhongJunzhou HuoYingchao FengJupeng DingYing-Chieh LiuJuraj BartolicYing-Chih LaiJuraj JagelcakYing-Chih LiaoJuraj KacurYingchun SuJuraj MachajYinghao ZhaoJürgen LorenzYinghui YeJurģis PoriņšYingke LeiJuri TaborriYinglong WuJurica SevaYingqiu ZhouJussi AaltonenYing-Ren ChienJustin AlbertYingtao LiuJustin F. SchneidermanYingxiang LiuJustin R. SempsrottYinsheng ChenJustinas PupeikisYinyin BaoJustus MarquetandYipeng LiuJustyna GrabskaYiping ChenJustyna Patalas-MaliszewskaYiqiang FanJustyna SulejYiqiang ZhanJustyna ZygmuntowiczYi-Qing NiJuvenal Rodriguez-ResendizYiran LiuJuveriya ParmarYiran LuoJuyong LeeYiru WangJyh Miin LinYirui WuJyh-Cheng ChenYitao ZhangJyoti GroverYitian ShaoJyoti Prakash SinghYiting KangJyotir Moy ChatterjeeYiu Yin Raymond LeeJyun-Jie LinYiwen HuangK. R. ShobhaYiwen ShenKa Io WongYixin HeKa KimYixin LiuKaan KalkanYixin ZhangKaan OrhanYixiong HeKadir SabancıYixiong ZhangKadiyala RamanaYixuan FengKaharudin Bin DimyatiYixuan WangKah-Hui WongYiyang ChenKai ChengYiyi HuangKai DongYiyu TanKai GuoYizhen PengKai HeYizhi SongKai HuYizhi ZhangKai HuangYizhong WangKai LiuYoav BrozaKai PengYogeenth KumaresanKai SunYogendra Kumar MishraKai WangYogesh ShrivastavaKai WeiYoghitha RamamoorthiKai WuYohei MatsumotoKai XiongYoko SuzukiKai XuYolanda Castillo-EscarioKai YangYolanda GuerreroKai ZhangYomna MahmoudKai ZhouYong ChenKaibao NieYong-Cheng WangKai-Chun LiuYong HeKaida XiaoYong HuangKaidong WangYong Ju JungKaifeng LiuYong KimKaige WuYong Kyoung YooKaihao ZhangYong LiKai-Hsiang HuangYong PangKaikai XuYong SangKaimeng DingYong Suk ChoiKais BelwafiYong WanKaisu LankinenYong YangKaitao LiYong ZhangKaixian BaYongchuan TangKaiyan QiuYongchun ZhengKalambuka Hudson AngeyoYongcong ShaoKalamullah RamliYongding TianKalathur SanthanamYongfei FengKaleeswaran BalasubramaniamYonggang XiaoKalia OrphanouYong-Guk KimKalle KärhäYonghao XuKalliopi KravariYonghong ChenKalliopi KyriakouYonghoon LeeKalpana BhattYonghwan JeongKalpana SettuYongjia XuKalyan GhoshYong-Jian HuKalyanasundaram MadhuYongjie ZhaiKam NedevYong-jin JeongKamal ChapagainYongjin SungKamal GuedriYong-Joo ChungKamal KayedYong-joo KimKamal KishorYong-Joong LeeKamarudin AmbakYongjun JiaKamarul Hawari Bin GhazaliYongjun LiuKambiz TehraniYongjun PanKamel MarsYongjun RenKamel SultanYongjun ZhangKamil BorawskiYongkuk LeeKamil DimililerYongkun SuiKamil GareevYongle PanKamil KrasuskiYong-Long LinKamil KrzywinskiYongqing HeKamil LeksyckiYongsheng ZhangKamil StaniecYongsu ParkKamil ŽidekYongsub LimKamran IqbalYongwoo JangKamran ShaukatYongxin LiuKamran SiddiqueYongzeng YangKamrul H. FoysalYongzhi MinKan LiYoon-Soo JangKanagachidambaresan RamasubramanianYosef PinhasiKandala N. V. P. S. RajeshYoshiaki AdachiKang LiuYoshifumi NishidaKang ZhouYoshihisa YamaokaKang-Chun PengYoshikazu KoikeKanghyeok LeeYoshikazu WashizawaKangjian HeYoshinari KamakuraKangli LiuYoshito WatanabeKanji OnoYosi KristianKanokwan SitthithakerngkietYosio ShimabukuroKanstantsin MiatliukYossi HadadKaori FujinamiYosuke SugiokaKapassa EvgeniaYosuke TomitaKapil DebnathYou LiKapil GulatiYoubin ZhengKara PetersYoucai ZhaoKaraca UtkuYouchung ChungKarekin EsmeryanYoufa LiuKarel CharvatYougang BianKarel PalečekYoung ChaKarel PavelkaYoung Chul LeeKarel RoubikYoung Deok ParkKaren GordonYoung Do JungKari SystäYoung Min SongKarim AlameYoung Soo SuhKarim AliakbariYoung Wook LeeKarim AlyYoungbae HwangKarim GasmiYoung-Chan JangKarim HamzaYoungchul BaeKarim LounisYoung-Dam KimKarim S. KarimYoung-Geun ParkKarin Anna HummelYounggun ChoKarin KolloYoung-Hak ChoKarina LebelYoung-Ho ChoKarla De JesusYoungho KimKarla HillerYoung-Ho KoKarla MossiYoung-Jae RyooKarol AbratkiewiczYoung-Jin ParkKarol MiądlickiYoung-Joo SuhKarol PiniarskiYoungmin ChooKarolina FurtakYoungsun KongKarolina Kołczyk-SiedleckaYoungsun RyuhKarolina Krzykowska-PiotrowskaYoungtai ChoiKarolína ŠiškováYoungwook SeoKaroline K. (Johnson) BarkjohnYoun-Sik HongKarolj SkalaYousaf Bin ZikriaKarsten WittYousef KassemKarthik KannanYousef-Awwad DaraghmiKarthik PushpavanamYou-Shyang ChenKarthik RamamurthyYoussef ErramiKarthikeyan PeriyasamiYoussef Es-SaadyKarumbaiah ChappandaYouwen ZhangKarumbaiah N. ChappandaYouye XieKashif AliYu (Eve) ZhangKashif ElahiYu CherepennikovKashif SaleemYu FuKashish DhalYu GongKaspar M. B. JansenYu GuoKaspar StaubYu HouKaspars KroicsYu HuangKasumawati LiasYu JiangKatarina ItrićYu LiuKatarzyna AntoszYu SongKatarzyna BialasYu TaoKatarzyna HarezlakYu Ting TsaiKatarzyna MajewskaYu V. BolotinKatarzyna Mańka-MalaraYu WuKatarzyna Nowakowska-LipiecYu XuKatarzyna PanasiukYu ZhangKatarzyna RobaszkiewiczYuan CaoKatarzyna StaszakYuan F. ZhengKateřina BerkováYuan LaiKaterina TzafilkouYuan LinKatherine Brooke-WavellYuan MengKatherine ShanksYuan NieKathiravan SrinivasanYuan ShenKathryn L. HavensYuan WangKatie HellierYuan XuKatina KralevskaYuan YuanKatrin SchmittYuan-Chang LiangKatya ArquillaYuan-Fong Chou ChauKaushik MandalYuanhang ShaoKaushik SridharYuan-Jen ChangKaveh Farokhi SadabadiYuanjun GuoKaveh MoulaeeYuankai QiKa-veng YuenYuankun LiuKawachi TetsuyaYuankun XuKay RobbinsYuanlong XieKazem PourhosseinYuanpeng WuKazem Reza KashyzadehYuanquan WangKazimierz BecekYuanrong LuKazimierz FabisiakYuan-Sen YangKazimierz GutYuanwei WuKazuhiro HondaYuanxi SunKazuhiro TakahashiYuanxin YeKazuki IkedaYuanyuan ShaoKazuo KumamotoYuanzhu ChenKazuyuki MatsumotoYubin GuoKe FengYubing HuKe LiYuchen JiangKe MaYuchen XuKe ShaoYuchen ZhengKe SuYu-Cheng ChouKe TianYu-Cheng FanKe WangYu-Cheng WangKe XuYucheng WuKechen SongYu-Chi LeeKee-Ho YuYu-Chi PuKee-Won KimYudi FernandoKefei LiuYudi PrayudiKefeng GuoYudong LiuKeh-Chyang LeouYue GuKeisuke KusakaYue WangKeisuke MukaiYue YuKeisuke YagiYue ZhangKeisuke YonedaYue ZhuoKeita YasutomiYuecheng ShenKeith SchubertYuejian ChenKejin WangYueru XuKele XuYue-Shan ChangKelly Cristina StéfaniYueting ZhangKelly De JesusYueyang BenKelly E. JohnsonYueyang LiKemal AkkayaYufei LiuKemal Maulana AlhasaYufei MaKen SasakiYufridin WahabKen′ichi KoyanagiYuguang FuKeng-Hao LiuYuguo CuiKenichi HayashizakiYuguo LiKen-ichi NomuraYuhan HuangKenichi OguchiYuhao QiangKenichi YamadaYuh-Chung LinKenji AonoYu-Hen HuKenji HayashidaYuhong ZhengKenji KawashimaYu-Hsi HuangKenji SatouYuji DongKenji ShimazoeYu-Jie TanKenji UchiyamaYujie WangKennedy O. OkokpujieYu-Jui FanKenneth A. LoparoYujun MaKenneth G. GerowYu-Jung HuangKenneth LeitchYuki KojimaKenneth OkeduYuki OkamotoKenneth ScheplerYukiharu HisakiKenneth SundarajYukihide NishimuraKenshi SahoYukio YamadaKenshin TakemuraYukyung ChoiKensuke NakamuraYulei QianKent RosserYulia O. BobrovaKerim ÇiçekYulian CaoKerim Kürşat ÇevikYuling NiuKerlos Atia AbdalmalakYulong LiKerry Brian WalshYun Jung HeoKerstin JohansenYun KongKerstin ThurowYun LinKeshav KumarYun LiuKeshav SoodYun PanKesheng WuYun Seon DoKetan KotechaYun ShimKetan PolYun Soung KimKetson Roberto Maximiano Dos SantosYun WangKeun Ho RyuYun XuKeunhwa LeeYun ZhuKevin BlackYuna MaoKe-Vin ChangYunbin YuanKevin ChauYunchao JiangKevin DesaiYunchao TangKevin G. Montero QuispeYundong ZhangKevin McCarthyYunfei ChangKevin MullaneyYun-Feng XiaoKevin WillyYung Hsiang ChenKeyang WuYung Po TsangKeyi WangYung Sheng ChenKeyvan RamezanpourYungang CaoKezheng LiYung-Chou KaoKhadak Singh BhandariYung-Chung ChuangKhai Wah KhawYung-Fa HuangKhairi AbdulrahimYung-Hui LiKhairul Nizam Abdul MauludYung-Ping LiuKhairullo Faizullaevich MakhmudovYung-Tsan JouKhairunnisa HasikinYunhe ZhaoKhaled ArdahYunhee KangKhaled Ben KhalifaYun-Ho ShinKhaled H. AlyoubiYun-Hsin ChuangKhaled KaânicheYunhua RaoKhaled R. AhmedYunhui ZhangKhaled RabieYun-Kyu AnKhaled RiadYunlong WangKhaled ZennirYunlong ZhangKhalfalla AwedatYun-Ping SunKhalid Adnan AlissaYunping YangKhalid ArifYunqian DaiKhalid ElgazzarYunshan SunKhalid HashimYun-Sik NamKhalid MasoodYunsong PengKhalid MehmoodYuntao GuoKhalid Nazim Abdus SattarYunus EgiKhalid RazaYunus Ziya KayaKhalid SaeedYunwei DongKhalid SayoodYunwen TaoKhalid YahyaYunyang YeKhalil A. KhatriYunyoung NamKhalil IbrahimiYunze HeKhalil TamersitYuqi JinKhalil UllahYuqi LiKhalisanni KhalidYuqi LiuKhan Rana Muhammad AsadYuqian HuKhandaker Foysal HaqueYuqian ZhangKhawaja Khalid MehmoodYuri KonstantinovKhayyam MasoodYuri KrakKhian-Hooi ChewYuri PisarevskyKhizar HayatYuri RzhanovKhoa NguyenYuri V. FilatovKhrissy MedeirosYurii BaryshevKhuong An NguyenYurii E. GeintsKhurram HameedYurikov AlexeyKhurram Karim QureshiYuriy G. DenisenkoKhurshid JahanYuriy KuleshovKhushboo MunirYuriy L. OrlovKi H. ChonYuriy L. Vasil′evKi-Baek LeeYuriy RomanetsKien DinhYuriy SyerovKien Voon KongYury KapelyushinKiho LimYury NikitinKijoon LeeYury YasyukevichKillol Vishnuprasad PandyaYu-Seop KimKim BoströmYusheng LinKim CluffYusheng ShiKim-Fung TsangYushuai LiKim-Hung LeYu-Shun YangKinam KimYusuf PerwejKinan GhanemYusuf YesilceKing Fai HungYusuke FujitaKinga Kostrakiewicz-GierałtYusuke KajiwaraKinga MártonYusuke MukutaKinya TorideYusuke OnoKinzo KishidaYuta TerasakaKiril AlexievYuta TsubouchiKirill Andreevich KuznetsovYutao LiuKirill CherednichenkoYutian PangKirk ShelleyYuting GuoKishor Datta GuptaYuting WanKishor Kumar SadasivuniYuvaraj M. HungeKishore DebnathYuvaraj NatarajanKiwon ParkYuvraj GajpalKiyotaka FujisakiYuwen CaoKiyotaka SasagawaYuxi XieKjartan HalvorsenYuxiang PengKlaudiusz GrübelYuxin PanKleomenis KalogeropoulosYuxin XingKlimek KamilaYuxin ZhangKoay Kuan ChuangYuyang GuKobra HeidarbeigiYuyang LiKo-Chiu WuYuyang ZhangKodai KitagawaYuzhe LiuKogularasu SakthivelYuzhu WangKoichi KuritaYuzo IanoKoji FujitaYvetta VelískováKomarizadehasl SeyedmiladZacarias PraviaKonda Srinivasa RaoZackory EricksonKonrad FurmańczykZafar BalochKonrad WaluśZaher Al BarakehKonrad WojtowiczZahid KhanKonstantin A. LukyanovZahid UllahKonstantin BulatovZahir M. HussainKonstantin GolantZahra EinalouKonstantin I. KostromitinZahra LotfollahiKonstantin Johannes ScholzZahra Shams EsfandabadiKonstantin KorotkovZahra SheikhbahaeeKonstantin KozlovZaid Abdi Alkareem AlyasseriKonstantin NikolaevZaide DuranKonstantin P. GaikovichZaihua DuanKonstantin V. KhishchenkoZain-Aldeen A. RahmanKonstantinos A. TsintotasZakariya AlgamalKonstantinos DelimpasisZakriya MohammedKonstantinos DemertzisZamora-Polo FranciscoKonstantinos DemestichasZan LiKonstantinos DovelosZan NieKonstantinos FysarakisZarah MoussaviKonstantinos G. NikolakopoulosZareena KausarKonstantinos GiannakisZargari Marandi RamtinKonstantinos KarampidisZatelli Maicon RafaelKonstantinos KotisZawar HussainKonstantinos LimniotisZawar Hussain KhanKonstantinos M. GiannoutakisZawar ShahKonstantinos MaliatsosZbigniew BanaszakKonstantinos MisiakosZbigniew DziongKonstantinos NinikasZbigniew KuleszaKonstantinos OikonomouZbigniew LeonowiczKonstantinos ProkopidisZbigniew LoziaKooktae LeeZbigniew NadolnyKoorosh AslansefatZbigniew OmiotekKosin ChamnongthaiZbigniew OtrembaKosmas KritsisZbigniew PilatKostas NizamisZbigniew PilchKostiantyn TorokhtiiZbigniew RanachowskiKosuke FukuiZbigniew UsarekKosuke HayashiZbigniew ZakrzewskiKosuke SanadaZbigniew ZembatyKota KodamaZbigniew ZielinskiKouji AdachiZbigniew ZiembikKoumantakis GeorgeZbyněk KocurKourosh KianiZbyněk RaidaKovadlo Pavel GavrilovichZdena DobesovaKowalski DariuszZdeněk KrňoulKo-Wei HuangZdenek KubikKrasimira Borislavova IvanovaZdeněk NeusserKresimir DolicZdenek SlaninaKrishan KumarZdzisław SroczyńskiKrishna C. MandalZebensui Morales-ReyesKrishna KantZechen YuKrishna KumarZedong NieKrishna Kumar MohbeyZeeshan PervezKrishnamurthy PrabakarZeev VolkovichKrishnamurthy VemuruZehao HeKristen M. MeiburgerZekuan YuKristen MorieZelei ChengKristian DokicŽeljen TrpovskiKristian KostalŽeljka CvejićKristijan LenacZeljko DespotovicKristína MachováŽeljko DolijanovićKristína ZgodavováZeljko HocenskiKristof KippŽeljko KanovićKrithika Latha BhaskaranZeljko SantosiKriti BhargavaZeljko ZilicKruno MilicevicZelun WangKrunoslav JuraicZemin ZhangKrystian ErwińskiZeming FanKrystian MistewiczZeming SunKrystian RadlakZeng-Ding LiuKrzysztof B. BecZenghui LiuKrzysztof BarteckiZengxia MeiKrzysztof ChwastekZeno GeradtsKrzysztof CiecielągZenon NieckarzKrzysztof DamaziakZenon SzczepaniakKrzysztof GrudzieńZepeng LiKrzysztof J. KurzydłowskiZepeng LiuKrzysztof JamroziakZesheng ChenKrzysztof JaskólskiZexi LiangKrzysztof KlimaszewskiZeynep TeklerKrzysztof KluzaZhan WangKrzysztof KoszelaZhan ZhaoKrzysztof KubiczekZhanat KappassovKrzysztof LewandowskiZhang MingleiKrzysztof LowczowskiZhanjun WuKrzysztof NausZhanjun ZhangKrzysztof NawaraZhanlin JiKrzysztof NykaZhanwen LiuKrzysztof ParczewskiZhan-Yi HuKrzysztof PrzybyłZhao ErfengKrzysztof RogowskiZhao HaoKrzysztof SawickiZhao HuangKrzysztof SchabowiczZhao RenKrzysztof SkarżyńskiZhao YangKrzysztof StankiewiczZhao ZhenKrzysztof SzwajkaZhaocheng WangKrzysztof SzymkiewiczZhaodong WangKrzysztof TomczykZhaogang ShuKrzysztof WalkowiakZhaoguo XueKrzysztof WesolowskiZhaohui XueKrzysztof ZubelZhaokun (Brandon) ZhangKrzysztof ŻywickiZhaoliang ZengKsawery KrencZhaolong YuKsenia OstrowskaZhaomeng LiuKuan HuangZhaoqiang ChuKuang Yao LoZhaoxiang ZhangKuang ZhangZhaoyang DingKuang-Hsuan ChenZhaoye QinKuanglu YuZhaoyun DingKuen-Suan ChenZhe BaiKumar HarendraZhe ChenKumar RamamoorthyZhe GuoKumar ShivamZhe LiKumar YelamarthiZhe MinKun GaoZhe WangKun JiaZhe ZhouKun LiuZhechao QuKun WangZhedong ZhengKunal PalZhen QiuKundan SivashanmuganZhen WangKun-Hui ChenZhen YuanKunkun FuZhen ZhangKunlin ChenZhenbing ZhaoKun-Lin LeeZhenchao TangKunook ChungZhendong WangKunpeng YaoZhenfeng GongKuo-Kun TsengZheng BinKuo-Liang HuangZheng CaoKurita KazunariZheng LiuKurt JacksonZheng TongKuruva LakshmannaZheng WangKutalmis SaylamZheng XuKwang Hee WonZheng Zachory WeiKwang Woo NamZheng ZhangKwangho KoZhengang LuKwang-Sig LeeZhengbin LiKwangsup ShinZhengchao ChenKwi-Il ParkZhengfei DaiKwok Kan SoZhengguo LiKwok Tai ChuiZhenghong YuKwok-Ping ChanZhenglin YangKyandoghere KyamakyaZhengqing ZhouKyeong HurZhengran HeKye-Si KwonZhenguo NieKyi TharZhenguo NiuKyle DunnoZhengwei LiKyle R. WiltZheng-Yi FengKymberly YoungZhengyuan ZhouKyohei HisanoZhenhu LiangKyoko HasegawaZhenhua LiKyra KaneZhen-Hua TangKyriaki-Nefeli D. MalamakiZhenhua TianKyu Tae ParkZhenhuan YiKyu-Haeng LeeZhenping QiangKyun Kyu KimZhenwei LiangKyunghoon MinZhenwen HeKyung-Hwan KwakZhenxu BaiKyung-Hyun SuhZhenyu ChuKyungjin AnZhenyu HeKyungkwang JooZhenyu TanKyung-Sook ChoiZhenyu YuanKyung-Soon ParkZhenyu ZhouKyungwoon LeeZhenzhong KuangKyungyong ChungZhenzhu WangL. J. NickischZheqi YuL′udmila DulebováZhetao ZhangLa LiZheyuan ChenLaavanya RachakondaZhezhuang XuLachit DuttaZhi HeLadislav FőzőZhi LinLadislav PilařZhi NingLadislav PolakZhi QiaoLahoucine HanichZhi WangLaith A. SabriZhi YangLaith AbualigahZhibin ZhaoLaith AlzubaidZhibo WangLaith T. KhraisZhichao CaoLajos DarócziZhicheng YangLakshmi Shankar IyerZhidong XueLal HussainZhifan GaoLala H. RajaoarisoaZhifu LiuLaleh AvazpourZhigang ChenLalitha GummidiZhigang JiLam PhamZhigang LiLamberto TronchinZhiguang WangLance E. ChristensenZhiguo DengLang XuZhiguo LuLangping LiZhiguo MengLanto RasolofondraibeZhihao ZhouLanxiang SunZhihong DengLapo BoschiZhihong ZhangLapo MiccinesiZhihua XuLara Del ValZhihui GuoLara RebaioliZhihui LiLara WarmelinkZhijian ChenLaramie PottsZhijian XieLarisa BeilinaZhijun WuLarisa DunaiZhiJun YanLarry AbelZhikang LiLars O. GrobeZhikang YuanLarysa TitarenkoZhilai LuLászló BeneZhi-Li ZhangLászló CsambalikZhilong SongLászló Csurgai-HorváthZhilu WangLászló DudásZhimeng YinLászló GogolákZhiMing ZhangLászló SiposZhipei HuangLászló SujbertZhipeng CaiLatif U. KhanZhipeng LaiLaura AngiolettiZhiping LinLaura BragonzoniZhiqiang CaoLaura BurattiniZhiqiang FanLaura DossiZhiqin ChuLaura FarinaZhisheng GaoLaura GarcíaZhitao GuanLaura GastaldiZhitao WangLaura JasińskaZhitian ShiLaura MicheliZhi-Ting YeLaura Parra-AnguitaZhiwei GuoLaura PeraltaZhiwei LuLaura PierucciZhiwei MenLaura Rodriguez-LorenzoZhiwei ZhangLaura RomeoZhixin ZhanLaura RuseZhixiong LiLaura TascónZhixiong ZengLaura TolesZhiyi HeLaura Vázquez-AraújoZhiyi TangLaura WallardZhiyong YangLaura-Diana RaduZhiyong ZhaoLaurel O. SillerudZhiyu ShengLaurent BeaudoinZhiyuan FengLaurent CormierZhiyuan LiLaurent DonzéZhiyuan RenLaurent SpinelleZhiyuan ZhaLaurențiu MiliciZhiyuan ZhuLauri LovénZhizhen LiuLauri SydänheimoZhizhong DingLaurindo Antônio GuasselliZhizhong TongLawrence LauZhongbao WeiLaxman SinghZhongchao LiangLaxminarayan SahooZhongguo LiLazhar KherijiZhong-Hua PangLazo M. ManojlovićZhonghua SunLazuardi UmarlZhonghua XieLe Huy TrinhZhongjing RenLe Minh Tu PhanZhongjun DingLe Nguyen Quoc KhanhZhongliang LiLeandro CoelhoZhongliang QiaoLeandro CruzZhongqi MiaoLeandro FernandesZhongqiang HuLeandro N. BalicoZhongshan JiangLech Bolesław DobrzańskiZhongwei HuLee F. JohnsonZhongxing ZhangLee Jen-ChunZhongxu HuLee-Yaw LinZhongyuan FangLefteris BenosZhongyue ZhangLei ChenZhongzheng LiuLei ChuZhou GuLei GaoZhoujie WuLei HangZhouxu ChenLei HouZhouzheng GaoLei JingZhuangjian LiuLei KangZhu-Jun GuLei LiZhun ZhongLei LiuZhuo ChenLei MaoZhuo LiuLei PanZhuo WangLei RenZhuofu LiuLei ShiZhuoqi ChengLei SunZhuoran DangLei WangZhuyun ChenLei XuZhuyun XiaoLei XueZhwan Dilshad Ibrahim SktaniLei YuZiad Aeyad TahaLei YuanZian Cheak TiuLei ZhangZidan GongLei ZhuZiemowit DworakowskiLeif KariZifei LiangLeijian YuŽiga KozincLeilei LiZigang DengLele LuanZiguang JiaLena K. L. OestreichZihan LinLenka LhotskaZihao OuLenny Van Erp-van der KooijZihuai LinLeo K. ChengZijian QiaoLéo MendiboureZijie ZhuLeo MrsicZilu LiangLeobardo HernandezZima BeataLeocundo Aguilar NoriegaZine El Abiddine FellahLeon AbelmannZining YangLeon JacobseZinovi DashevskyLeon RothkrantzZinovia KefalopoulouLeonardo Acho ZuppaZirui LiLeonardo Alexandre Peyré-TartarugaZisis TsiatsikasLeonardo Azael García-GarcíaZissis SamarasLeonardo ChiattiZitong YuLeonardo CiaccheriŽivko BojovićLeonardo D′AcquistoZiwen LiuLeonardo Garcia-GarciaZixin WangLeonardo J. ValdiviaZiyang MengLeonardo OrnellaZiyang ZhangLeonardo RundoZiyin WuLeonardo Sastoque PinillaZiyuan PuLeonel Paredes-MadridZiyun DingLeonid FetisovZizung YoonLeonid SeleznevZlatin ZlatevLeonid ZotovZlatko BundaloLeonidas MiliosZofia WróbelLeopoldo Altamirano-RoblesZohaib LatifLeopoldo AngrisaniZohair Al-AmeenLeslie Chavez-GalanZohra BenzartiLeslie Ching Ow TiongZoka B. MilanLesya AnishchenkoZoltán FeketeLeszek AmbroziakZoltan JuhaszLeszek BlaszkiewiczZoltán TamusLeszek BryjaZong QinLeszek DrabikZongan LiLeszek NowosielskiZongliang WangLetitia MireaZongyao ShaLetizia CataliniZongyin YangLetizia De mariaZoran CicaLev KazakovtsevZoran DuricLev KuzminZoran LevnajicLev V. EppelbaumZoran MijicLevente CzumbilZoran StevićLewis NkenyereyeZoubair BoulahiaLi BingZouhair HananiLi HuiZrinka MihaljevićLi JinZsanett S. BodorLi JuZsófia BajiLi LiZsolt TóthLi LinZsuzsanna MiklósLi LiuZuhair JamainLi SuZuhairiah Zainal AbidinLi SunZulfiqar KhanLi XiaZulqurnain SabirLi ZhengZunsong YangLi ZhuZuo-Min TsaiLia E. AciuZuzana MarcalikovaLian NedelchevZuzana VišňovcováLian Sheng WangZvi RothLiana C. SalanțăZvonimir ŠipušLianbo GuoZvonko RadosavljevicLianbo MaŽydrūnas KavaliauskasLiang FanZygfryd WitkiewiczLiang Huang


